# A Proposed Diagnostic Algorithm for Inborn Errors of Metabolism Presenting With Movements Disorders

**DOI:** 10.3389/fneur.2020.582160

**Published:** 2020-11-13

**Authors:** Juan Darío Ortigoza-Escobar

**Affiliations:** Movement Disorders Unit, Institut de Recerca Sant Joan de Déu, CIBERER-ISCIII and European Reference Network for Rare Neurological Diseases (ERN-RND), Barcelona, Spain

**Keywords:** dystonia, inborn error of metabolism, ataxia, myoclonus, chorea, athetosis, hypokinetic rigid syndrome

## Abstract

Inherited metabolic diseases or inborn errors of metabolism frequently manifest with both hyperkinetic (dystonia, chorea, myoclonus, ataxia, tremor, etc.) and hypokinetic (rigid-akinetic syndrome) movement disorders. The diagnosis of these diseases is in many cases difficult, because the same movement disorder can be caused by several diseases. Through a literature review, two hundred and thirty one inborn errors of metabolism presenting with movement disorders have been identified. Fifty-one percent of these diseases exhibits two or more movement disorders, of which ataxia and dystonia are the most frequent. Taking into account the wide range of these disorders, a methodical evaluation system needs to be stablished. This work proposes a six-step diagnostic algorithm for the identification of inborn errors of metabolism presenting with movement disorders comprising red flags, characterization of the movement disorders phenotype (type of movement disorder, age and nature of onset, distribution and temporal pattern) and other neurological and non-neurological signs, minimal biochemical investigation to diagnose treatable diseases, radiological patterns, genetic testing and ultimately, symptomatic, and disease-specific treatment. As a strong action, it is emphasized not to miss any treatable inborn error of metabolism through the algorithm.

## Introduction

Inborn errors of metabolism (IEMs) are defined as any condition that leads to a disturbance of a metabolic pathway, irrespective of whether it is associated with abnormalities in biochemical laboratory tests. Indeed, IEMs include not only enzymes or transporters deficiencies or superactivities, but also chaperons and transcription factors abnormalities. Recently, a current nosology by Ferreira et al., defines 1,015 IEMs in 130 groups (1).

IEMs encompass a large group of single gene disorders that can affect all organs and lead to a variety of symptoms (2). Most of the IEMs are multisystem diseases with neurological (intellectual disability/developmental delay, cognitive regression, hypotonia, spasticity, neuropathy, vision and hearing impairment, encephalopathy/stroke, epilepsy, etc.) and non-neurological (failure to thrive, vomiting, hepatomegaly, splenomegaly, renal tubular acidosis, nephrolithiasis, etc.) manifestations. Commonly initial symptoms and signs of IEMs are somewhat nonspecific (3). Movement disorders (MD) are among the most usual neurological symptoms in children with IEMs and account for a significant part of the morbidity and mortality. MD manifest in IEMs that cause diffuse CNS or selective basal ganglia involvement. As it is known, the basal ganglia participate in the control of voluntary movement and on the other hand this brain area is especially vulnerable to certain IEMs as metal storage defects, energy metabolism and lysosomal storages disorders (4, 5).

A central problem is the large number and variety of IEMs with MD and the poor recognition of these disorders that make it challenging for the pediatric neurologist to decide on the initial evaluation with the consequent delay in diagnosis and timely treatment. This work herein facilitates an update information with new IEMs that present MD, suggests red flags and diagnostics clues for suspecting IEMs, proposes the minimum biochemical studies as stated in each MD and the differential diagnoses according to the neuroradiological findings and provides evidence on symptomatic or disease specific-treatment through a six-step algorithm.

## Overview of Movement Disorders in Inborn Errors of Metabolism

Based on the distinct pathway involved, IEMs can be categorized as described by the nosology of Ferreira et al. into the following groups (1): (1) Disorders of nitrogen-containing compounds, (2) Disorders of vitamins, cofactors, metals, and minerals, (3) Disorders of carbohydrates, (4) Mitochondrial disorders of energy metabolism, (5) Disorders of lipids, (6) Disorders of Tetrapyrroles, (7) Storage disorders, (8) Disorders of peroxisome and oxalate, and (9) Congenital disorders of glycosylation. This accurate and updated nosology will be applied in this review, in which a total of 231 IEMs presenting with MD will be included. The search strategy, the selection criteria and the data extraction applied are described in Supplementary File 1. The IEMs presenting with MD appear in Supplementary Table 1.

Whereas, MD are classified into two main categories, hyperkinetic, and hypokinetic movements. Hyperkinetic MD are unwanted or excess movements which include dystonia, choreoathetosis, tremor, myoclonus, tics, and stereotypies. Definition and classification of pediatric hyperkinetic movements were described by Sanger et al. (6), although; this classification did not include ataxia. Hypokinetic movements are called hypokinetic-rigid syndrome or Parkinsonism (7). Both hyperkinetic and hypokinetic MD are crucial clinical findings with significant implications for diagnosis and treatment.

MD in the context of IEMs are generally a combination of different MD, therefore a MD oftentimes does not predict the type of IEM. The 51% of IEMs of this review presents 2 or more MD. In this group there are well-known diseases namely neurotransmitters disorders (8), cerebrotendinous xanthomatosis (9), Wilson disease (10), POLG deficiency (11), Niemann Pick disease type C (12), and Glucose transporter 1 deficiency (13) that can exhibit virtually any MD and that must always be incorporated in the differential diagnosis on either child with MD. It should be noted that many of these referred IEMs are also treatable.

**Figure 1:**
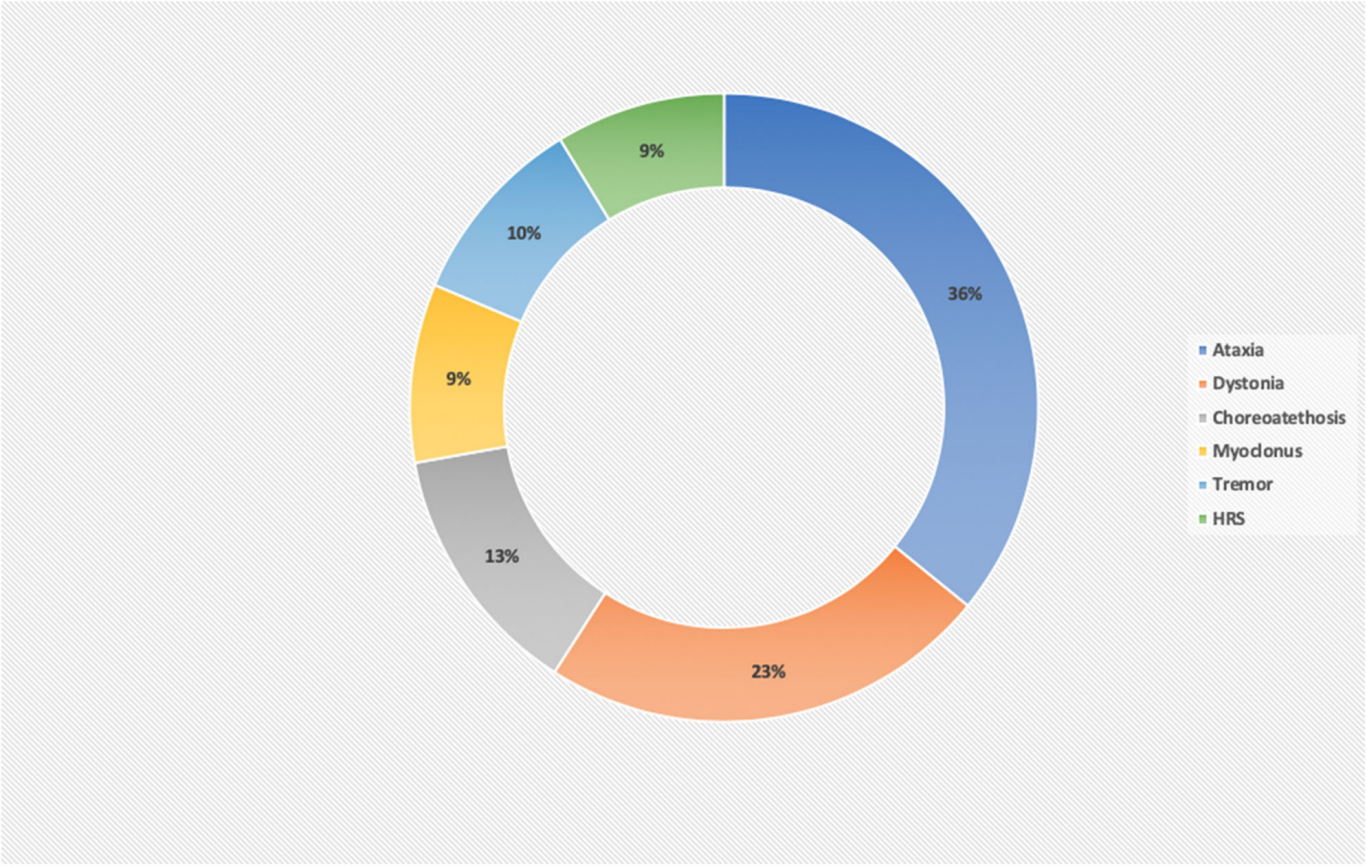
Percentage of movement disorders in a series of 231 inborn error of metabolism disorders. Ataxia and dystonia are the most frequent MDs in IEMs, comprising about 60% of the disorders. HRS and myoclonus are the least frequent MD and for this reason, the most useful MD in guiding the diagnosis of a definite IEM.

Rarely, there is an isolated MD, commonly mild ataxia or chorea, as part of a more diffuse clinical picture. Furthermore, the MD can evolve over a period of time in the same patient, by the progress in the severity of the main MD [e.g., dystonia in *PANK2*-Pantothenate kinase-associated neurodegeneration (14), *FA2H*-Fatty acid hydroxylase-associated neurodegeneration (15), *SLC39A14* deficiency (16), *TIMM8A*-Deafness-Dystonia-Optic Neuronopathy Syndrome (17), or ataxia in COQ8A deficiency (18), *MVK*-mevalonate kinase deficiency (19), *SNX14* deficiency (20), and *COG5*-CDG (21) or by presenting new further MDs], in particular HRS in relation to neurodegeneration [e.g., Leigh syndrome (22), CLN1 (23), *PLA2G6*-associated neurodegeneration (24), *WDR45*-associated neurodegeneration (25), Tay Sachs or Sandhoff disease (26), and L-2-hydroxyglutarate Dehydrogenase deficiency (27)]. Ataxia and dystonia together represent >50% of the cases of MD, with myoclonus and hypokinetic rigid syndrome being the least frequent MD. The percentages of the different MD in the IEMs are detailed in Figure 1.

Several studies have shown that IEMs account for less than a quarter of diagnoses in children with MD, for this reason timely recognition of these underlying IEMs often allows disease-specific treatments and the best possible outcome for the affected child (3, 28). The percentage of diagnosis reached depends on the population included and on the biochemical or genetics analysis conducted. Diagnosis rate is lower in children with acute symptoms and substantially higher in children with chronic pathology. In this review, six studies investigating IEMs presenting with MD in 909 children and adults have been found (3, 29–33). The groups examined were very heterogeneous while other study exclusively included patients with dystonia (29). When all movement disorders were included, dystonia and ataxia were the most frequent MD. Most of the studies applied targeted next generation sequencing panels obtaining a diagnostic yield ranged between 11 and 51%. Nevertheless, among three other studies in children with acute pathology presenting to the pediatric emergency department, the percentage of children with IEMs was between two and eight percent of all cases. These cases included IEMs as various as ceroidolipofuscinosis, mucopolisacaridosis, pantothenate kinase-associated neurodegeneration, glutaric aciduria type 1, MELAS, and Leigh syndrome (34–36). The full-description of all these studies is displayed in Supplementary Table 2.

Regrettably, there are only a few studies that make a comprehensive description of the MD in the IEMs and most of the knowledge is based on individual cases or series of very few patients. Quite apart from the fact that many of these report do not provide in-depth characterization of the MD. Some studies that include large series of patients with IEMs and MD are: Tetrahydrobiopterin (BH4) deficiencies (37), cobalamin-related remethylation disorders (38), SERAC1 deficiency (39), mitochondrial disorders (22), PLA2G6-associated neurodegeneration (24), lysosomal storage disorders (40), cerebrotendinous xanthomatosis (9), and congenital disorders of glycosylation (41). IEMs with MD as a primary or prominent feature and treatable causes should always be in mind. These groups of IEMs are listed in Table 1.

**Table 1:** Movement disorders and neurological/non-neurological characterization, biochemical findings, neuroimaging, and disease-specific treatment in IEMs with MD as a prominent feature.

**Diseases**	**Gene (omim)**	**MD phenotype**	**Other clinical features**	**Biochemical findings**	**Neuroimaging**	**Specific treatment**	**References**
**DISORDERS OF NITROGEN-CONTAINING COMPOUNDS**							
Guanidinoacetate methyltransferase deficiency	*GAMT* (#612736)	Dystonia and choreoathetosis	ID, hypotonia, S, hyperreflexia	↓CSF creatine, ↓CSF creatinine, ↓ creatine excretion	BG hyperintensities	Creatine and ornithine supplementation, arginine restriction	(42)
Creatine transporter deficiency	*SLC6A8* (#300352)	Early onset dystonia (3 month of age) worsened by infections, oromandibular dystonia. Choreiform movements (33 months of age) appear later	Failure to thrive, microcephaly, ID, hypotonia, spasticity, S, behavioral abnormalities	↑urinary and plasma creatine, ↑urinary creatine-to-creatinine ratio	BA, DM, TCC, ↓creatine peak on MRS	Creatine, arginine and glycine supplementation	(43)
Tyrosine hydroxylase deficiency	*TH* (#605407)	There have been described three phenotypes: (1) TH-deficient dopa-responsive dystonia (the mild form of TH deficiency) age onset: 12 months to 12 years of age, initial symptoms are typically lower-limb dystonia and/or difficulty in walking (2) TH-deficient infantile HRS (hypokinesia, rigidity of extremities, and/or tremor) with motor delay (the severe form), age at onset: 3 to 12 months, and (3) TH-deficient progressive infantile encephalopathy (the very severe form), age at onset: 3 to 6 months: severe hypokinesia and limb hypertonia	Oculogyric crisis, ptosis, truncal hypotonia	↓CSF HVA, ↓CSF MHPG, normal CSF 5-HIAA	Normal, TH-deficient progressive infantile encephalopathy present mild brain or cerebellar atrophy or periventricular WM changes	L-dopa	(44)
Aromatic L-amino acid decarboxylase deficiency	*DDC* (#608643)	MD most often described are oculogyric crises (77%), dystonia (53%), and hypokinesia (34%). Dyskinesia (e.g. hyperkinesia, chorea, athetosis), myoclonus and tremor have also been described. Status dystonicus can occur in AADCD patients	Oculogyric crisis, ptosis, nasal congestion, hypotension, poor feeding, gastroesophageal reflux disease, ID, truncal hypotonia, hyperreflexia, sleep disturbances, paroxysmal sweating, temperature instability	↓CSF HVA, ↓CSF 5-HIAA, ↓ whole blood serotonin, ↑CSF, plasma and urinary L-Dopa, 5HTP, 3-OMD and 3-methoxytyrosine	Mild brain atrophy	Dopamine agonists, MAO inhibitors, pyridoxine, AAV-delivered gene therapy targeted to the SN	(45)
Dopamine transporter deficiency	*SLC6A3* (#613135)	Classical DTDS: nonspecific findings following by hyperkinetic MD (with features of chorea, dystonia, ballismus, orolingual dyskinesia). Atypical DTDS: Normal psychomotor development in infancy and early childhood is followed by later onset manifestations of parkinsonism-dystonia with tremor, progressive bradykinesia, variable tone, and dystonic posturing	Oculogyric crisis, ocular flutter, gastroesophageal reflux disease, poor feeding, truncal hypotonia	↑CSF HVA, normal CSF 5-HIAA	Normal	Dopamine agonists	(46)
Dopamine-serotonin vesicular transport defect	*SLC18A2* (#618049)	Gait dystonia, HRS, and oculogyric crises have been described	Oculogyric crisis, ptosis, nasal congestion, increased sweating, poor distal perfusion (cold hands and feet), truncal hypotonia, ID, hyperreflexia, hypernasal speech, temperature instability	↑urinary HVA and 5-HIAA, ↓urinary norepinephrine and dopamine, normal CSF neurotransmitters	Normal	Dopamine agonists	(47)
Autosomal recessive and autosomal dominant GTP cyclohydrolase I deficiency	*GCH1* (#233910) *GCH1* (#128230)	Childhood-onset dystonia and a dramatic and sustained response to low doses of oral administration of L-dopa. This disorder typically presents with gait disturbance caused by foot dystonia, later development of HRS signs. Occasionally, initial symptoms are arm dystonia, postural tremor of the hand, or slowness of movements. In general, gradual progression to generalized dystonia is observed	Abnormal ocular movement, ID, truncal hypotonia, S, episodic hyperthermia, torticollis, pes cavus	↑plasma phenylalanine, ↓CSF HVA and normal or ↓ 5-HIAA and ↓CSF biopterin and neopterin	Normal	Low Phe/BH4 supplementation, L-dopa, 5-hydroxytryptophan	(48)
Sepiapterin reductase deficiency	*SPR* (#612716)	Dystonia and oculogyric crisis are present >65% of patients. Other common features include HRS signs (tremor, bradykinesia, masked facies, rigidity), choreoathetosis and ataxia	Oculogyric crisis, oculomotor apraxia, microcephaly, ID, S, autonomic signs, sleep disturbances	↓CSF 5-HIAA, ↓CSF HVA, ↑CSF biopterin, ↑CSF dihydrobiopterin, ↑CSF Sepiapterin, ↓urinary HVA, 5-HIAA and VMA	Normal, rarely BA, DM	L-dopa, 5-hydroxytryptophan, BH4	(49)
DNAJC12-deficient hyperphenylalaninemia	*DNAJC12* (#617384)	Progressive MD with prominent dystonia	Nystagmus, oculogyric crisis, hypotonia, ID, autism	↑serum phenylalanine, ↓CSF dopamine and serotonin metabolites	—	BH4, L-dopa and/or 5- hydroxytryptophan	(50)
2-Methyl-3-hydroxybutyryl-CoA dehydrogenase deficiency	*HSD17B10* (#300438)	Generalized rigidity with some dystonic posturing at 15 years of age. Tremor can appear later in the disease course	SNHL, optic atrophy, retinal degeneration, nystagmus, hypertrophic cardiomyopathy, ID, hypotonia, S, spasticity	↑blood lactate, metabolic acidosis, ↑urinary 2-methyl-3 hydroxybutyrate, ↑urinary tiglylglycine	—	None	(51)
Glutaric aciduria type 1	*GCDH* (# 231670)	Progressive complex MD including dystonia and choreoathetosis. Dystonia is a significant sequela for individuals with BG injury. Those who have insidious onset generally have less severe MD	Failure to thrive, macrocephaly, hepatomegaly, S	Glutaricaciduria, metabolic acidosis, ketonemia, ketonuria, hypoglycemia	BG hyperintensities, frontotemporal atrophy, DM, dilatation of lateral ventricles	Carnitine, lysine-restricted/arginine- rich diet	(52)
**DISORDERS OF VITAMINS, COFACTORS, METALS AND MINERALS**							
Folate receptor alpha deficiency	*FOLR1* (# 613068)	Intention tremor, progressive ataxia and epileptic myoclonus are typically found at diagnosis (median onset 2 years, range 6 mo. to 4,5 y). Later, patients develop choreoathetosis	ID, hypotonia, congenital microcephaly, PN, S, sensory stimulus-sensitive drop attacks	↓CSF methyltetrahydrofolate	BG calcification, DM, BA	Folinic acid	(53)
Biotin-thiamine-responsive basal ganglia disease	*SLC19A3* (#607483)	Dystonia was the second most common sign after encephalopathy. The BFMDS questionnaire was administered to 34 SLC19A3 patients with dystonia (9.8 ± 1.6 points [mean 6 SEM]; range, 0–30). Higher BFMDS scores were identified in patients who had a previous history of developmental delay and in patients with disease onset before 6 months of age. A positive, and almost significant, correlation was observed between the BFMDS scores and the time from disease onset to thiamine initiation	Nystagmus, ophthalmoplegia, ptosis, episodic encephalopathy, S, truncal hypotonia, spasticity	Metabolic and lactic acidosis, ↓CSF free thiamine	BG hyperintensities	Thiamine, biotin	(54)
Pantothenate kinase-associated neurodegeneration (PKAN)	*PKAN2* (#234200)	Choreoathetosis, dystonia, including severe jaw-opening dystonia, HRS. Dystonia is always present and usually an early manifestation. Cranial dystonia and limb dystonia are frequent and may lead, respectively, to recurrent trauma to the tongue and to atraumatic long bone fracture from the combination of extreme bone stress and osteopenia. The resulting pain and distress can contribute to development of status dystonicus in a cycle that can be difficult to break	Pigmentary retinopathy, optic atrophy, ID, spasticity, psychiatric abnormalities	None	Eye of the tiger sign, BA	None	(14)
Alpha-tocopherol transfer protein deficiency	*TTPA* (#277460)	Late childhood or early teens between ages five and 15 years (age range: 2 to 37 years of age) progressive ataxia and clumsiness of the hand. Head tremor in 40% of cases	Areflexia, proprioception loss, xanthelasmata, tendon xanthomas	↓blood vitamin E, ↑serum cholesterol, triglycerides and beta-lipoprotein	Normal, CeA	Vitamin E	(55)
Wilson disease	*ATP7B* (#277900)	Neurologic involvement follows two general patterns: movement disorders or rigid dystonia. Movement disorders tend to occur earlier and include tremors, poor coordination, loss of fine-motor control, micrographia (abnormally small, cramped handwriting), chorea, and/or choreoathetosis. Spastic dystonia disorders manifest as mask-like facies, rigidity, and gait disturbance	Kayser-Fleischer ring, hepatomegaly, cirrhosis, renal tubular dysfunction, osteoporosis, chondrocalcinosis, PN	Hemolytic anemia, hypoparathyroidism, ↓serum ceruloplasmin, ↑urinary copper, proteinuria, aminoaciduria, glucosuria, hypercalciuria, hyperphosphaturia	Face of the Panda sign	Zinc, penicillamine, trientine	(10)
SLC30A10 deficiency	*SLC30A10* (#613280)	Although most cases show pure four-limb dystonia leading to a characteristic high stepping gait (a “cock-walk” gait) and fine motor impairment sometimes accompanied by dysarthria, fine tremor, and bradykinesia, one affected individual has pure spastic paraparesis without extrapyramidal dysfunction	Hepatomegaly, cirrhosis, PN	Polycythemia, ↑blood manganese, ↑unconjugated bilirubin, ↑transaminases, ↑erythropoietin, ↓iron, ↓ferritin, ↑TIBC	BG T1W hyperintensities	Chelation (EDTA), iron supplementation	(56)
SLC39A14 deficiency	*SLC39A14* (#617013)	Affected children presented with loss of developmental milestones, progressive dystonia and bulbar dysfunction in infancy or early childhood. Toward the end of the first decade, patients develop severe generalized pharmacoresistant dystonia, spasticity, limb contractures and scoliosis, and lost independent ambulation. Some showed parkinsonian features of hypomimia, tremor and bradykinesia	Microcephaly, scoliosis, ID, spasticity, hyperreflexia, ankle clonus,	↑blood manganese	BG T1W hyperintensities, BA, CeA	Chelation (EDTA)	(16)
**DISORDERS OF CARBOHYDRATES**							
Glucose transporter 1 deficiency	*SLC2A1* (*138140)	A complex MD is commonly seen and is characterized by ataxia, dystonia, and chorea that may be continuous, paroxysmal, or continual with fluctuations determined by environmental stressors. Often, paroxysmal worsening occurs before meals, during fasting, or with infectious stress.Clinical findings included the following: Gait disturbance (89%), the most frequent being ataxia and spasticity together or ataxia alone, action limb dystonia (86%), mild chorea (75%), cerebellar action tremor (70%), non-epileptic paroxysmal events (28%), dyspraxia (21%) and myoclonus (16%)Paroxysmal movement disorders. Paroxysmal exercise-induced dyskinesia and paroxysmal choreoathetosis are now recognized to be part of the phenotypic spectrum of Glut1 DS.Other associated findings included progressive spastic paraparesis with onset in early adulthood, mild gait ataxia, mild-to-moderate cognitive impairment, and epileptic seizures.It is unclear whether these events represent epileptic or non-epileptic phenomena	Microcephaly, S, hyperreflexia, ID	↓CSF glucose, ↓CSF lactate	Normal	Ketogenic diet, triheptanoin	(13)
Pyruvate carboxylase deficiency	*PC* (# 266150)	Ataxia, dystonia, tremor and HRS have been described	Hepatomegaly, proximal renal tubular acidosis, ID, hypotonia, S, ankle clonus	↑blood lactate, pyruvate and alanine, hypoglycemia, ↑serum ammonia, citrulline and lysine, ↑lactate: pyruvate ratio	BA, periventricular cysts and leukomalacia, DM, subcortical leukodystrophy	None	(57)
**MITOCHONDRIAL DISORDERS OF ENERGY METABOLISM**							
Pyruvate dehydrogenase complex deficiency	Various genes	Isolated paroxysmal exercise induced dystonia and intermittent isolated ataxia have been described. Late onset (mid-thirties) atypical parkinsonism, choreiform movements, stereotypies and ataxia have also been reported	Low birth weight, microcephaly, episodic ptosis, hypotonia, ID, S, facial dysmorphism (less frequent)	↑blood and CSF lactate and pyruvate, ↑blood alanine, ammonia	BG T2W hyperintensities, BA, agenesis of corpus callosum, ↑lactate on MRS	Thiamine, ketogenic diet	(58–61)
Succinyl-CoA ligase β subunit (SUCLA2) deficiency	*SUCLA2* (#612073)	Early-onset dystonia/hyperkinesia-deafness syndrome. Dystonia (85% of patients)	Failure to thrive, SNHL, ophthalmoplegia, ptosis, strabismus, hypotonia, ID, spasticity, hyporeflexia, S, PN	↑blood and CSF lactate, ↑CK, methylmalonic aciduria, methylglutaconic aciduria, intermittent aminoaciduria	BG T2W hyperintensities, BA	None	(39)
Succinyl-CoA ligase α subunit (SUCLG1) deficiency	*SUCGL1* (# 245400)	Dystonia (40% of patients)	Failure to thrive, SNHL, hypotonia, ID	↑blood and CSF lactate, hypoglycemia, methylmalonic aciduria, abnormal mitochondrial RCC activities	BG T2W hyperintensities, BA	None	(62)
L-2-hydroxyglutaric aciduria	*L2HGDH* (# 236792)	Dystonia, ataxia and intention tremor have been reported	SNHL, optic atrophy, strabismus, nystagmus, ID, S	↑serum lysine, ↑ serum, urinary and CSF L-2-hydroxyglutaric acid	Leukoencephalopathy with cavitation, BA, CeA	None	(27, 63)
Leigh Syndrome	>75 genes	Dystonia is a common feature in Leigh syndrome. Choreic movements have been found in Leigh syndrome, especially in children with ATPase 6 point mutations. Myoclonus has also been reported in some patients	Failure to thrive, ophthalmoplegia, optic atrophy, nystagmus, strabismus, ptosis, pigmentary retinopathy hypotonia, hypertrichosis, ID, spasticity, hyperreflexia, S	↑blood and CSF lactate	BG, cerebellum and brainstem T2W hyperintensities	None	(22)
Myoclonic epilepsy with ragged red fibers (MERRF)	Various genes	Ataxia and myoclonus	S, spasticity, SNHL	↑blood lactate, ↑pyruvate	CeA, atrophy of superior cerebellar peduncles	None	(64)
Neuropathy, ataxia and retinitis pigmentosa (NARP)	MTATP6 (*516060)	Proximal neurogenic muscle weakness with sensory neuropathy, ataxia, and pigmentary retinopathy. Symptoms usually start in childhood	Retinitis pigmentosa, nystagmus, ID, S, PN	None	Normal, BA, CeA	None	(65)
MEGDEL Syndrome	*SERAC1* (**#** 614739)	Starting at a median age of 6 months, muscular hypotonia (91%) was seen, followed by progressive spasticity (82%, median onset 5 15 months) and dystonia (82%, 18 months). The majority of affected individuals never learned to walk (68%)	Failure to thrive, microcephaly, SNHL, optic atrophy, neonatal hepatic dysfunction, hypotonia, ID, spasticity, S, recurrent infections, neonatal sepsis	↑blood lactate, ↑transaminases, ↑AFP, hypoglycemia, coagulopathy, 3-methylglutaconic aciduria, ↓cholesterol	BG T2W hyperintensities sparing central putamen, BA, CeA	None	(39)
DNAJC19 deficiency	*DNAJC19* (#610198)	Non-progressive cerebellar ataxia	Prenatal growth failure, optic atrophy, dilated cardiomyopathy, long QT syndrome, steatosis, hypospadias, cryptorchidism, ID	Microcytic anemia, ↑transaminases,3-methylglutaconic aciduria, 3-methylglutaric aciduria	BG T2W hyperintensities	None	(66)
Mohr-Tranebjaerg syndrome	*TIMM8A* (**#** 304700)	Deafness-dystonia-optic neuronopathy (DDON) syndrome is a progressive disorder. Dystonia and ataxia may appear in adolescent. One case of rapidly progressive dystonia who died at 16 years of age has been described	SNHL, cortical blindness, fractures, spasticity, hyperreflexia, mental regression, behavioral abnormalities	None	BG atrophy in males older than 40 years of age	None	(17)
CLPB deficiency	*CLPB* (#616271)	Severe cases present with hyperekplexia or absence of voluntary movements in the neonatal period. Moderate cases show progressive MD (ataxia, dystonia, and/or dyskinesia) of varying severity	Failure to thrive, microcephaly, cataracts, neonatal hypotonia, ID, spasticity, S, recurrent infections	Neutropenia, ↑urinary 2-methylglutaconic acid	BG atrophy, BA, CeA	None	(67)
Sacsin deficiency	*SACS* (#270550)	Mild to moderate early onset gait ataxia (age at onset 16-18 months of age) (33%), tremor (10%)	Nystagmus, pes cavus, PN, spasticity, hyperreflexia, ID (rare)	None	Vermis atrophy	None	(68)
**DISORDERS OF LIPIDS**							
Mitochondrial enoyl-coA reductase deficiency	*MERC* (#617282)	Childhood-onset progressive dystonia, facial chorea, dyskinesias and myoclonus: ages 1-6.5 years	Optic atrophy, nystagmus, spasticity, hyperreflexia	↑CSF lactate, abnormal mitochondrial RCC activities	BG hyperintensities	None	(69)
ELOVL4 deficiency	*ELOVL4* (#133190)	AD inheritance: adult onset (30-40 years of age) slowly progressive ataxia, pyramidal tract signs, and cerebellar and pontine atrophy detected on MRI, erythrokeratodermia may be present. AR inheritance: spastic paraplegia, ichthyosis and ID	Nystagmus, supranuclear gaze palsy, erythrokeratodermia, spasticity, PN	None	CeA, pontine atrophy, pontine midline linear hyperintensity	None	(70)
PLA2G6-associated neurodegeneration	*PLA2G6* (*603604)	PLAN encompasses a continuum of three overlapping phenotypes: 1) infantile onset PLAN, corresponding to classic infantile neuroaxonal dystrophy, 2) childhood-onset PLAN corresponding to atypical neuroaxonal dystrophy (ANAD) and 3) juvenile adult-onset PLAN corresponding to PLA2G6-related dystonia-parkinsonism. These patient exhibit predominantly HRS, resting tremor and limbs, oromandibular or generalized dystonia	Psychomotor regression, hypotonia, pyramidal tract signs, a/hyperreflexia, S, PN	None	CeA, BA, cerebellar cortex T2W hyperintensities, thin optic chiasm, BG iron deposition		(24)
Fatty acid hydroxylase-associated neurodegeneration	*FA2H* (#612319)	Variable phenotype NBIA, SPG35 (spastic paraparesis) and leukodystrophy (dystonia). Early childhood onset shows predominantly lower limb spastic tetraparesis and truncal instability, cerebellar ataxia, and cognitive deficits, often accompanied by movement disorders. The disease is rapidly progressive with loss of ambulation after a median of 7 years after disease onset	Optic atrophy, nystagmus, external ophthalmoplegia, spasticity, hyperreflexia, S	None	TCC, CeA, brainstem atrophy, WM abnormalities, iron deposition in globus pallidus	None	(15)
**STORAGE DISORDERS**							
Beta-propeller protein-associated neurodegeneration (BPAN)	*WDR45* (# 300894)	The affected individuals universally showed an early-onset global developmental delay that is static until adolescence/early adulthood when a secondary neurological decline is noted including HRS, dystonia and dementia	Eye movement abnormalities, retinal atrophy, ID, S, behavioral abnormalities	None	Globus pallidus T1W hyperintensities, BA, CeA	None	(25)
Sialidosis	NEU1 (*608272)	Slowly progressive ataxia and myoclonus (age at onset: 9 years of age)	Coarse facies, SNHL, nystagmus, cherry-red spot, lens opacity, cardiomyopathy, neonatal ascites, hepatomegaly, splenomegaly, inguinal hernia, dysostosis multiplex, muscle weakness, S, ID, hypotonia, hyperreflexia, hydrops fetalis	Vacuolated lymphocytes, proteinuria, ↑ urine sialyloligosaccharides and sialylglycopeptides	Normal	None	(71)
Galactosialidosis	CSTA (#256540)	Juvenile/adulthood phenotype (Japanese patients): myoclonus and ataxia	Coarse facies, SNHL, ID, S, dysostosis multiplex, corneal clouding, red cherry-spot	↑urine sialyloligosaccharides, normal sialic acid	Enlarged ventricles, hyperintense WM, striato thalamic vasculopathy, widened periencephalic spaces	None	(72)
Niemann-Pick disease type C	*NPC1* (#257220) *NPC2* (#607625)	Prominent MD: typically beginning as action dystonia in one limb and gradually spreading to involve all of the limbs and axial muscles. Speech gradually deteriorates, with a mixed dysarthria and dysphonia. Facial, orolingual and limbs severe dystonia, cerebellar truncal and limbs ataxia, gelastic cataplexy. Slowly progressive course. Age at onset: 10 to 14 years of age	Vertical supranuclear gaze palsy, hepatomegaly, splenomegaly, neonatal jaundice, fetal ascites, hypotonia, ID, spasticity, S, cataplexy, behavioral alterations	Enzyme analysis, Filipin test		None	(12, 40)
Action myoclonus-renal failure syndrome	*SCARB2* (**#** 254900)	Adolescent–young adulthood onset, progressive action myoclonus, ataxia and tremor, absence of mental deterioration	Nephrotic syndrome, renal failure, S	Thrombocytopenia, proteinuria	Thrombocytopenia, proteinuria	None	(73)
**DISORDERS OF PEROXISOMES**							
Sterol carrier protein-2 deficiency	*SCP2* (*184755)	Spasmodic torticollis and dystonic head tremor have been described at 7 years of age as well as slight cerebellar signs with intention tremor. Dystonic head tremor can be triggered by stressful situations	Abnormal eye movements, azoospermia	Hypergonadotropic hypogonadism	WM hyperintensities, butterfly lesions of the pons	None	(74)
**CONGENITAL DISORDERS OF GLYCOSYLATION**							
Phosphomannomutase 2 deficiency	*PMM2* (#212065)	Prominent cerebellar ataxia with generalized muscular hypotonia, generalized or segmental/multifocal dystonia, choreoathetosis. Stereotypies and tremor have also been described	Nonimmune hydrops fetalis, ID, hypotonia, S, hyporeflexia, stroke-like episodes, PN, abnormal subcutaneous fat tissue distribution, inverted nipples, joint contractures, hepatomegaly, nephrotic syndrome, cardiomyopathy, retinitis pigmentosa, nystagmus, microcephaly, failure to thrive	↑transaminases, thrombocytosis, ↓IgA, ↓IgG, ↓Cu, ↓Fe, ↓Zn, ↓cholesterol, ↓albumin, ↓Factor XI, ↓antithrombin III, abnormal TIFT (type 1 pattern), proteinuria, prolonged prothrombin time, hypergonadotropic hypogonadism, hypothyroidism	Olivopontocerebellar hypoplasia, CeA	None	(41)
ST3GAL5-CDG	*ST3GAL5* (*604402)	Choreoathetoid and dystonic cerebral palsy, startle myoclonus onset between 2 weeks and 3 months before GTCS seizures	Failure to thrive, microcephaly, deafness, optic atrophy, ID, S, hypotonia	None	Diffuse BA	None	(75, 76)
Steroid 5 alpha-reductase 3 deficiency	*SRD5A3* (*611715)	Early onset cerebellar ataxia (onset before 3,5 years of age)	ID, hypotonia	↑transaminases, ↓IGF1, ↓IGFBP3, microcytic anemia, coagulation defects, antithrombin III deficiency, abnormal TIFT (type 1 pattern)	Polymicrogyria or cerebellar vermis hypoplasia	None	(77)
GOSR2-CDG	*GORS2* (#614018)	Early onset ataxia (on average at 2 years of age), action myoclonus and myoclonic seizures (onset average at 6,5 years), rest tremor. Transient episodes of motor deterioration triggered by infection and fever	Mild ID, S, scoliosis	↑CK	Normal	None	(78)
N-glycanase 1 deficiency	*NGLY1* (*610661)	All affected individuals exhibited choreoathetosis, dystonia, myoclonus and action tremor. These movements were more severe in younger individuals	ID, PN, S, hypotonia, microcephaly	↑transaminases, ↑AFP, ↑blood lactate, abnormal urine oligosaccharides, normal TIFT, normal N-glycan analysis	Normal to BA, DM, or prominent Virchow Robin spaces	None	(79)

This table includes 47 out of the 231 IEMs identified in which MDs are usually a main symptom, either due to the fact of the frequency or the severity in which they appear. Treatable IEMs are included in this table.

*A, ataxia; AAV, adeno-associated virus; AFP, alpha-fetoprotein; BA, brain atrophy; BG, basal ganglia; CA, choreoathetosis; CeA, Cerebellar atrophy CK, creatine kinase; D, dystonia; DM, delayed myelination; 5-HIAA, 5-hydroxyindoleacetic acid; HRS, hypokinetic rigid syndrome; 5HTP, 5-hydroxytryptophan; HVA, homovanillic acid; ID, intellectual disability; M, myoclonus; MHPG, 3-methoxy-4-hydroxyphenylethyleneglycol; 3-OMD, 3-ortho-methyldopa; PN, peripheral neuropathy; RCC, respiratory chain complex S, seizures; SNHL, sensorineural hearing loss; T, tremor; TCC, thin corpus callosum; TIBC, total iron binding capacity; TIFT, transferrin isoelectric focusing test; VMA, vanillyl mandelic acid; WM, white matter*.

IEMs should repeatedly be considered, even in patients in whom an acquired cause of MD is assumed (e.g., infantile cerebral palsy). In a review by Leach et al. they identified 67 treatable IEMs that mimicked infantile cerebral palsy. Seventy-four percent of these IEMs have a specific treatment or a treatment that allows stabilizing/preventative effects. Fifty-seven percent of these IEMs are detected with straightforward metabolic/biochemical tests (80).

With regards to previous diagnostic algorithms of IEMs and MD, a simple scheme for adult patients was proposed by Sedel et al. that demonstrated a diagnostic approach based first on the clinical course of symptoms and the brain MRI (4). This very simple scheme allows the exclusion of the most frequent etiologies speedily whilst it cannot be applied altogether to childhood-onset MD because the diseases differ from adults.

One as well the other, IEM and MD impact intensively children's quality of life (QOL). In the study by Eggink et al., it was found that increasing severity and lower adaptative abilities of MD positively correlated with lower (QOL) scores (2). For this reason, adequate treatment can better ameliorate the quality of life of these children.

## Approach to Child With Suspected IEM and MD

**Figure 2:**
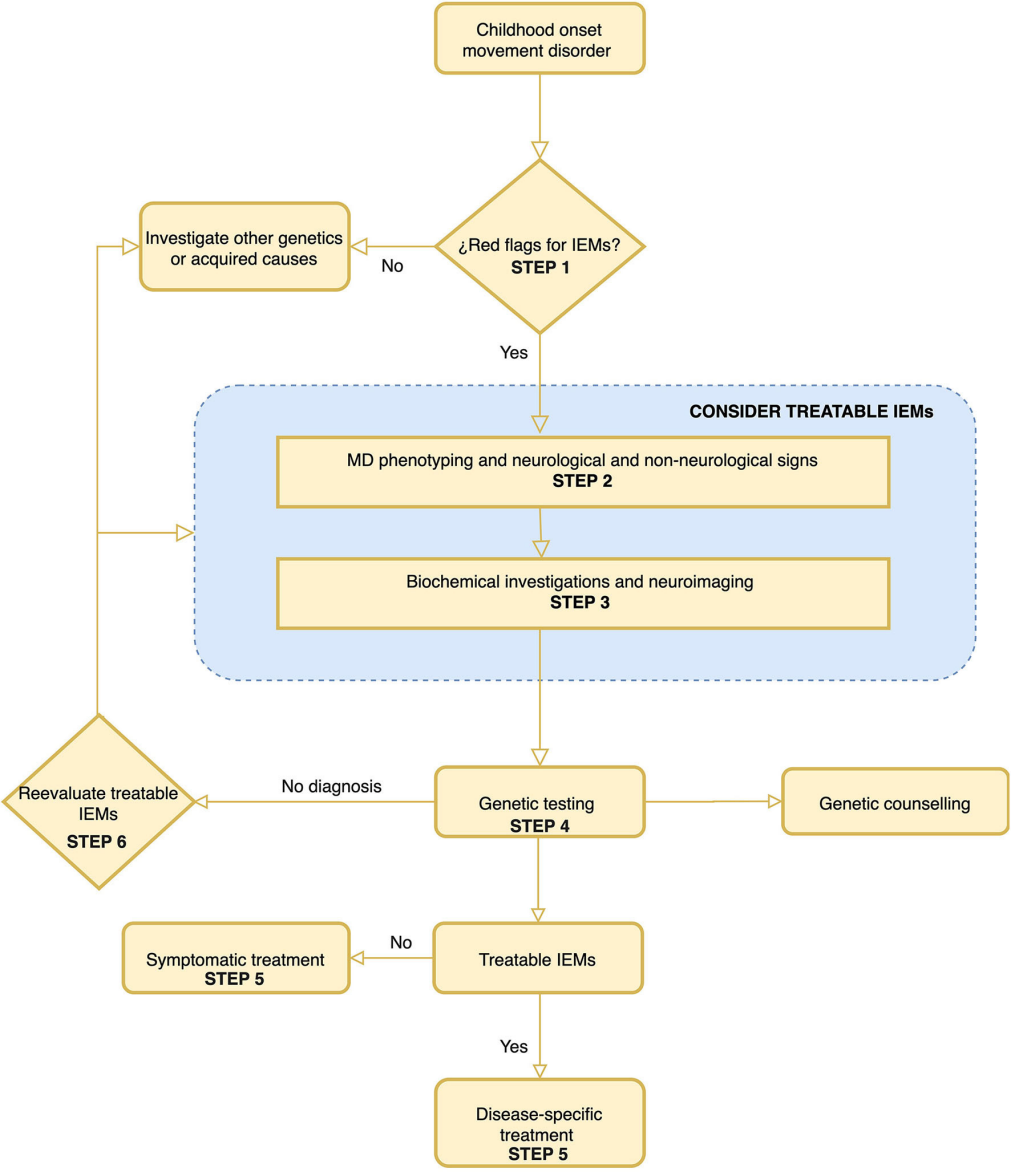
A proposed algorithm for the diagnosis of inborn error of metabolism in childhood-onset movement disorders. The most important steps of this algorithm are step 1 “Red Flags for IEMs” and step 6 “Rethink of treatable IEMs.” It is important to highlight that treatable IEMs should always be taken into consideration. The rationalization of the different steps is developed in the text of the article.

In this work, a six-step diagnostic algorithm was drafted and represented in Figure 2. The algorithm is based on simple steps that are listed below. The algorithm helps decide whether to consider a metabolic cause in a patient with MD. It also guides in the characterization of the MD, considering the characteristics that will most help guide a defined IEMs. It presents a list of neurological and non-neurological symptoms to take into account to establish the differential diagnosis of IEMs. Dependent on each MD, it proposes a list of minimal biochemical studies and also a list of IEMs to consider in fulfillment of the neuroradiological findings. Finally, a summary of the available treatments of both the IEMs and the MD is made. Once more, it is very important to note that the algorithm encourages thinking about treatable causes across different steps of the process.

## Step 1. When to Suspect an IEM in MD?

IEMs should be included in the differential diagnosis of all children with MD, even though there are several signs and symptoms that should indicate that a particular MD may be produced by an IEM. Some of these signs and symptoms are (3, 4, 81):

1) Diffuse clinical picture with several neurological and non-neurological signs,
2) Combination of different MD (ataxia plus dystonia, dystonia plus parkinsonism, chorea plus dystonia and myoclonus with any other MD or neurological or non-neurological signs, other than seizures),
3) Acute or subacute onset, remarkably if the onset is associated with encephalopathy/coma or if onset is precipitated by a concurrent febrile illness, starvation, physical exhaustion, or after a high-protein ingestion,
4) Insidious onset in a patient with multiple previous non-systemic manifestations,
5) Consanguinity and/or recessive or X-linked inheritance pattern,
6) Distinct neuroradiological findings for instance basal ganglia abnormalities, white matter involvement (hypomyelination/leukoencephalopathy) or cerebellar atrophy,
7) Atypical or progressively abnormal MD that fails to respond to standard treatment, and
8) MD that are not explained by classical etiologies (e.g., structural brain lesion, infectious/parainfectious or autoimmune disorders, toxic or drug induced MD and other well-known genetic or neurodegenerative MD, etc.).

## Step 2. Characterize the MD Phenotype and Other Neurological and Non-neurological Signs

### Neurological Signs

#### Movement Disorder

##### Phenomenology of MD

Not surprisingly this is the most significant step in the algorithm and also the one that can offer the most difficulty. The characterization of the MD must be in line with the definitions of Sanger et al., and reinforce in the own experience of the physician (6). As far as possible it is of the utmost importance to have also the assessment of an expert in pediatric MD. In a straightforward manner, it should be determined:

1. If the child presents unwanted excess movement (hyperkinetic movements) will be classified taking into account the definitions for dystonia, chorea, myoclonus, tremor, and stereotypies fairly detailed by Sanger et al. (6). The definition of ataxia is not included in the latter; accordingly, this definition will be used:
  - Ataxia: consists of the decomposition of movement due to breakdown of normal coordinated execution of a voluntary movement. Dysmetria, dysdiadochokinesia, intentional tremor, hypotonia, wide-based gait, or inability to tandem walk are frequently associated signs of ataxia. Early onset cerebellar ataxia consists in a large group of patients with rare disorders, manifesting symptomatic cerebellar ataxia before the age of 25 years (82).
  It is worth noting that tics are not habitually associated with IEMs, hence have not been include in this article, despite the fact that few cases of patients with tics have been reported in the following IEMs: Lesch-Nyhan disease (83), juvenile neuronal ceroid-lipofuscinosis (84), pantothenate kinase-associated neurodegeneration (85), Wilson disease (86), POLG disease (87), mannosidosis (88), *COASY*-associated neurodegeneration (89), and Maple Syrup Urine Disease (90).
2. If the child presents decreased (hypokinesia) and slow (bradykinesia) movements, rigidity or rest tremor. These are all signs of parkinsonism or hypokinetic rigid syndrome. These signs commonly do not appear all at the same time in a child, therefore the syndrome is routinely “incomplete” (7). The diagnosis of HRS greatly narrow possible IEMs. In this instance, is extremely helpful to observe the recommendations made by Garcia-Cazorla et al. to separate etiologies on the basis of age at onset (7). HRS starting at very young age (<2 years) can guide us to the diagnosis of monoamine neurotransmitter defects, mitochondrial diseases or Neurodegeneration with Brain Iron Accumulation disorders. On the contrary, HRS starting in children older than 2 year of age, can guide us in addition to those formerly mentioned in the following disorders: Wilson disease (10), GLUT-1 deficiency (13), Niemann-Pick type C (12), Gaucher disease (27), manganese accumulation disorders (16, 56), glutaric aciduria type 1 (52), Celia's encephalopathy (91), gangliosidosis (26), and cerebrotendinous xanthomatosis (9). Although it should be noted that HRS generally appears predominantly in the initial stages of the monoamine neurotransmitter defects or mitochondrial disorders, emerging later in the remaining IEMs.

By virtue of the response to treatment, it is important to highlight a very important group of treatable IEMs frequently presenting with HRS: the monoamine neurotransmitter disorders. The clinical phenotypes of these IEMs are predominantly neurological and the symptoms are similar to other childhood neurological disorders, such as cerebral palsy, hypoxic ischemic encephalopathy, or epileptic encephalopathies, as a result the monoamine neurotransmitter disorders are not recognized and are often misdiagnosed. Marked diurnal variation of motor symptoms (axial hypotonia and gait disturbances) and MD (dystonia, HRS, dyskinesia/chorea, tremor, and less commonly myoclonus) are often prominent along with autonomic dysfunction (sweating, hypersalivation, nasal congestion, and temperature dysregulation). Detailed MD phenotype, other clinical features as well as biochemical findings and neuroimaging of monoamine neurotransmitter disorders appears in Table 1. Prompt diagnosis is fundamental, considering that many respond to treatment and that adequate therapy is curative in some disorders. Conversely, the remarkable response to L-dopa of any childhood-onset undiagnosed MD should also guide us to this group of IEMs (8).

Commonly a child affected by an IEM present more than one type of MD. To follow the proposed six-step algorithm, we should take into account all the MD that we can identify regardless the fact that we will always take into more consideration: (1) the most prominent MD in the patient or (2) the least frequent (more specific) MD in all cases. In this sense, diseases that show myoclonus or HRS are less numerous, limiting for the best our diagnostic options. A list of treatable IEMs according to the MD they present appears in Supplementary Table 3.

##### MD Age at Onset and Type of Onset

In some cases, the MD begins earlier in the prenatal or the neonatal period. With respect to infants and children, there are plenty IEMs presenting with MD that can debut at these group of ages, consequently the isolated age of onset criteria will not be useful to differentiate between the different IEMs. Although, there are some IEMs presenting MD frequently before 2 years of age. Many MD in IEMs have an onset in adulthood regardless numerous cases with non-specific non-neurological symptoms as early as childhood or adolescence (Table 2).

**Table 2:** IEMs presenting with MD by age at onset.

**Prenatal**	**Neonatal**	**Infancy and childhood**	**Adolescence and adulthood**
Atypical Gaucher disease due to saposin C deficiency (92) Glycine encephalopathy (93, 94)	Hyperekplexia or hyperexcitability: SUOX - Isolated sulfite oxidase deficiency (95) Absence of voluntary movements or HRS: CLPB 3-methylglutaconic aciduria disorder (67), ADSL adenylosuccinate lyase deficiency (96) and PC pyruvate carboxylase deficiency (97) Myoclonic jerks: GLDC and AMT - glycine encephalopathy (93, 94) Tremor, jitteriness, dystonia: HTRA2 3-methylglutaconic aciduria type 8 (98)	Most of the IEMS presenting with MD begin in this age group Onset before 2 years of age: disorders of purine and creatine metabolism, (42, 43) neurotransmitters disorders, (44–49) propionic (99) and methylmalonic acidemia, (100) glutaric aciduria type 1, (52) disorders of cobalamin metabolism, (38) biotinidase deficiency, (101) manganese disorders *(SLC39A8*), (102) GLUT-1 deficiency, (13) mitochondrial disorders (including Leigh syndrome), (22) SNX14 deficiency, (20) CLN14 disease, (40) Niemann Pick type C, (12–40) Sialidosis, (71) PMM2-CDG (41) and other congenital disorders of glycosylation. (75–79)	Adult-onset ataxia: 3-methylglutaconyl-CoA hydratase deficiency, (103) 3-phosphoglycerate dehydrogenase deficiency (104), γ-glutamylcysteine synthetase deficiency, (105) OPA1 deficiency, (106) very long-chain fatty acid elongase 4 deficiency, (70) very long-chain fatty acid elongase 5 deficiency, (107) abetalipoproteinemia, (108) hereditary coproporphyria, (109) and complex MD (tremor, ataxia, myoclonus, perioral dyskinesias) cathepsin F deficiency, (110) (myoclonus, cerebellar ataxia, parkinsonism) Neuronal ceroid lipofuscinosis type 4 (Parry type) (23)

*To be noted that many IEMs presenting with MD do not have a specific age of onset, hence usually this parameter may not be extremely valuable in evaluating a particular case*.

MD that appear acutely, associated or not with encephalopathy, are generally caused by diseases that are considered medical emergencies. In cases where the onset of MD coincides with a metabolic decompensation, there is several times an acute injury to the basal ganglia or the cerebral cortex, in most cases with accompanying encephalopathy/coma, as is often the case in Leigh syndrome (22), glutaric aciduria type 1 (52), propionic acidemia (99), and other organic acidurias or mitochondrial disorders. Injury to the basal ganglia can also occur with the appearance of MD delayed as in the β-ketothiolase deficiency (111). In the remaining IEMs, there is very often no clear onset of MD disorder.

In a large number of these diseases there is an identifiable trigger as for example: fever [ataxia: Maple Syrup Urine Disease (112), episodic ataxia type 6 (113), milder phenotypes of glycine encephalopathy (93, 94), and GORS2-CDG (78), dystonia: β-ketothiolase deficiency (111) and mitochondrial short-chain enoyl-CoA hydratase 1 deficiency (114)], fasting or fever [(complex MD: GLUT1 deficiency (13)], exercise [(ataxia or dystonia: mitochondrial short-chain enoyl-CoA hydratase 1 deficiency (114), pyruvate dehydrogenase complex deficiency (58–61)] and lead exposure [ataxia: hereditary coproporphyria (109)]. In other cases, the trigger is regularly more subtle as stressful situations and dystonic head tremor in sterol carrier protein-2 deficiency (74) or else minor febrile illnesses and brief episodes of ataxia in citrullinemia (115).

Notwithstanding, status dystonicus is a rare and life-threatening MD emergency. It has been described in the following IEMs: Wilson disease (116), Pantothenate kinase-associated neurodegeneration (117–120), aromatic l-amino acid decarboxylase (AADC) deficiency (45) and *SLC6A3* dopamine transporter deficiency syndrome (121). Management is adjusted to the characteristics and complications of each patient. The use of dystonia action plans can facilitate intervention or avoid progression to dystonic state. One of these plans have been describe in detail by Allen et al. (122). If the dystonic refractory state persists despite drug therapy, early consideration of intrathecal baclofen or deep brain stimulation is required.

Finally, involuntary MD can in addition appear during treatment, for example tremor with the administration of vitamin B12.

##### MD Distribution

Focal MDs in IEMs are rare and are generally associated with asymmetric radiologic injury. Multifocal or segmental MDs have been described in: dystonia: PMM2-CDG (41), myoclonus: Pyridoxamine 5′-phosphate oxidase deficiency (123) and hemiplegic episodes: episodic ataxia type 6 (113) and MELAS (124). In the remaining IEMs, the distribution of the MD is usually generalized. In this context, the MD distribution is not very useful to guide the diagnosis.

On the contrary, in certain patients, the distribution can be very useful, as in the case of oromandibular dystonia noted in just a few of IEMs: cerebral creatine deficiency (43), GCDH1 dopa-responsive dystonia (48), *PLA2G6*-associated neurodegeneration (24), *COASY*-associated neurodegeneration (125), *CP*-aceruloplasminemia (126), fucosidosis (127), hypermanganesemia due to *SLC39A14* (16), CLN3 (128), GM1 gangliosidosis (26), *TIMM8A*-Deafness-Dystonia-Optic Neuronopathy Syndrome (17), and biotin-thiamine responsive basal ganglia disease (54).

In a similar manner, dystonic head tremor observed in *AFG3L2*-spinocerebellar ataxia type 28 (129), *GBA2*-Autosomal recessive spastic paraplegia type 46 (130), *PEX6*-Peroxin 6 deficiency (131), *PEX16*- Peroxin 16 deficiency (132), Niemann Pick type C disease (12), *NAXE*-NAD(P)HX epimerase deficiency (133), *TTPA*-vitamin E deficiency (55), and *SCP2*-Sterol carrier protein-2 deficiency (74) gives an excellent clue for the diagnosis of the IEMs.

##### MD Temporal Pattern

In the majority of IEMs, as expected, MD presented persistently and continuously, due to this circumstances, the temporary pattern does not usually provide diagnostic benefits. In contrast, it is very useful in a restricted group of IEMs presenting with paroxysmal or intermittent MD (Table 3) as well as the monoamine neurotransmitter disorders whereby there is a very characteristic diurnal fluctuation of symptoms: motor symptoms, including MD, become more prominent in the evening and improve after sleep.

**Table 3:** IEMs presenting with paroxysmal MD.

**Ataxia**	**Dystonia**	**Chorea**	**Dyskinesia**
Pyruvate dehydrogenase complex deficiency (58–61) BTD-biotinidase deficiency (101) Hartnup disease (134) GLDC and AMT-glycine encephalopathy (93, 94) HTD-Tyrosinemia type III (135) SLC2A1-GLUT1 deficiency (13)	SLC2A1-GLUT1 deficiency (13) ECHS1-mitochondrial short-chain enoyl-CoA hydratase 1 deficiency (114) HIBCH-3-hydroxyisobutyryl-CoA hydrolase deficiency (136) Pyruvate dehydrogenase complex deficiency (58–61)	OTC- Ornithine transcarbamylase deficiency (137, 138)	ABAT-GABA transaminase deficiency (139) ALDH5A1-Succinic Semialdehyde Dehydrogenase Deficiency (140) PARK2-Parkin deficiency (141)

### Other Neurological Signs

In the second step, the neurological evaluation is completed with other signs and symptoms that can guide the diagnosis. The following signs and symptoms will be taken into account:

- Intellectual disability or developmental delay, cognitive regression, autistic features, self-injurious behavior
- Tone muscle and osteotendinous reflexes: hypotonia, pyramidal signs or spasticity and hypo/hyperreflexia
- Episodic encephalopathy/coma
- Seizures or epileptic encephalopathy
- Microcephaly/Macrocephaly
- Myopathy, contractures, polyneuropathy or pes cavus
- Optic atrophy, nystagmus, ptosis, oculogyric crises, oculomotor apraxia, ectopia lentis, glaucoma, strabismus, retinitis pigmentosa, retinal degeneration, chorioretinal dystrophy, cherry red-spot, supranuclear gaze palsy, ophthalmoplegia, and cataracts
- Sensorineural hearing loss (SNHL).

It is imperative to emphasize that the ophthalmological exploration can find numerous signs, which are ordinarily more or less specific and can exceedingly help guide the diagnosis of the child.

### Non-neurological Signs

Thus, far as possible these non-neurological signs and symptoms will be considered during the evaluation of a patient with IEMs:

- Dysmorphic or coarse facies
- Intrauterine growth retardation (IUGR), hidrops fetalis, postnatal failure to thrive, or short stature
- Vomiting, hepatomegaly, cirrhosis, splenomegaly, intestinal pseudo-obstruction, and pancreatitis
- Cardiomyopathy and long QT syndrome
- Frequent infections,
- Renal tubular acidosis, renal failure, nephrolithiasis, nephrotic syndrome, and glomerulosclerosis
- Other: gout, hair abnormalities (trichorrhexis nodosa), episodic hypothermia, skin (blonde hair, light-sensitive dermatitis, eczema, seborrheic dermatitis, alopecia, xanthelasmata, xanthomas, cutaneous leiomyomata, etc.), delayed teething, apneas, and skeletal abnormalities (scoliosis, osteoporosis, chondrocalcinosis, dysostosis multiplex etc.), etc.

The phenotype of the MD, as well as the neurological and non-neurological signs of each IEMs appears in the Supplementary Table 1. Some of the non-neurological symptoms are very specific to certain IEMS and may further assist in the diagnostic process.

## Step 3. Biochemical Investigation and Neuroimaging

Eighty-eight percent of the 231 IEMs with MD can be detected through biochemical tests in blood, urine, feces, fibroblasts or CSF. Nevertheless, the diagnosis in the remaining IEMs will only be accomplish with the genetic testing. The tests to request are heterogeneous and will vary in line with the assumed MD and the interest in excluding certain diseases. First-tier and second-tier testing have been considered for each MD in order to prioritize the diagnosis of treatable diseases (see Table 4). In the second-tier testing, more specialized or less common biochemical analysis (enzymatic activities, porphyrins, etc.) have been included. However, some tests of the second-tier should be considered at the beginning of the diagnostic process if the suspicion of a particular disease is very high. As expected, this list of biochemical tests is broader for patients with ataxia, the most frequent MD in patients with IEMs and the one with the most possible etiologies whereas the list become more restricted for patients with myoclonus and hypokinetic-rigid syndrome.

**Table 4:** Minimal biochemical testing to diagnose treatable IEMs based on the particular MD.

**Ataxia**	**Dystonia**
Blood: 1st Tier: hemoglobin, reticulocytes, blood count, ASAT/ALAT, glucose, uric acid, urea, creatine, guanidino compounds, ammonia, lactate, pyruvate, glutathione, amino acids, orotic acid, total homocysteine, acylcarnitines, methylmalonic acid, vitamin B12, folate, thiamine pyrophosphate, vitamin E, copper, ceruloplasmin, ferritin, manganese, VLCFA, sialotransferrins, CoQ10, acetoacetate, sterols. 2nd Tier: Galactose-1-P, GALT enzyme activity, TPP1 enzyme activity, beta-glucosidase enzyme analysis, arylsulfatase A enzyme analysis, alpha-mannosidase enzyme analysis, porphobilinogen, aminolevulinic acid, porphyrins, phytanic acid, pristanic acid, plasmalogens Urine: 1st Tier: uric acid, purines and pyrimidines, creatine, guanidino compounds, organic acids, thiosulfate, sulfites, copper, acetoacetate, sulfatide, oligosaccharides, pipecolic acid 2nd Tier: porphobilinogen, aminolevulinic acid, porphyrins, CSF: 1st Tier: neurotransmitters, amino acids, 5-Methyl-THF, free thiamine, pipecolic acid, glucose, lactate, protein, Fibroblasts: 2nd Tier: Cyclic NADHX Feces: 2nd Tier: porphyrins ^*^Celia's encephalopathy	Blood: 1st Tier: blood count, ASAT/ALAT, glucose, uric acid, creatine, guanidino compounds, prolactin, amino acids, pterins, total homocysteine, lactate, pyruvate, acylcarnitines, methylmalonic acid, thiamine pyrophosphate, pipecolic acid, AASA, copper, ceruloplasmin, ferritin, manganese, sialotransferrins, CoQ10, acetoacetate, sterols, VLCFA. 2nd Tier: Galactose-1-P, GALT enzyme activity, TPP1 enzyme activity, beta-glucosidase enzyme analysis, arylsulfatase A enzyme analysis, Urine: 1st Tier: uric acid, purines and pyrimidines, creatine, guanidino compounds, pterins, organic acids, thiosulfate, pipecolic acid, AASA, sulfites, copper, acetoacetate, sulfatide, CSF: 1st Tier: neurotransmitters, pterins, free thiamine, PLP, pipecolic acid, AASA, glucose, lactate, protein, ^*^Celia's encephalopathy
**Choreoathetosis**	**Tremor**
Blood: 1st Tier: blood count, ASAT/ALAT, glucose, lactate, pyruvate, uric acid, creatine, guanidino compounds, prolactin, amino acids, pterins, purine, total homocysteine, copper, ceruloplasmin, acylcarnitines, methylmalonic acid, folate, sterols, acetoacetate. 2nd Tier: Galactose-1-P, GALT enzyme activity, TPP1 enzyme activity Urine: 1st Tier: glucose, lactate, purine and pyrimidines, guanidino compounds, pterins, organic acids, sulfites, copper, acetoacetate CSF: 2nd Tier: amino acids, neurotransmitters, pterins, 5-Methyl-THF, pipecolic acid	Blood: 1st Tier: blood count, glucose, prolactin, ASAT/ALAT, copper, ceruloplasmin, ferritin, manganese, amino acids, acylcarnitines, AASA, pipecolic acid, CoQ10, acetoacetate, sterols. 2nd Tier: Galactose-1-P, GALT enzyme activity, beta-glucosidase enzyme analysis Urine: 1st Tier: copper, pterins, organic acids, AASA, pipecolic acid, acetoacetate CSF: 2nd Tier: glucose, lactate, neurotransmitters, pterins, 5-Methyl-THF, PLP, AASA, pipecolic acid ^*^Celia's encephalopathy
**Myoclonus**	**HRS**
Blood: 1st Tier: blood count, glucose, ASAT/ALAT, urea, prolactin, copper, ceruloplasmin, ammonia, amino acids, orotic acids, total homocysteine, AASA, pipecolic acid, CoQ10, sterols. 2nd Tier: beta-glucosidase enzyme analysis, TPP1 enzyme activity Urine: 1st Tier: copper, amino acids, orotic acid, pterins, AASA, pipecolic acid CSF: 2nd Tier: glucose, lactate, neurotransmitters, pterins, PLP, AASA, pipecolic acid ^*^Celia's encephalopathy	Blood: 1st Tier: Blood count, glucose, ASAT/ALAT, prolactin, copper, manganese, ceruloplasmin, ferritin, uric acid, total homocysteine, amino acids, acylcarnitines, sterols. 2nd Tier: Galactose-1-P, GALT enzyme activity, beta-glucosidase enzyme analysis Urine: 1st Tier: copper, purines, pterins, organic acids, sulfites CSF: 1st Tier: glucose, lactate, neurotransmitters, pterins, 5Methyl-THF, pipecolic acid

1st Tier and 2nd Tier biochemical testing have been suggested according to each MD. As expected, the minimal biochemical testing is more extensive in ataxia and dystonia, the most frequent MDs.

* *Celia's encephalopathy - BSCL2 deficiency requires genetic testing*.

Subsequent confirmation of a definite disease may be done through additional biochemical or genetic tests. In many countries where there is difficulty in performing biochemical tests, genetic testing should be done sooner rather than later. A vital aspect in the biochemical study of a patient is to know at all times the diseases that have been ruled out with the requested tests and, therefore, to make a more thought-out plan for each patient. An alternative and sometimes controversial approach is the early performance of the lumbar puncture in a patient with MD. Lumbar puncture is regularly included in the initial evaluation of all children with MD, first and foremost when there is no speedy availability of genetic tests. This approach is correct unless there is a differential diagnostic hypothesis in which a lumbar puncture is not considered necessary. Thus, analysis of cerebrospinal fluid in IEMs presenting with MD is essential and should be considered as a 1st Tier biochemical investigation, especially in cases of ataxia, dystonia, or HRS, taking into account the need for the precise diagnosis of certain IEMs (monoamine neurotransmitter disorders, GLUT-1 deficiency, etc.) with the consequent establishment of a specific treatment (8, 13).

In this section it is necessary to comment on newborn screening programs since many patients are diagnosed now even before presenting neurological or non-neurological symptoms. The main purpose of newborn screening programs is to diagnose genetic disorders early, allowing treatment to begin before symptoms appear. The quantification of amino acids and acylcarnitines in dried blood spots (DBS) by MS/MS allows the simultaneous detection of more than 30 metabolic disorders, including those associated with amino acid, organic acid, and fatty acid metabolism. Other disorders detected using different techniques, e.g., galactosemia, biotinidase deficiency, Pompe disease, and mucopolysaccharidosis type I, are now included in some NBS programs. In a study by Navarrete et al., diagnosis of an inborn error of metabolism were confirmed in fifty-nine percent of patients tested using a personalized exome sequencing panel (142).

Along with these baseline biochemical testing, neuroimage can identify patterns of treatable inborn errors of metabolism and helps to exclude other secondary causes of MD guiding further testing. Consequently, neuroimage should be considered part of the initial diagnostic evaluation (81). In chronic MD it is essential to make a correct assessment of the neuroimaging, which generally gives many clues to the specific diagnosis. In the IEMs that cause MD, a combination of cerebral atrophy, cerebellar atrophy, white matter alteration (hypomyelination, demyelination, nonspecific lesions, or decreased volume) and basal ganglia involvement (hyperintensity, metal deposit, etc.) can be observed. Additionally, less frequently injuries in other areas like the brain stem, corpus callosum or the spinal cord or metabolic can be detected.

**Figure 3:**
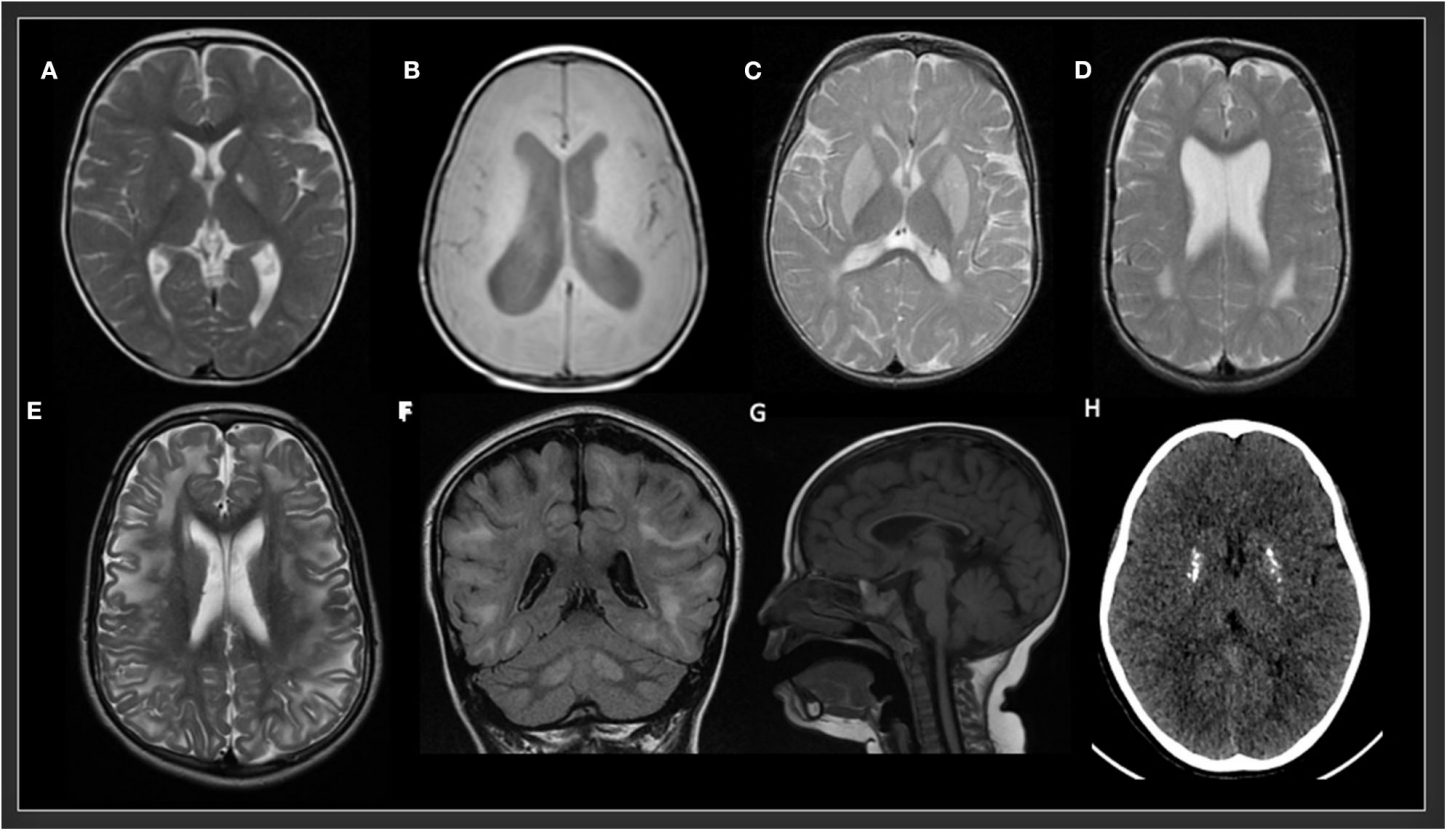
Radiological patterns in selected inborn errors of metabolism. **(A)** Mitochondrial short-chain enoyl-CoA hydratase 1 deficiency (*ECHS1*). MRI (T2W) showing bilateral symmetric signal hyperintensity in globus pallidus and a small cavitation in the left globus pallidus. **(B)** Type 3 Gaucher disease (*GBA*). MRI (T1W) showing decrease volume of white matter and hydrocephalus. **(C)** Glutaric aciduria type 1 (*GCDH*). MRI (T2 FSE) showing bilateral symmetric signal hyperintensity in putamen and globus pallidus and posterior periventricular white matter abnormalities. **(D)** Rhizomelic chondrodysplasia punctata, type 1 (*PEX7*). MRI (T2) showing bilateral symmetric posterior periventricular white matter hyperintensity and ventriculomegaly. **(E,F)** L-2-hydroxyglutaric aciduria (*L2HGA*). MRI (T2 and FLAIR) showing bilateral diffuse cerebral white matter and dentate nuclei hyperintensities. **(G)** Methylmalonic aciduria and homocystinuria, cblC type (*MMACHC*). MRI (T1) showing a very thin corpus callosum. **(H)** Aicardi-Goutières syndrome 2 (*RNASEH2B*). CT showing multiple calcifications in basal ganglia.

**Figure 4:**
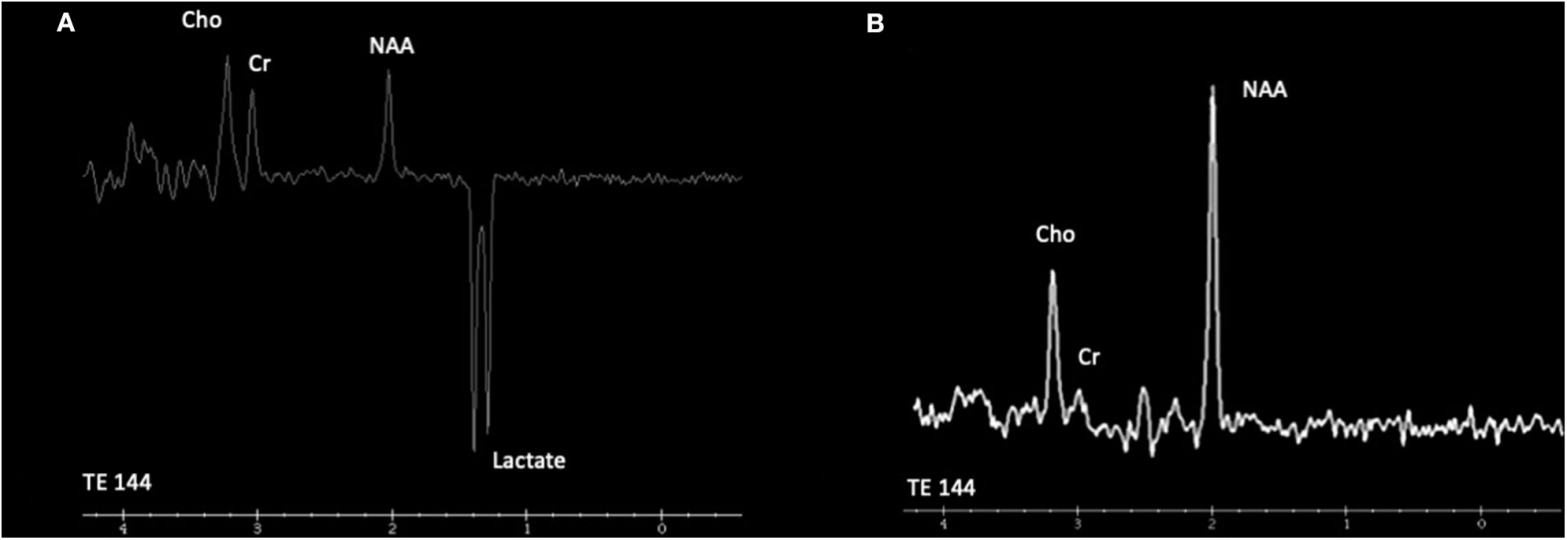
Cranial proton magnetic resonance spectroscopy (MRS). **(A)** Leigh syndrome (*NDUFS4*). Magnetic resonance spectroscopy using intermediate echo time (144 milliseconds) at the right caudate nuclei reveals the presence of an obvious inverted doublet of lactate at 1.33 ppm. **(B)** Cerebral creatine deficiency syndrome 1 (*SLC6A8*). Magnetic resonance spectroscopy using intermediate echo time (144 milliseconds) at the posterior periventricular white matter level reveals low creatine peak at 3.0 ppm. Cho, choline; Cr, creatine; NAA, N-acetyl aspartate.

In the study by Cordeiro et al., basal ganglia or gray matter abnormalities with or without white matter changes were present in sixty seven percent of the patients with IEMs (3). It is of the utmost importance to keep in mind that many IEMs can present normal neuroimaging in the initial stages and exclusively progress to cerebral or cerebellar atrophy in advanced stages of the disease. Therefore, it is necessary to be careful with the interpretation of a normal brain MRI. Spectroscopy can be useful in some cases: Creatine transporter deficiency (43), *SLC13A5*-Epileptic encephalopathy, early infantile (143), 25, Pyruvate dehydrogenase complex deficiency (58–61), thiamine pyrophosphokinase deficiency, biotin-thiamine-responsive basal ganglia disease, mitochondrial thiamine pyrophosphate transporter deficiency (54), and many other mitochondrial disorders (Figure 4). Detection of intracerebral or basal ganglia calcification is also suggestive of certain IEMs (Table 5, Figure 3): Dihydropteridine reductase deficiency (37), folate receptor alpha deficiency, dihydrofolate reductase deficiency, hereditary folate malabsorption (144), phenylketonuria (144), Krabbe disease (145) and Aicardi-Goutières syndrome (146). Figure 3 and Figure 4 present the radiological pattern and MR Spectroscopy of some IEMs and Table 5 shows a list of IEMs with disease-specific treatment classified according to the neuroradiological pattern.

**Table 5:** Radiological findings in IEMs with MD.

**Normal MRI**	**Brain**	**Cerebellum**	**Corpus callosum**	**White matter**	**Basal ganglia**	**Others**
Phosphoribosyl pyrophosphate synthetase 1 superactivity Isovaleric acidemia Glycine encephalopathy due to aminomethyltransferase deficiency Imerslund-Gräsbeck syndrome cblC disease Glucose transporter 1 deficiency Celia's encephalopathy Alpha-tocopherol transfer protein deficiency Coproporphyrinogen oxidase deficiency Holocarboxylase synthetase deficiency Tyrosine hydroxylase deficiency Aromatic L-amino acid decarboxylase deficiency Dopamine transporter deficiency Dopamine-serotonin vesicular transport defect Autosomal recessive GTP cyclohydrolase I deficiency Autosomal dominant GTPCH deficiency Sepiapterin reductase deficiency DNAJC12-deficient hyperphenylalaninemia Pyridoxine-dependent epilepsy PNPO deficiency COQ6 deficiency	BRAIN ATROPHY: Creatine transporter deficiency Imerslund-Gräsbeck syndrome Gaucher disease Cerebrotendinous xanthomatosis SLC39A14 deficiency Molybdenum cofactor deficiency Celia's encephalopathy Folate receptor alpha deficiency Pyruvate dehydrogenase complex deficiency Zellweger spectrum disorders Biotinidase deficiency Arginase deficiency Mitochondrial ornithine transporter deficiency Gaucher disease CLN2 disease NAXE deficiency Dihydrofolate reductase deficiency Tyrosine hydroxylase deficiency Aromatic L-amino acid decarboxylase deficiency Sepiapterin reductase deficiency DNAJC12-deficient hyperphenylalaninemia COQ6 deficiency SLC39A8 deficiency POLYMICROGYRIA, /PACHYGYRIA: Zellweger spectrum disorders Malonic aciduria HETEROTOPIAS: Zellweger spectrum disorders Argininosuccinate lyase deficiency Malonic aciduria INTRACEREBRAL CALCIFICATIONS: Dihydropteridine reductase deficiency Folate receptor alpha deficiency Dihydrofolate reductase deficiency Hereditary folate malabsorption Phenylketonuria	CEREBELLAR ATROPHY: CAD trifunctional protein deficiency Gaucher disease Cerebrotendinous xanthomatosis SLC39A14 deficiency Molybdenum cofactor deficiency (*GPHN*) COQ2 deficiency COQ8A deficiency Folate receptor alpha deficiency Alpha-tocopherol transfer protein deficiency dna: Cerebrotendinous xanthomatosis Thiamine pyrophosphokinase deficiency Biotinidase deficiency Argininosuccinate synthetase deficiency Argininosuccinate lyase deficiency Arginase deficiency Alpha-mannosidosis Dihydrofolate reductase deficiency Recessive porphobilinogen deaminase deficiency SLC39A8 deficiency CEREBELLAR WHITE MATTER ABNORMALITIES: Cerebrotendinous xanthomatosis CEREBELLAR POLYMYCROGIRIA: Molybdenum cofactor deficiency (*GPHN*)	THIN CORPUS CALLOSUM: Hypoxanthine guanine phosphoribosyltransferase deficiency Creatine transporter deficiency Gaucher disease Molybdenum cofactor deficiency Aromatic L-amino acid decarboxylase deficiency ABSENT/HYPOPLASIA CORPUS CALLOSUM: Glycine encephalopathy due to aminomethyltransferase deficiency Pyruvate dehydrogenase complex deficiency Zellweger spectrum disorders Pyridoxine-dependent epilepsy	DELAYED MYELINATION: Hypoxanthine guanine phosphoribosyltransferase deficiency Creatine transporter deficiency Methylmalonyl-CoA epimerase deficiency Glutaric aciduria type 1 Molybdenum cofactor deficiency X-linked adrenoleukodystrophy Zellweger spectrum disorders X-linked adrenoleukodystrophy Alpha-mannosidosis Dihydrofolate reductase deficiency Hereditary folate malabsorption Sepiapterin reductase deficiency 6-Pyruvoyl-tetrahydropterin synthase deficiency cblX disease LEUKODYSTROPHIES – WHITTE MATTER ABNORMALITIES: Maple syrup urine disease Phenylketonuria X-linked adrenoleukodystrophy Metachromatic leukodystrophy Methylenetetrahydrofolate reductase deficiency Argininosuccinate lyase deficiency X-linked adrenoleukodystrophy Alpha-mannosidosis Metachromatic leukodystrophy Folate receptor alpha deficiency NAXE deficiency Methylcobalamin synthesis defect - cblD variant 1 Tyrosine hydroxylase deficiency (periventricular) Aromatic L-amino acid decarboxylase deficiency Malonic aciduria Recessive porphobilinogen deaminase deficiency Classic galactosemia Methylmalonic aciduria due to methylmalonyl-CoA mutase deficiency Hypoxanthine guanine phosphoribosyltransferase deficiency HYPOMYELINATION: Folate receptor alpha deficiency Aromatic L-amino acid decarboxylase deficiency	T2W HYPERINTENSITIES: Guanidinoacetate methyltransferase deficiency Ornithine transcarbamylase deficiency Ethylmalonic encephalopathy Isovaleric acidemia Methylmalonyl-CoA epimerase deficiency Glutaric aciduria type 1 Beta-ketothiolase deficiency Pyruvate dehydrogenase complex deficiency Thiamine pyrophosphokinase deficiency Biotin-thiamine-responsive basal ganglia disease Mitochondrial thiamine pyrophosphate transporter deficiency Argininosuccinate synthetase deficiency Argininosuccinate lyase deficiency Malonic aciduria Wilson disease Methylmalonic aciduria due to methylmalonyl-CoA mutase deficiency T1W HYPERINTENSITIES: SLC39A14 deficiency SLC30A10 deficiency THALAMIC LESIONS: Glutathione synthetase deficiency Recessive porphobilinogen deaminase deficiency INFARCTION: Propionic acidemia IRON DEPOSITION: Aceruloplasminemia	CREATINE PEAK ON MRS: Creatine transporter deficiency ↑LACTATE PEAK ON MRS: Pyruvate dehydrogenase complex deficiency Thiamine pyrophosphokinase deficiency Biotin-thiamine-responsive basal ganglia disease Mitochondrial thiamine pyrophosphate transporter deficiency CORTICAL AND SUBCORTICAL EDEMA DURING DECOMPENSATION: Ornithine transcarbamylase deficiency Maple syrup urine disease Argininosuccinate synthetase deficiency STROKE: Classic homocystinuria Argininosuccinate lyase deficiency Methionine synthase deficiency – cblG Methylmalonic aciduria due to methylmalonyl-CoA mutase deficiency CEREBRAL PEDUNCLES ABNORMALITIES: Cerebrotendinous xanthomatosis INTERNAL CAPSULE/CORTICOSPINAL TRACT ABNORMALITIES: Cerebrotendinous xanthomatosis Refsum disease Glycine encephalopathy due to glycine decarboxylase deficiency FACE OF THE PANDA SIGN: Wilson disease Subependymal cysts: Zellweger spectrum disorders ABSENT OLFATORY LOBES: Zellweger spectrum disorders INTRACRANIAL HEMORRAGHE: Menkes disease SPINAL MYELOPATHY: NAXE deficiency Holocarboxylase synthetase deficiency cblC disease

*IEMs with likely normal neuroimaging have been separated from those with pathological neuroimaging. Six areas of assessment have been proposed: brain, cerebellum, corpus callosum, white matter, basal ganglia and other areas. Some neuroimage patterns can be highly suggestive of a group of IEMs, such as, basal ganglia T1W hyperintensities in manganese disorders*.

## Step 4. Genetic Investigation in IEMs

Genetic analysis is currently performed as a definitive test to confirm a diagnosis and to identify the exact gene involved. The genetic testing strategy will depend on the clinical setting and the available resources. Diagnostic yield of karyotype, chromosomal microarray or single gene sequencing (unless there is a very clear biochemical biomarker) is in many cases very low and therefore should not be indicated as the first option. In the case of IEMs and MD, the recommended testing would be targeted next generation sequencing panels or whole exome/genome sequencing (81). The diagnostic yield of the different reported studies ranged between eleven and fifty one percent (see Supplementary Table 2). In a study by Cordeiro et al., targeted next-generation sequencing panels and whole exome sequencing increased diagnostic rate to more than 40%. In the same study, a genetic diagnosis provided either disease-specific or symptomatic therapy in thirty eight percent of the patients with a genetic diagnosis, highlighting the importance of genetic investigations to confirm underlying genetic cause in patients with pediatric MD. Early detection of treatable IEM allows for referral to expert centers and timely interventions to prevent disease progression and to improve neurological functioning. Additionally, detection of IEM for which no treatment currently exists allows for genetic counseling of the affected families (3).

## Step 5. Treatment of Pediatric Metabolic Movement Disorders

MDs are a major medical problem. Treatment of children with IEMs-associated MD may include both symptomatic treatment of MDs and disease-specific management of the underlying IEM. Pharmacologic treatments for IEMs may comprise specialized dietary modifications, cofactor, vitamins, or supplements addition, enzyme replacement therapy, hematopoietic stem cell transplantation, and gene therapy (81). Successful treatment and better outcome frequent take place with early recognition of an underlying treatable IEM, therefore there should be an increased consciousness of these IEMs (147). A complete list of the specific treatments of each IEMs appears in Supplementary Table 1. Regrettably, only thirty-eight percent of the IEMs included in this article have a disease-specific treatment. Although, over the last decades, new treatment approaches have changed the scope of IEMs from a group of rare, untreatable, and often fatal disorders to an important cause of potentially treatable diseases.

By contrast, when disease modification therapies are not possible, symptomatic therapy may be the only treatment that is available. Symptomatic therapy of MD requires multidisciplinary approach ranging from avoiding identifiable triggers, pharmacologic interventions, physiotherapy, to intrathecal baclofen pump, deep brain stimulation and gene therapy (3). Koy et al. has reviewed prodigiously the management of MD including dose range and guidelines for drugs commonly used to treat pediatric MD (148). The reader may refer to this review for more information. Even with treatment, MD hardly resolve completely and can cause life-long disability. Nevertheless, symptomatic treatment may improve the functional abilities and quality of life, correct orthopedic complications or relieve pain of children with generalized dystonia.

Recently, Mohammad et al., outlined a treatment approach for children with MD, including IEMs disorders. In this approach, phenomenology of MD, associated comorbidities (in particular intellectual disability and psychiatric disorders), etiology (and natural history of the disorder), level of functional impairment, and risk of a definite therapy are determined sequentially before making a treatment decision. As in any other MD, the administration of quality of life scales or scales for the specific MD is recommended, in order to have a useful measure of the effect of the treatment. The risk of a given treatment should always be considered before starting, since the adverse effects of the treatment may be worse than the MD itself. Providentially, side effects are transient and reversible for most treatments (149).

Keeping up to date with the emergence of new gene therapies is essential. Gene therapy trials using viral vectors results in improvement of MD and motor function in early clinical studies of AADC deficiency (150, 151).

Kojima et al., conducted an open-label phase 1/2 study including six patients whom received adeno-associated virus vector harboring DDC via bilateral intraputaminal infusions. Five of these patients presented a severe phenotype while the remaining patient presented a moderate phenotype. At follow-up for up to 2 years, motor function improved markedly in all patients. They were able to stand with support to run or ride a bicycle. The treatment was more effective in the younger patients. Cognitive and verbal functions were also improved in the patient with the moderate phenotype. Clinical signs such as dystonia and oculogyric seizures disappeared or decreased markedly. As an adverse effect, transient chorea was observed (150). Tseng et al., demonstrated that there was an improvement in the microstructural integrity of white matter tracts using Brain diffusion tensor imaging (DTI) 1 year after AADC gene therapy (151).

In the same way, enzyme-replacement therapy for CNL2 have resulted in significant less decline in motor and language function than that in historical controls although with serious adverse events comprising failure of the intraventricular device and device-related infections (152).

For many patients, medical management alone is insufficient. Hence, many of these drug-refractory patients experience significant symptomatic improvement after baclofen intrathecal infusion pump or deep brain stimulation (DBS) following an appropriate selection process. Intrathecal baclofen pump has been indicated in several patients with spasticity or severe dystonia and progressive IEMs, in some cases for palliative purposes, for example: Niemann Pick disease type C (153), Sjögren-Larsson syndrome (154), Metachromatic leukodystrophy (155), and Gaucher disease type 2 (156). Regarding DBS, Supplementary Table 4 describes the role and evidence of DBS in selected IEMs and childhood-onset MD. Twenty-five articles were included with fifty-four patients who underwent DBS surgery. The mean age of surgery was 12.2 ± 4.6 years (range: 1–18 y) and the most frequent targets were internal globus pallidus (19 studies) and subthalamic nucleus (5 studies) (157–181). In summary, DBS can be recommended for treatment of dystonia in pantothenate kinase associated neurodegeneration or Lesch–Nyhan syndrome (182). Patients with pantothenate kinase associated neurodegeneration repeatedly respond well to DBS even if there may not be sustained at long-term effect because of ongoing neurodegeneration in the pallidum. While DBS appears effective in Lesch–Nyhan syndrome patients both in reducing dystonia and self-mutilating behavior. No beneficial effect was observed in the following IEMs: X-linked adrenoleukodystrophy, methylmalonic aciduria, mitochondrial disorder and glutaric aciduria type 1.

## Step 6. Reevaluate Treatable IEMs

IEMs presenting with prominent MD can be noticed in Table 1. It is of fundamental importance to reevaluate these treatable IEMs and ensure that they have been completely discarded, both biochemically and genetically. This is a process that must be performed consistently in all patients without diagnosis. Finally, other genetic or acquired causes that could explain the movement disorders will have to be investigated.

## Future Directions

Further collaborative investigations are needed in which an elaborated description of the MD is made, including the largest cohorts possible. It would also be beneficial if more research was done on the application of intrathecal baclofen pump and deep brain stimulation in IEMs. The future is very promising, both in new diagnostic techniques in particular metabolomics and in new gene therapies. Metabolomics can be described as a complete and simultaneous analysis of small molecules in a biological sample. The aims of this analysis are to improve the diagnosis, understand the factors that contribute to the progression and susceptibility of a certain disease, and monitor the long-term metabolic effects of therapies. The scope and usefulness of this approach in diagnosing IEMs is expected to drastically change in the near future and it is very likely that they will be included in upcoming diagnostic algorithms (183).

## Conclusion

This review proposes a straightforward six-step algorithm to IEMs presenting with MD in childhood. The major components of this algorithm include the initial suspicion on an IEM, the characterization of the MD phenotype and other neurological and non-neurological signs, biochemical, genetic and imaging testing based on the type of MD. Finally, an update of the treatable IEMs and the therapies available for the MD is made. The application of this algorithm will potentially allow clinicians to avoid blind and poorly efficacious explorations or biological test.

## Author Contributions

JDO-E wrote and reviewed the final manuscript and provide data for all tables and figures.

## Conflict of Interest

The author declares that the research was conducted in the absence of any commercial or financial relationships that could be construed as a potential conflict of interest.
