# Supplementary material for: A Proposed Diagnostic Algorithm for Inborn Errors of Metabolism Presenting With Movements Disorders
Source: Front Neurol. 2020 Nov 13;11:582160. doi: 10.3389/fneur.2020.582160 (PMC7691570; doi:10.3389/fneur.2020.582160)
Supplement: Supplementary file 1 [file Table_1.DOCX]

Table 1. Movement disorders and neurological/non-neurological characterization, biochemical findings, neuroimaging and disease-specific treatment in IEMs with MD as a prominent feature.

| **Diseases** | **Gene (OMIM)** | **MD Phenotype** | **Other clinical features** | **Biochemical findings** | **Neuroimaging** | **specific Treatment** | **Ref.** |
| --- | --- | --- | --- | --- | --- | --- | --- |
| DISORDERS OF NITROGEN-CONTAINING COMPOUNDS | | | | | | | |
| Guanidinoacetate methyltransferase deficiency | *GAMT*  (#612736) | Dystonia and choreoathetosis | ID, hypotonia, S, hyperreflexia | ↓CSF creatine, ↓CSF creatinine, ↓ creatine excretion | BG hyperintensities | Creatine and ornithine supplementation, arginine restriction | 137 |
| Creatine transporter deficiency | *SLC6A8*  (#300352) | Early onset dystonia (3 month of age) worsened by infections, oromandibular dystonia. Choreiform movements (33 month of age) | Failure to thrive, microcephaly, ID, hypotonia, spasticity, S, behavioral abnormalities | ↑urinary and plasma creatine, ↑urinary creatine-to-creatinine ratio | BA, DM, TCC, ↓creatine peak on MRS | Creatine, arginine and glycine supplementation | 81 |
| Tyrosine hydroxylase deficiency | *TH*  (#605407) | There have been described three phenotypes: (1) TH-deficient dopa-responsive dystonia (the mild form of TH deficiency) age onset: 12 months to 12 years of age, initial symptoms are typically lower-limb dystonia and/or difficulty in walking (2) TH-deficient infantile HRS (hypokinesia, rigidity of extremities, and/or tremor) with motor delay (the severe form), age at onset: 3 to 12 months, and (3) TH-deficient progressive infantile encephalopathy (the very severe form), age at onset: 3 to 6 months: severe hypokinesia and limb hypertonia. | Oculogyric crisis, ptosis, truncal hypotonia | ↓CSF HVA, ↓CSF MHPG, normal CSF 5-HIAA | Normal, TH-deficient progressive infantile encephalopathy present mild brain or cerebellar atrophy or periventricular WM changes | L-dopa | 138 |
| Aromatic L-amino acid decarboxylase deficiency | *DDC*  (#608643) | MD most often described are oculogyric crises (77%), dystonia (53%), and hypokinesia (34%). Dyskinesia (e.g. hyperkinesia, chorea, athetosis), myoclonus and tremor have also been described. Status dystonicus can occur in AADCD patients. | Oculogyric crisis, ptosis, nasal congestion, hypotension, poor feeding, gastroesophageal reflux disease, ID, truncal hypotonia, hyperreflexia, sleep disturbances, paroxysmal sweating, temperature instability | ↓CSF HVA, ↓CSF 5-HIAA, ↓ whole blood serotonin, ↑CSF, plasma and urinary L-Dopa, 5HTP, 3-OMD and 3-methoxytyrosine | Mild brain atrophy | Dopamine agonists, MAO inhibitors, pyridoxine, AAV-delivered gene therapy targeted to the SN | 76 |
| Dopamine transporter deficiency | *SLC6A3*  (#613135) | Classical DTDS: nonspecific findings following by hyperkinetic MD (with features of chorea, dystonia, ballismus, orolingual dyskinesia). Atypical DTDS: Normal psychomotor development in infancy and early childhood is followed by later onset manifestations of parkinsonism-dystonia with tremor, progressive bradykinesia, variable tone, and dystonic posturing. | Oculogyric crisis, ocular flutter, gastroesophageal reflux disease, poor feeding, truncal hypotonia | ↑CSF HVA, normal CSF 5-HIAA | Normal | Dopamine agonists | 139 |
| Dopamine-serotonin vesicular transport defect | *SLC18A2*  (#618049) | Gait dystonia, HRS, and oculogyric crises have been described. | Oculogyric crisis, ptosis, nasal congestion, increased sweating, poor distal perfusion (cold hands and feet), truncal hypotonia, ID, hyperreflexia, hypernasal speech, temperature instability | ↑urinary HVA and 5-HIAA, ↓urinary norepinephrine and dopamine, normal CSF neurotransmitters | Normal | Dopamine agonists | 140 |
| Autosomal recessive and autosomal dominant GTP cyclohydrolase I deficiency | *GCH1*  (#233910)  *GCH1*  (#128230) | Childhood-onset dystonia and a dramatic and sustained response to low doses of oral administration of L-dopa. This disorder typically presents with gait disturbance caused by foot dystonia, later development of HRS signs. Occasionally, initial symptoms are arm dystonia, postural tremor of the hand, or slowness of movements. In general, gradual  progression to generalized dystonia is observed. | Abnormal ocular movement, ID, truncal hypotonia, S, episodic hyperthermia, torticollis, pes cavus | ↑plasma phenylalanine, ↓CSF HVA and normal or ↓ 5-HIAA and ↓CSF biopterin and neopterin | Normal | Low Phe/BH4 supplementation, L-dopa, 5-hydroxytryptophan | 82 |
| Sepiapterin reductase deficiency | *SPR*  (#612716) | Dystonia and oculogyric crisis are present >65% of patients. Other common features include HRS signs (tremor, bradykinesia, masked facies, rigidity), choreoathetosis and ataxia | Oculogyric crisis, oculomotor apraxia, microcephaly, ID, S, autonomic signs, sleep disturbances | ↓CSF 5-HIAA, ↓CSF HVA, ↑CSF biopterin, ↑CSF dihydrobiopterin, ↑CSF Sepiapterin, ↓urinary HVA, 5-HIAA and VMA | Normal, rarely BA, DM | L-dopa, 5-hydroxytryptophan, BH4 | 141 |
| DNAJC12-deficient hyperphenylalaninemia | *DNAJC12* (#617384) | Progressive MD with prominent dystonia | Nystagmus, oculogyric crisis, hypotonia, ID, autism | ↑serum phenylalanine, ↓CSF dopamine and serotonin metabolites | --- | BH4, L-dopa and/or 5- hydroxytryptophan | 142 |
| 2-Methyl-3-hydroxybutyryl-CoA dehydrogenase deficiency | *HSD17B10* (#300438) | Generalized rigidity with some dystonic posturing at 15 years of age. Tremor can appear later in the disease course. | SNHL, optic atrophy, retinal degeneration, nystagmus, hypertrophic cardiomyopathy, ID, hypotonia, S, spasticity | ↑blood lactate, metabolic acidosis, ↑urinary 2-methyl-3 hydroxybutyrate, ↑urinary tiglylglycine | --- | None | 143 |
| Glutaric aciduria type 1 | *GCDH*  (# 231670) | Progressive complex MD including dystonia and choreoathetosis. Dystonia is a significant sequela for individuals with BG injury. Those who have insidious onset generally have less severe MD. | Failure to thrive, macrocephaly, hepatomegaly, S | Glutaricaciduria, metabolic acidosis, ketonemia, ketonuria, hypoglycemia | BG hyperintensities, frontotemporal atrophy, DM, dilatation of lateral ventricles | Carnitine, lysine-restricted/arginine- rich diet | 54 |
| DISORDERS OF VITAMINS, COFACTORS, METALS AND MINERALS | | | | | | | |
| Folate receptor alpha deficiency | *FOLR1*  (# 613068) | Intention tremor, progressive ataxia and epileptic myoclonus are typically found at diagnosis (median onset 2 years, range 6 mo. to 4,5 y). Later, they develop choreoathetosis. | ID, hypotonia, congenital microcephaly, PN, S, sensory stimulus-sensitive drop attacks | ↓CSF methyltetrahydrofolate | BG calcification, DM, BA | Folinic acid | 96 |
| Biotin-thiamine-responsive basal ganglia disease | *SLC19A3*  (#607483) | Dystonia was the second most common sign after encephalopathy. The BFMDS questionnaire was administered to 34 SLC19A3 patients with dystonia (9.8 6 1.8 points [mean 6 SEM]; range, 0–30). Higher BFMDS scores were identified in patients who had a previous history of developmental delay and in patients with disease onset before 6 months of age. A positive, and almost significant, correlation was observed between the BFMDS scores and the time from disease onset to thiamine initiation | Nystagmus, ophthalmoplegia, ptosis, episodic encephalopathy, S, truncal hypotonia, spasticity | Metabolic and lactic acidosis, ↓CSF free thiamine | BG hyperintensities | Thiamine, biotin | 87 |
| Pantothenate kinase-associated neurodegeneration (PKAN) | *PKAN2*  (#234200) | Choreoathetosis, dystonia, including severe jaw-opening dystonia, HRS. Dystonia is always present and usually an early manifestation. Cranial dystonia and limb dystonia are frequent and may lead, respectively, to recurrent trauma to the tongue and to atraumatic long bone fracture from the combination of extreme bone stress and osteopenia. The resulting pain and distress can contribute to development of status dystonicus in a cycle that can be difficult to break. | Pigmentary retinopathy, optic atrophy, ID, spasticity, psychiatric abnormalities | None | Eye of the tiger sign, BA | None | 14 |
| Alpha-tocopherol transfer protein deficiency | *TTPA*  (#277460) | Late childhood or early teens between ages five and 15 years (age range: 2 to 37 years of age) progressive ataxia and clumsiness of the hand. Head tremor in 40% of cases. | Areflexia, proprioception loss, xanthelasmata, tendon xanthomas | ↓blood vitamin E, ↑serum cholesterol, triglycerides and beta-lipoprotein | Normal, CeA | Vitamin E | 93 |
| Wilson disease | *ATP7B*  (#277900) | Neurologic involvement follows two general patterns: movement disorders or rigid  dystonia.  • Movement disorders tend to occur earlier and include tremors, poor coordination, loss of fine-motor  control, micrographia (abnormally small, cramped handwriting), chorea, and/or choreoathetosis.  • Spastic dystonia disorders manifest as mask-like facies, rigidity, and gait disturbance | Kayser-Fleischer ring, hepatomegaly, cirrhosis, renal tubular dysfunction, osteoporosis, chondrocalcinosis, PN | Hemolytic anemia, hypoparathyroidism, ↓serum ceruloplasmin, ↑urinary copper, proteinuria, aminoaciduria, glucosuria, hypercalciuria, hyperphosphaturia | Face of the Panda sign | Zinc, penicillamine, trientine | 10 |
| SLC30A10 deficiency | *SLC30A10*  (#613280) | Although most cases show pure four-limb dystonia leading to a characteristic high stepping gait (a ‘‘cock-walk’’ gait) and fine motor impairment sometimes accompanied by dysarthria, fine tremor, and bradykinesia, one affected individual has pure spastic paraparesis without extrapyramidal dysfunction | Hepatomegaly, cirrhosis, PN | Polycythemia, ↑blood manganese, ↑unconjugated bilirubin, ↑transaminases, ↑erythropoietin, ↓iron, ↓ferritin, ↑TIBC | BG T1W hyperintensities | Chelation (EDTA), iron supplementation | 53 |
| SLC39A14 deficiency | *SLC39A14*  (#617013) | Affected children presented with loss of developmental milestones, progressive dystonia and bulbar dysfunction in infancy or early childhood. Towards the end of the first decade, they developed severe generalized pharmacoresistant dystonia, spasticity, limb contractures and scoliosis, and lost independent ambulation. Some showed parkinsonian features of hypomimia, tremor and bradykinesia | Microcephaly, scoliosis, ID, spasticity, hyperreflexia, ankle clonus, | ↑blood manganese | BG T1W hyperintensities, BA, CeA | Chelation (EDTA) | 16 |
| DISORDERS OF CARBOHYDRATES | | | | | | | |
| Glucose transporter 1 deficiency | *SLC2A1* (*138140) | A complex MD is commonly seen and is characterized by ataxia, dystonia, and chorea that may be continuous, paroxysmal, or continual with fluctuations determined by environmental stressors. Often, paroxysmal worsening occurs before meals, during fasting, or with infectious stress.  Clinical findings included the following: Gait disturbance (89%), the most frequent being ataxia and spasticity together or ataxia alone, action limb dystonia (86%), mild chorea (75%),  cerebellar action tremor (70%), non-epileptic paroxysmal events (28%), dyspraxia (21%) and myoclonus (16%)  Paroxysmal movement disorders. Paroxysmal exercise-induced dyskinesia and paroxysmal choreoathetosis are now recognized to be part of the phenotypic spectrum of Glut1 DS.  Other associated findings included progressive spastic paraparesis with onset in early adulthood, mild gait ataxia, mild-to-moderate cognitive impairment, and epileptic seizures.  It is unclear whether these events represent epileptic or non-epileptic phenomena. | Microcephaly, S, hyperreflexia, ID | ↓CSF glucose, ↓CSF lactate | Normal | Ketogenic diet, triheptanoin | 13 |
| Pyruvate carboxylase deficiency | *PC*  (# 266150) | A, D, HRS, T | Hepatomegaly, proximal renal tubular acidosis, ID, hypotonia, S, ankle clonus | ↑blood lactate, pyruvate and alanine, hypoglycemia, ↑serum ammonia, citrulline and lysine, ↑lactate: pyruvate ratio | BA, periventricular cysts and leukomalacia, DM, subcortical leukodystrophy | None | 144 |
| MITOCHONDRIAL DISORDERS OF ENERGY METABOLISM | | | | | | | |
| Pyruvate dehydrogenase complex deficiency | Various genes | Isolated paroxysmal exercise induced dystonia and intermittent isolated ataxia have been described. Late onset (mid-thirties) atypical parkinsonism, choreiform movements, stereotypies, ataxia have also been reported. | Low birth weight, microcephaly, episodic ptosis, hypotonia, ID, S, facial dysmorphism (less frequent) | ↑blood and CSF lactate and pyruvate, ↑blood alanine, ammonia | BG T2W hyperintensities, BA, agenesis of corpus callosum, ↑lactate on MRS | Thiamine, ketogenic diet | 64-67 |
| Succinyl-CoA ligase β subunit (SUCLA2) deficiency | *SUCLA2* (#612073) | Early-onset dystonia/hyperkinesia-deafness syndrome. Dystonia in 85% of patients. | Failure to thrive, SNHL, ophthalmoplegia, ptosis, strabismus, hypotonia, ID, spasticity, hyporeflexia, S, PN | ↑blood and CSF lactate, ↑CK, methylmalonic aciduria, methylglutaconic aciduria, intermittent aminoaciduria | BG T2W hyperintensities, BA | None | 39 |
| Succinyl-CoA ligase α subunit (SUCLG1) deficiency | *SUCGL1*  (# 245400) | Dystonia (40% of patients) | Failure to thrive, SNHL, hypotonia, ID | ↑blood and CSF lactate, hypoglycemia, methylmalonic aciduria, abnormal mitochondrial RCC activities | BG T2W hyperintensities, BA | None | 145 |
| L-2-hydroxyglutaric aciduria | *L2HGDH*  (# 236792) | Dystonia, ataxia and intention tremor have been reported | SNHL, optic atrophy, strabismus, nystagmus, ID, S | ↑serum lysine, ↑ serum, urinary and CSF L-2-hydroxyglutaric acid | Leukoencephalopathy with cavitation, BA, CeA | None | 27, 146 |
| Leigh Syndrome | >75 genes | Dystonia is a common feature in Leigh syndrome. Choreic movements have been found in Leigh syndrome, especially in children with ATPase 6 point mutations. Myoclonus has also been reported in some patients. | Failure to thrive, ophthalmoplegia, optic atrophy, nystagmus, strabismus, ptosis, pigmentary retinopathy hypotonia, hypertrichosis, ID, spasticity, hyperreflexia, S | ↑blood and CSF lactate | BG, cerebellum and brainstem T2W hyperintensities | None | 22 |
| Myoclonic epilepsy with ragged red fibers (MERRF) | Various genes | Ataxia and myoclonus | S, spasticity, SNHL | ↑blood lactate, ↑pyruvate | CeA, atrophy of superior cerebellar peduncles | None | 147 |
| Neuropathy, ataxia and retinitis pigmentosa (NARP) | MTATP6  (*516060) | Proximal neurogenic muscle weakness with sensory neuropathy, ataxia, and pigmentary retinopathy. Symptoms usually start in childhood. | Retinitis pigmentosa, nystagmus, ID, S, PN | None | Normal, BA, CeA | None | 148 |
| MEGDEL Syndrome | *SERAC1* (**#** 614739) | Starting at a median age of 6 months, muscular hypotonia (91%) was seen, followed by progressive spasticity (82%, median onset 5 15 months) and dystonia (82%, 18 months). The majority of affected individuals never learned to walk (68%). | Failure to thrive, microcephaly, SNHL, optic atrophy, neonatal hepatic dysfunction, hypotonia, ID, spasticity, S, recurrent infections, neonatal sepsis | ↑blood lactate, ↑transaminases, ↑AFP, hypoglycemia, coagulopathy, 3-methylglutaconic aciduria, ↓cholesterol | BG T2W hyperintensities sparing central putamen, BA, CeA | None | 39 |
| DNAJC19 deficiency | *DNAJC19*  (#610198) | Non-progressive cerebellar ataxia | Prenatal growth failure, optic atrophy, dilated cardiomyopathy, long QT syndrome, steatosis, hypospadias, cryptorchidism, ID | Microcytic anemia, ↑transaminases,  3-methylglutaconic aciduria, 3-methylglutaric aciduria | BG T2W hyperintensities | None | 149 |
| Mohr-Tranebjaerg syndrome | *TIMM8A* (**#** 304700) | Deafness-dystonia-optic neuronopathy (DDON) syndrome is a progressive disorder. Dystonia and ataxia may appear in adolescent. One case of rapidly progressive dystonia who died at 16 years of age. | SNHL, cortical blindness, fractures, spasticity, hyperreflexia, mental regression, behavioral abnormalities | None | BG atrophy in males older than 40 years of age | None | 17 |
| CLPB deficiency | *CLPB*  (#616271) | Severe cases with hyperekplexia or absence of voluntary movements in the neonatal period. Moderate cases with progressive movement disorder (ataxia, dystonia, and/or dyskinesia) of varying severity | Failure to thrive, microcephaly, cataracts, neonatal hypotonia, ID, spasticity, S, recurrent infections | Neutropenia, ↑urinary 2-methylglutaconic acid | BG atrophy, BA, CeA | None | 150 |
| Sacsin deficiency | *SACS*  (#270550) | Mild to moderate early onset gait ataxia (age at onset 16-18 months of age) (33%), tremor (10%) | Nystagmus, pes cavus, PN, spasticity, hyperreflexia, ID (rare) | None | Vermis atrophy | None | 151 |
| DISORDERS OF LIPIDS | | | | | | | |
| Mitochondrial enoyl-coA reductase deficiency | *MERC* (#617282) | Childhood-onset progressive dystonia, facial chorea, dyskinesias and myoclonus: ages 1-6.5 years | Optic atrophy, nystagmus, spasticity, hyperreflexia | ↑CSF lactate, abnormal mitochondrial RCC activities | BG hyperintensities | None | 152 |
| ELOVL4 deficiency | *ELOVL4* (#133190) | AD inheritance: adult onset (30-40 years of age) slowly progressive ataxia, pyramidal tract signs, and cerebellar and pontine atrophy detected on MRI, erythrokeratodermia may be present. AR inheritance: spastic paraplegia, ichthyosis and ID. | Nystagmus, supranuclear gaze palsy, erythrokeratodermia, spasticity, PN | None | CeA, pontine atrophy, pontine midline linear hyperintensity | None | 153 |
| PLA2G6-associated neurodegeneration | *PLA2G6*  (*603604) | PLAN encompasses a continuum of three overlapping phenotypes: 1) infantile onset PLAN, corresponding to classic infantile neuroaxonal dystrophy, 2) childhood-onset PLAN corresponding to atypical neuroaxonal dystrophy (ANAD) and 3) juvenile adult-onset PLAN corresponding to PLA2G6-related dystonia-parkinsonism. These patient exhibit predominantly HRS, resting tremor and limbs, oromandibular or generalized dystonia. | Psychomotor regression, hypotonia, pyramidal tract signs, a/hyperreflexia, S, PN | None | CeA, BA, cerebellar cortex T2W hyperintensities, thin optic chiasm, BG iron deposition |  | 24 |
| Fatty acid hydroxylase-associated neurodegeneration | *FA2H* (#612319) | Variable phenotype NBIA, SPG35 (spastic paraparesis) and leukodystrophy (dystonia). Early childhood onset predominantly lower limb spastic tetraparesis and truncal instability, cerebellar ataxia, and cognitive deficits, often accompanied by movement disorders. The disease is rapidly progressive with loss of ambulation after a median of 7 years after disease onset | Optic atrophy, nystagmus, external ophthalmoplegia, spasticity, hyperreflexia, S | None | TCC, CeA, brainstem atrophy, WM abnormalities, iron deposition in globus pallidus | None | 15 |
| STORAGE DISORDERS | | | | | | | |
| Beta-propeller protein-associated neurodegeneration (BPAN) | *WDR45*  (# 300894) | The affected individuals universally showed an early-onset global developmental delay that was static until adolescence/early adulthood when a secondary neurological decline was noted including HRS, dystonia and dementia | Eye movement abnormalities, retinal atrophy, ID, S, behavioral abnormalities | None | Globus pallidus T1W hyperintensities, BA, CeA | None | 25 |
| Sialidosis | NEU1  (*608272) | Slowly progressive ataxia and myoclonus (age at onset: 9 years of age) | Coarse facies, SNHL, nystagmus, cherry-red spot, lens opacity, cardiomyopathy, neonatal ascites, hepatomegaly, splenomegaly, inguinal hernia, dysostosis multiplex, muscle weakness, S, ID, hypotonia, hyperreflexia, hydrops fetalis | Vacuolated lymphocytes, proteinuria, ↑ urine sialyloligosaccharides and sialylglycopeptides | Normal | None | 154 |
| Galactosialidosis | CSTA  (#256540) | Juvenile/adulthood phenotype (Japanese patients): myoclonus and ataxia | Coarse facies, SNHL, ID, S, dysostosis multiplex, corneal clouding, red cherry-spot | ↑urine sialyloligosaccharides, normal sialic acid | Enlarged ventricles, hyperintense WM, striato thalamic vasculopathy, widened periencephalic spaces | None | 155 |
| Niemann-Pick disease type C | *NPC1*(#257220)  *NPC2*(#607625) | Prominent MD: typically beginning as action dystonia in one limb and gradually spreading to involve all of the limbs and axial muscles. Speech gradually deteriorates, with a mixed dysarthria and dysphonia. Facial, orolingual and limbs severe dystonia, cerebellar truncal and limbs ataxia, gelastic cataplexy. Slowly progressive course. Age at onset: 10 to 14 years of age. | Vertical supranuclear gaze palsy, hepatomegaly, splenomegaly, neonatal jaundice, fetal ascites, hypotonia, ID, spasticity, S, cataplexy, behavioral alterations | Enzyme analysis, Filipin test |  | None | 12,40 |
| Action myoclonus-renal failure syndrome | *SCARB2* (**#** 254900) | Adolescent–young adulthood onset, progressive action myoclonus, ataxia and tremor, absence of mental deterioration | Nephrotic syndrome, renal failure, S | Thrombocytopenia, proteinuria | Thrombocytopenia, proteinuria | None | 156 |
| DISORDERS OF PEROXISOMES | | | | | | | |
| Sterol carrier protein-2 deficiency | *SCP2* (*184755) | Spasmodic torticollis and dystonic head tremor, at 7 years of age as well as slight cerebellar signs with intention tremor. Dystonic head tremor triggered by stressful situations | Abnormal eye movements, azoospermia | Hypergonadotropic hypogonadism | WM hyperintensities, butterfly lesions of the pons | None | 69 |
| CONGENITAL DISORDERS OF GLYCOSYLATION | | | | | | | |
| Phosphomannomutase 2 deficiency | *PMM2* (#212065) | Prominent cerebellar ataxia with generalized muscular hypotonia, generalized or segmental/multifocal dystonia, choreoathetosis. Stereotypies and tremor have also been described | Nonimmune hydrops fetalis, ID, hypotonia, S, hyporeflexia, stroke-like episodes, PN, abnormal subcutaneous fat tissue distribution, inverted nipples, joint contractures, hepatomegaly, nephrotic syndrome, cardiomyopathy, retinitis pigmentosa, nystagmus, microcephaly, failure to thrive | ↑transaminases, thrombocytosis, ↓IgA, ↓IgG, ↓Cu, ↓Fe, ↓Zn, ↓cholesterol, ↓albumin, ↓Factor XI, ↓antithrombin III, abnormal TIFT (type 1 pattern), proteinuria, prolonged prothrombin time, hypergonadotropic hypogonadism, hypothyroidism | Olivopontocerebellar hypoplasia, CeA | None | 41 |
| ST3GAL5-CDG | *ST3GAL5* (*604402) | Choreoathetoid and dystonic cerebral palsy, startle myoclonus onset between 2 weeks and 3 months before GTCS seizures | Failure to thrive, microcephaly, deafness, optic atrophy, ID, S, hypotonia | None | Diffuse BA | None | 157, 158 |
| Steroid 5 alpha-reductase 3 deficiency | *SRD5A3*  (*611715) | Early onset cerebellar ataxia (onset before 3,5 years of age) | ID, hypotonia | ↑transaminases, ↓IGF1, ↓IGFBP3, microcytic anemia, coagulation defects, antithrombin III deficiency, abnormal TIFT (type 1 pattern) | Polymicrogyria or cerebellar vermis hypoplasia | None | 159 |
| GOSR2-CDG | *GORS2* (#614018) | Early onset ataxia (on average at 2 years of age), action myoclonus and myoclonic seizures (onset average at 6,5 years), rest tremor. Transient episodes of motor deterioration triggered by infection and fever. | Mild ID, S, scoliosis | ↑CK | Normal | None | 62 |
| N-glycanase 1 deficiency | *NGLY1* (*610661) | All affected individuals exhibited choreoathetosis, dystonia, myoclonus and action tremor. These movements were more severe in younger individuals. | ID, PN, S, hypotonia, microcephaly | ↑transaminases, ↑AFP, ↑blood lactate, abnormal urine oligosaccharides, normal TIFT, normal N-glycan analysis | Normal to BA, DM, or prominent Virchow Robin spaces | None | 160 |

**Abbreviations.** A= ataxia, AAV= adeno-associated virus, AFP = alpha-fetoprotein, BA = brain atrophy, BG = basal ganglia, CA= choreoathetosis, CeA= Cerebellar atrophy CK = creatine kinase, D= dystonia, DM = delayed myelination, 5-HIAA = 5-hydroxyindoleacetic acid, HRS= hypokinetic rigid syndrome, 5HTP = 5-hydroxytryptophan, HVA= homovanillic acid, ID = intellectual disability, M = myoclonus, MHPG = 3-methoxy-4-hydroxyphenylethyleneglycol, 3-OMD = 3-ortho-methyldopa, PN = peripheral neuropathy, RCC= respiratory chain complex S= seizures, SNHL = sensorineural hearing loss, T= tremor, TCC= thin corpus callosum, TIBC = total iron binding capacity, TIFT = transferrin isoelectric focusing test, VMA = vanillyl mandelic acid, WM = white matter
