# Supplementary material for: A Proposed Diagnostic Algorithm for Inborn Errors of Metabolism Presenting With Movements Disorders
Source: Front Neurol. 2020 Nov 13;11:582160. doi: 10.3389/fneur.2020.582160 (PMC7691570; doi:10.3389/fneur.2020.582160)
Supplement: Supplementary file 2 [file Table_2.DOCX]

| **Table 2. IEMs presenting with MD by age at onset** | | | |
| --- | --- | --- | --- |
| **Prenatal** | **Neonatal** | **Infancy and childhood** | **Adolescence and adulthood** |
| - Atypical Gaucher disease due to saposin C deficiency ^161^ - Glycine encephalopathy^60,61^ | - Hyperekplexia or hyperexcitability: SUOX - Isolated sulfite oxidase deficiency^162^ - Absence of voluntary movements or HRS: CLPB 3-methylglutaconic aciduria disorder^150^, ADSL adenylosuccinate lyase deficiency ^163^ and PC pyruvate carboxylase deficiency^164^ - Myoclonic jerks: GLDC and AMT - glycine encephalopathy^60,61^ - Tremor, jitteriness, dystonia: HTRA2 3-methylglutaconic aciduria type 8 ^165^ | **Most of the IEMS presenting with MD begin in this age group**  Onset before 2 years of age: disorders of purine and creatine metabolism,^81,137^ neurotransmitters disorders^,76,82,138-141^ propionic^56^ and methylmalonic acidemia,^175^ glutaric aciduria type 1,^54^ disorders of cobalamin metabolism,^38^ biotinidase deficiency,^174^ manganese disorders *(SLC39A8*),^173^ GLUT-1 deficiency,^13^ mitochondrial disorders (including Leigh syndrome),^22^ SNX14 deficiency^,20^ CLN14 disease,^40^ Niemann Pick type C,^12-40^ Sialidosis,^154^ PMM2-CDG^41^ and other congenital disorders of glycosylation.^62, 157-160^ | - adult-onset ataxia: 3-methylglutaconyl-CoA hydratase deficiency,^166^ 3-phosphoglycerate dehydrogenase deficiency^167^, γ-glutamylcysteine synthetase deficiency,^168^ OPA1 deficiency, ^169^ very long-chain fatty acid elongase 4 deficiency,^153^ very long-chain fatty acid elongase 5 deficiency ,^170^ abetalipoproteinemia,^171^ hereditary coproporphyria,^68^ and complex MD (tremor, ataxia, myoclonus, perioral dyskinesias) cathepsin F deficiency,^172^ (myoclonus, cerebellar ataxia, parkinsonism) Neuronal ceroid lipofuscinosis type 4 (Parry type) ^23^ |

- To be noted that many IEMs presenting with MD do not have a specific age of onset, hence usually this parameter may not be extremely valuable in evaluating a particular case.
