# Supplementary material for: A Proposed Diagnostic Algorithm for Inborn Errors of Metabolism Presenting With Movements Disorders
Source: Front Neurol. 2020 Nov 13;11:582160. doi: 10.3389/fneur.2020.582160 (PMC7691570; doi:10.3389/fneur.2020.582160)
Supplement: Supplementary file 3 [file Table_3.DOCX]

| **Table 3. IEMs presenting with paroxysmal MD** | | | |
| --- | --- | --- | --- |
| **Ataxia** | **Dystonia** | **Chorea** | **Dyskinesia** |
| - pyruvate dehydrogenase complex deficiency ^64-67^ - BTD-biotinidase deficiency^174^ - Hartnup disease^176^ - GLDC and AMT-glycine encephalopathy^60,61^ - HTD-Tyrosinemia type III^177^ - SLC2A1-GLUT1 deficiency^13^ | - SLC2A1-GLUT1 deficiency^13^ - ECHS1-mitochondrial short-chain enoyl-CoA hydratase 1 deficiency^63^ - HIBCH-3-hydroxyisobutyryl-CoA hydrolase deficiency ^178^ - pyruvate dehydrogenase complex deficiency ^64-67^ | - OTC- Ornithine transcarbamylase deficiency ^179-180^ | - ABAT-GABA transaminase deficiency ^181^ - ALDH5A1-Succinic Semialdehyde Dehydrogenase Deficiency^182^ - PARK2-Parkin deficiency^183^ |
