# Supplementary material for: A Proposed Diagnostic Algorithm for Inborn Errors of Metabolism Presenting With Movements Disorders
Source: Front Neurol. 2020 Nov 13;11:582160. doi: 10.3389/fneur.2020.582160 (PMC7691570; doi:10.3389/fneur.2020.582160)
Supplement: Supplementary file 4 [file Table_4.DOCX]

| Table 4. Minimal biochemical testing to diagnose treatable IEMs based on the particular MD | |
| --- | --- |
| Ataxia | Dystonia |
| - Blood: 1^st^ Tier: hemoglobin, reticulocytes, blood count, ASAT/ALAT, glucose, uric acid, urea, creatine, guanidino compounds, ammonia, lactate, pyruvate, glutathione, amino acids, orotic acid, total homocysteine, acylcarnitines, methylmalonic acid, vitamin B12, folate, thiamine pyrophosphate, vitamin E, copper, ceruloplasmin, ferritin, manganese, VLCFA, sialotransferrins, CoQ10, acetoacetate, sterols. 2^nd^ Tier: Galactose-1-P, GALT enzyme activity, TPP1 enzyme activity, beta-glucosidase enzyme analysis, arylsulfatase A enzyme analysis, alpha-mannosidase enzyme analysis, porphobilinogen, aminolevulinic acid, porphyrins, phytanic acid, pristanic acid, plasmalogens - Urine: 1^st^ Tier: uric acid, purines and pyrimidines, creatine, guanidino compounds, organic acids, thiosulfate, sulfites, copper, acetoacetate, sulfatide, oligosaccharides, pipecolic acid 2^nd^ Tier: porphobilinogen, aminolevulinic acid, porphyrins, - CSF: 1^st^ Tier: neurotransmitters, amino acids, 5-Methyl-THF, free thiamine, pipecolic acid, glucose, lactate, protein, - Fibroblasts: 2^nd^ Tier: Cyclic NADHX - Feces: 2^nd^ Tier: porphyrins   *Celia's encephalopathy | - Blood: 1^st^ Tier: blood count, ASAT/ALAT, glucose, uric acid, creatine, guanidino compounds, prolactin, amino acids, pterins, total homocysteine, lactate, pyruvate, acylcarnitines, methylmalonic acid, thiamine pyrophosphate, pipecolic acid, AASA, copper, ceruloplasmin, ferritin, manganese, sialotransferrins, CoQ10, acetoacetate, sterols, VLCFA. 2^nd^ Tier: Galactose-1-P, GALT enzyme activity, TPP1 enzyme activity, beta-glucosidase enzyme analysis, arylsulfatase A enzyme analysis, - Urine: 1^st^ Tier: uric acid, purines and pyrimidines, creatine, guanidino compounds, pterins, organic acids, thiosulfate, pipecolic acid, AASA, sulfites, copper, acetoacetate, sulfatide, - CSF: 1^st^ Tier: neurotransmitters, pterins, free thiamine, PLP, pipecolic acid, AASA, glucose, lactate, protein,   *Celia's encephalopathy |
| Choreoathetosis | **Tremor** |
| - Blood: 1^st^ Tier: blood count, ASAT/ALAT, glucose, lactate, pyruvate, uric acid, creatine, guanidino compounds, prolactin, amino acids, pterins, purine, total homocysteine, copper, ceruloplasmin, acylcarnitines, methylmalonic acid, folate, sterols, acetoacetate. 2^nd^ Tier: Galactose-1-P, GALT enzyme activity, TPP1 enzyme activity - Urine: 1^st^ Tier: glucose, lactate, purine and pyrimidines, guanidino compounds, pterins, organic acids, sulfites, copper, acetoacetate - CSF: 2^nd^ Tier: amino acids, neurotransmitters, pterins, 5-Methyl-THF, pipecolic acid | - Blood: 1^st^ Tier: blood count, glucose, prolactin, ASAT/ALAT, copper, ceruloplasmin, ferritin, manganese, amino acids, acylcarnitines, AASA, pipecolic acid, CoQ10, acetoacetate, sterols. 2^nd^ Tier: Galactose-1-P, GALT enzyme activity, beta-glucosidase enzyme analysis - Urine: 1^st^ Tier: copper, pterins, organic acids, AASA, pipecolic acid, acetoacetate - CSF: 2^nd^ Tier: glucose, lactate, neurotransmitters, pterins, 5-Methyl-THF, PLP, AASA, pipecolic acid   *Celia's encephalopathy |
| Myoclonus | **HRS** |
| - Blood: 1^st^ Tier: blood count, glucose, ASAT/ALAT, urea, prolactin, copper, ceruloplasmin, ammonia, amino acids, orotic acids, total homocysteine, AASA, pipecolic acid, CoQ10, sterols. 2^nd^ Tier: beta-glucosidase enzyme analysis, TPP1 enzyme activity - Urine: 1^st^ Tier: copper, amino acids, orotic acid, pterins, AASA, pipecolic acid - CSF: 2^nd^ Tier: glucose, lactate, neurotransmitters, pterins, PLP, AASA, pipecolic acid   *Celia's encephalopathy | - Blood: 1^st^ Tier: Blood count, glucose, ASAT/ALAT, prolactin, copper, manganese, ceruloplasmin, ferritin, uric acid, total homocysteine, amino acids, acylcarnitines, sterols. 2^nd^ Tier: Galactose-1-P, GALT enzyme activity, beta-glucosidase enzyme analysis - Urine: 1^st^ Tier: copper, purines, pterins, organic acids, sulfites - CSF: 1^st^ Tier: glucose, lactate, neurotransmitters, pterins, 5Methyl-THF, pipecolic acid |

*Celia's encephalopathy - BSCL2 deficiency requires genetic testing
