# Supplementary material for: A Proposed Diagnostic Algorithm for Inborn Errors of Metabolism Presenting With Movements Disorders
Source: Front Neurol. 2020 Nov 13;11:582160. doi: 10.3389/fneur.2020.582160 (PMC7691570; doi:10.3389/fneur.2020.582160)
Supplement: Supplementary file 5 [file Table_5.DOCX]

| **Table 5. Radiological findings in IEMs with MD** | | | | | | |
| --- | --- | --- | --- | --- | --- | --- |
| **NORMAL MRI** | **BRAIN** | **CEREBELLUM** | **CORPUS CALLOSUM** | **WHITE MATTER** | **BASAL GANGLIA** | **OTHERS** |
| Phosphoribosyl pyrophosphate synthetase 1 superactivity  Isovaleric acidemia  Glycine encephalopathy due to aminomethyltransferase deficiency  Imerslund-Gräsbeck syndrome  cblC disease  Glucose transporter 1 deficiency  Celia's encephalopathy  Alpha-tocopherol transfer protein deficiency  Coproporphyrinogen oxidase deficiency  Holocarboxylase synthetase deficiency  Tyrosine hydroxylase deficiency  Aromatic L-amino acid decarboxylase deficiency  Dopamine transporter deficiency  Dopamine-serotonin vesicular transport defect  Autosomal recessive GTP cyclohydrolase I deficiency  Autosomal dominant GTPCH deficiency  Sepiapterin reductase deficiency  DNAJC12-deficient hyperphenylalaninemia  Pyridoxine-dependent epilepsy  PNPO deficiency  COQ6 deficiency | BRAIN ATROPHY:  Creatine transporter deficiency  Imerslund-Gräsbeck syndrome  Gaucher disease  Cerebrotendinous xanthomatosis  SLC39A14 deficiency  Molybdenum cofactor deficiency  Celia's encephalopathy  Folate receptor alpha deficiency  Pyruvate dehydrogenase complex deficiency  Zellweger spectrum disorders  Biotinidase deficiency  Arginase deficiency  Mitochondrial ornithine transporter deficiency  Gaucher disease  CLN2 disease  NAXE deficiency  Dihydrofolate reductase deficiency  Tyrosine hydroxylase deficiency  Aromatic L-amino acid decarboxylase deficiency  Sepiapterin reductase deficiency  DNAJC12-deficient hyperphenylalaninemia  COQ6 deficiency  SLC39A8 deficiency  Polymicrogyria, /pachygyria:  Zellweger spectrum disorders  Malonic aciduria  Heterotopias:  Zellweger spectrum disorders  Argininosuccinate lyase deficiency  Malonic aciduria  INTRACEREBRAL CALCIFICATIONS:  Dihydropteridine reductase deficiency  Folate receptor alpha deficiency  Dihydrofolate reductase deficiency  Hereditary folate malabsorption  Phenylketonuria | CEREBELLAR ATROPHY:  CAD trifunctional protein deficiency  Gaucher disease  Cerebrotendinous xanthomatosis  SLC39A14 deficiency  Molybdenum cofactor deficiency (*GPHN*)  COQ2 deficiency  COQ8A deficiency  Folate receptor alpha deficiency  Alpha-tocopherol transfer protein deficiency  dna:  Cerebrotendinous xanthomatosis  Thiamine pyrophosphokinase deficiency  Biotinidase deficiency  Argininosuccinate synthetase deficiency  Argininosuccinate lyase deficiency  Arginase deficiency  Alpha-mannosidosis  Dihydrofolate reductase deficiency  Recessive porphobilinogen deaminase deficiency  SLC39A8 deficiency  CEREBELLAR WHITE MATTER ABNORMALITIES:  Cerebrotendinous xanthomatosis  CEREBELLAR POLYMYCROGIRIA  Molybdenum cofactor deficiency (*GPHN*) | THIN CORPUS CALLOSUM  Hypoxanthine guanine phosphoribosyltransferase deficiency  Creatine transporter deficiency  Gaucher disease  Molybdenum cofactor deficiency  Aromatic L-amino acid decarboxylase deficiency  ABSENT/HYPOPLASIA CORPUS CALLOSUM  Glycine encephalopathy due to aminomethyltransferase deficiency  Pyruvate dehydrogenase complex deficiency  Zellweger spectrum disorders  Pyridoxine-dependent epilepsy | DELAYED MYELINATION:  Hypoxanthine guanine phosphoribosyltransferase deficiency  Creatine transporter deficiency  Methylmalonyl-CoA epimerase deficiency  Glutaric aciduria type 1  Molybdenum cofactor deficiency  X-linked adrenoleukodystrophy  Zellweger spectrum disorders  X-linked adrenoleukodystrophy  Alpha-mannosidosis  Dihydrofolate reductase deficiency  Hereditary folate malabsorption  Sepiapterin reductase deficiency  6-Pyruvoyl-tetrahydropterin synthase deficiency  cblX disease  LEUKODYSTROPHIES – WHITTE MATTER ABNORMALITIES:  Maple syrup urine disease  Phenylketonuria  X-linked adrenoleukodystrophy  Metachromatic leukodystrophy  Methylenetetrahydrofolate reductase deficiency  Argininosuccinate lyase deficiency  X-linked adrenoleukodystrophy  Alpha-mannosidosis  Metachromatic leukodystrophy  Folate receptor alpha deficiency  NAXE deficiency  Methylcobalamin synthesis defect - cblD variant 1  Tyrosine hydroxylase deficiency (periventricular)  Aromatic L-amino acid decarboxylase deficiency  Malonic aciduria  Recessive porphobilinogen deaminase deficiency  Classic galactosemia  Methylmalonic aciduria due to methylmalonyl-CoA mutase deficiency  Hypoxanthine guanine phosphoribosyltransferase deficiency  HYPOMYELINATION:  Folate receptor alpha deficiency  Aromatic L-amino acid decarboxylase deficiency | T2W HYPERINTENSITIES  Guanidinoacetate methyltransferase deficiency  Ornithine transcarbamylase deficiency  Ethylmalonic encephalopathy  Isovaleric acidemia  Methylmalonyl-CoA epimerase deficiency  Glutaric aciduria type 1  Beta-ketothiolase deficiency  Pyruvate dehydrogenase complex deficiency  Thiamine pyrophosphokinase deficiency  Biotin-thiamine-responsive basal ganglia disease  Mitochondrial thiamine pyrophosphate transporter deficiency  Argininosuccinate synthetase deficiency  Argininosuccinate lyase deficiency  Malonic aciduria  Wilson disease  Methylmalonic aciduria due to methylmalonyl-CoA mutase deficiency  T1W HYPERINTENSITIES  SLC39A14 deficiency  SLC30A10 deficiency  THALAMIC LESIONS  Glutathione synthetase deficiency  Recessive porphobilinogen deaminase deficiency  INFARCTION:  Propionic acidemia  IRON DEPOSITION:  Aceruloplasminemia | ↓ CREATINE PEAK ON MRS:  Creatine transporter deficiency  ↑LACTATE PEAK ON MRS:  Pyruvate dehydrogenase complex deficiency  Thiamine pyrophosphokinase deficiency  Biotin-thiamine-responsive basal ganglia disease  Mitochondrial thiamine pyrophosphate transporter deficiency  CORTICAL AND SUBCORTICAL EDEMA DURING DECOMPENSATION:  Ornithine transcarbamylase deficiency  Maple syrup urine disease  Argininosuccinate synthetase deficiency  STROKE:  Classic homocystinuria  Argininosuccinate lyase deficiency  Methionine synthase deficiency – cblG  Methylmalonic aciduria due to methylmalonyl-CoA mutase deficiency  CEREBRAL PEDUNCLES ABNORMALITIES  Cerebrotendinous xanthomatosis  INTERNAL CAPSULE/CORTICOSPINAL TRACT ABNORMALITIES  Cerebrotendinous xanthomatosis  Refsum disease  Glycine encephalopathy due to glycine decarboxylase deficiency  FACE OF THE PANDA SIGN:  Wilson disease  Subependymal cysts:  Zellweger spectrum disorders  ABSENT OLFATORY LOBES:  Zellweger spectrum disorders  INTRACRANIAL HEMORRAGHE  Menkes disease  SPINAL MYELOPATHY:  NAXE deficiency  Holocarboxylase synthetase deficiency  cblC disease |
