# Supplementary material for: A Proposed Diagnostic Algorithm for Inborn Errors of Metabolism Presenting With Movements Disorders
Source: Front Neurol. 2020 Nov 13;11:582160. doi: 10.3389/fneur.2020.582160 (PMC7691570; doi:10.3389/fneur.2020.582160)
Supplement: Supplementary file 6 [file Table_6.DOCX]

| **IEM group/Genes** | **MD phenotype in children** | **Other clinical features** | **Biochemical findings** | **MRI** | **Disease-specific treatment** | **Ref** |
| --- | --- | --- | --- | --- | --- | --- |
| **Disorders of nitrogen containing compounds** | | | | | | |
| **Disorders of purine and pyrimidine metabolism** | | | | | | |
| *CAD* | Early onset ataxia usually after seizure beginning | ID, S, epileptic encephalopathy, hypotonia, renal tubular acidosis | Anemia, acanthocytosis, ↑ammonia | Progressive BA | Uridine | Koch et al., 2017 |
| *PRPS1* | *PRPS1* superactivity: early onset ataxia  Arts syndrome: Early onset ataxia before age two years | *PRPS1* superactivity: variable ID, hypotonia, gout, SNHL  Arts syndrome: ID, congenital SNHL, hypotonia, optic atrophy, PN, frequent infections leading to early death | *PRPS1* superactivity: hyperuricemia, hyperuricosuria  Arts syndrome: Normal range serum uric acid, ↓urine hypoxanthine | *PRPS1* superactivity: Normal  Arts syndrome: Normal to decreased WM volume | *PRPS1* superactivity: Allopurinol, febuxostat, urine alkalization  Arts syndrome: SAM | de Brouwer et al., 2015  de Brouwer et al., 2018 |
| *PNP* | Ataxia (<30% of patients), tremor | ID, hypotonia, splenomegaly, failure to thrive, frequent infections, malignancies | Autoimmune hemolytic anemia, thrombocytopenia, autoimmune neutropenia, ↓serum and urine uric acid, ↑urine inosine, 2'deoxyinosine, guanosine, and 2'deoxyguanosine | Cerebral vasculitis | HSCT | Fekrvand et al., 2019 |
| *ADSL* | Gait ataxia, stereotypies (hand movements, repetitive manipulation of toys, grimacing, clapping hands, rubbing feet, inappropriate laughter, head and trunk rocking, and stereotyped sounds) and epileptic myoclonus | Broad spectrum: neonatal encephalopathy to ID, hypotonia, autistic features, self-injurious behavior, failure to thrive | ↑S-Ado in serum, urine, and CSF, ↑SAICAr, ↓S-Ado:SAICAr ratio | BA, CeA, hypomyelination and lissencephaly | D-ribose, uridine and SAM | Jurecka et al., 2015 |
| *HPRT1* | Severe action dystonia, choreoathetosis, ballismus and opisthotonos usually between 12 and 18 mo. (range 1 mo. to 6 y), similar to athetoid CP | ID, hypotonia, self-injurious behavior (biting of the lips or fingers), gout, nephrolithiasis, failure to thrive, short stature, vomiting | Megaloblastic anemia, hyperuricemia, hyperuricosuria | Diffuse thinning of the CC, decreased WM volume with enlarged extra-axial cerebrospinal fluid space, and DM | Allopurinol | Nyhan et al., 2014 |
| **Disorders of nucleotide metabolism** | | | | | | |
| *TREX1, RNASEH2B, RNASEH2A, RNASEH2C, SAMHD1, ADAR, IFIH1* | Dystonia | IUGR, microcephaly, abnormal ocular movement, chilblain lesions, hypotonia, ID, cognitive regression, spasticity, S rarely hepatomegaly, splenomegaly, lupus like lesions, glaucoma, cardiomyopathy | Thrombocytopenia, ↑CSF pterins, upregulation of alpha-interferon-stimulated genes, autoantibodies, hypocomplementemia, hypergammaglobulinemia | BG calcification, BA | None | Crow 2016 |
| **Disorders of creatine metabolism** | | | | | | |
| *GAMT* | Dystonia and choreoathetosis | ID, hypotonia, S, hyperreflexia | ↓CSF creatine, ↓CSF creatinine, ↓ creatine excretion | BG hyperintensities | Creatine and ornithine supplementation, arginine restriction | Dhar et al., 2009 |
| *SLC6A8* | Early onset dystonia (3 month of age) worsened by infections, oromandibular dystonia. Choreiform movements (33 month of age) | Failure to thrive, microcephaly, ID, hypotonia, spasticity, S, behavioral abnormalities | ↑urinary and plasma creatine, ↑urinary creatine-to-creatinine ratio | BA, DM, TCC, ↓creatine peak on MRS | Creatine, arginine and glycine supplementation | Anselm et al., 2006 |
| **Disorders of glutathione metabolism** | | | | | | |
| *GCLC* | Adult-onset ataxia started in the thirties | ID, Myopathy, PN | Neonatal hemolytic anemia, ↓Glutathione | Normal | None | Almusafri et al., 2017 |
| *GSS* | Adolescent-onset ataxia and intention tremor | ID, psychiatric abnormalities | Hemolytic anemia, episodic neutropenia, pyroglutamic acidemia and aciduria | Visual cortex and thalamus lesions | N-acetylcysteine, vitamins E and C | Yapicioğlu et al., 2004 |
| **Disorders of ammonia detoxification** | | | | | | |
| *OTC* | Vomiting, ataxia and altered mental status can be a form of presentation. Intermittent choreiform movements of the arms can also be noted during decompensations. | Failure to thrive, vomiting, ID, S | Respiratory alkalosis, ↑ammonia, glutamine, asparagine, ↓ citrulline, arginine, orotic aciduria | During decompensation: cortical and subcortical edema. BG T2W hyperintensity with thalamic sparing. | Protein restriction, ammonia scavengers, citrulline, liver transplant | Myers et al., 1996  Keegan et al., 2003 |
| *ASS1* | Brief episodes of ataxia triggered by minor febrile illnesses | Failure to thrive, hepatomegaly, vomiting, ID, S | ↑citrulline, glutamine, ↓arginine, episodic hyperammonemia, orotic aciduria | Mild CeA, cortical subcortical edema (decompensation), BG T2W hyperintensities | Protein restriction, ammonia scavengers, arginine, liver transplant | Saini et al., 2018 |
| *ASL* | Ataxia (17% of patients) was first noticed at a median age of 8.5 years (range 1–12) with two main age groups at first observation, early around the age of 1 year (n = 2) or later as teenagers (n = 7). Three patients had dyskinesia and tremor | Failure to thrive, hepatomegaly, vomiting, trichorrhexis nodosa, ID, S | ↑transaminases, citrulline, glutamine, episodic hyperammonemia, orotic aciduria, argininosuccinicaciduria | Parenchymal infarcts, WM abnormalities, nodular heterotopia, CeA, BA, thalamic atrophy, hyperintensity of caudate head and posterior putamen, isosignal between pallidi and putamen | Protein restriction, ammonia scavengers, arginine, liver transplant | Baruteau et al. 2017 |
| *ARG1* | Ataxia (12,5%) and choreic movement and tremor (6%) have been described. | Failure to thrive, vomiting, ID, S | ↑ammonia, arginine, Diaminoaciduria (arginuria, lysinuria, cystinuria, ornithinuria), orotic aciduria, pyrimidinuria, ↑CSF amino acids (arginine, ornithine, aspartate, threonine, glycine, and methionine) | BA, CeA | Protein restriction, ammonia scavengers | Rocha et al., 2012 |
| *SLC25A15* | Ataxia and myoclonus have been described. | Failure to thrive, hepatomegaly, episodic vomiting, ID, hypotonia, S, spasticity, hyperreflexia, acute encephalopathy | ↑ammonia, ornithine, homocitrullinuria | BA | Ornithine-citrulline supplementation, protein-restricted diet and sodium benzoate | Tunali et al., 2014 |
| **Disorders of amino acid transport** | | | | | | |
| *SLC6A19* | Intermittent cerebellar ataxia, only in a few cases, the onset is before 5 years of age. | ID, S, hypertonia, psychosis, light-sensitive dermatitis – pellagra-like lesions, short stature | Neutral hyperaminoaciduria | BA, abnormal CC, DM | High-protein diet, nicotinamide, tryptophan | Cheon et al., 2010 |
| **Disorders of monoamine metabolism** | | | | | | |
| *TH* | There have been described three phenotypes: (1) TH-deficient dopa-responsive dystonia (the mild form of TH deficiency) age onset: 12 months to 12 years of age, initial symptoms are typically lower-limb dystonia and/or difficulty in walking (2) TH-deficient infantile HRS (hypokinesia, rigidity of extremities, and/or tremor) with motor delay (the severe form), age at onset: 3 to 12 months, and (3) TH-deficient progressive infantile encephalopathy (the very severe form), age at onset: 3 to 6 months: severe hypokinesia and limb hypertonia. | Oculogyric crisis, ptosis, truncal hypotonia | ↓CSF HVA, normal CSF 5-HIAA | Normal to BA, CeA, periventricular WM changes and symmetric high signal abnormalities in the superior cerebellar peduncles and dorsal pons | L-dopa | Furukawa et al., 2018 |
| *DDC* | MD most often described are oculogyric crises (77%), dystonia (53%), and hypokinesia (34%). Dyskinesia (e.g. hyperkinesia, chorea, athetosis), myoclonus and tremor have also been described. Status dystonicus can occur in AADCD patients. | Oculogyric crisis, ptosis, ID, truncal hypotonia, episodic hypothermia | ↓CSF HVA, 5-HIAA | Normal to BA, WM abnormalities, thin CC, hypomyelination, leukodystrophy-like pattern | Dopamine agonists, MAO inhibitors, pyridoxine | Wassenberg et al., 2017 |
| *SLC6A3* | Classical DTDS: nonspecific findings following by hyperkinetic MD (with features of chorea, dystonia, ballismus, orolingual dyskinesia). Atypical DTDS: Normal psychomotor development in infancy and early childhood is followed by later onset manifestations of parkinsonism-dystonia with tremor, progressive bradykinesia, variable tone, and dystonic posturing. | Ocular flutter, oculogyric crisis, ID, truncal hypotonia | ↑CSF HVA, normal CSF 5-HIAA | Normal | Poor response to L-dopa. Dopamine agonists | Kurian, 2016 |
| *SLC18A2* | Gait dystonia, HRS, and oculogyric crises have been described. | Oculogyric crisis, ptosis, truncal hypotonia, ID, hyperreflexia, temperature instability | Normal CSF neurotransmitters, ↓ urine HVA and 5-HIAA | Normal | Poor response to L-dopa. Dopamine agonists | Rilstone et al., 2013 |
| **Disorders of phenylalanine and tetrahydrobiopterin metabolism** | | | | | | |
| *PAH* | Fine action tremor (16%). Dystonia is observed at later  ages | Microcephaly, cataracts, blonde hair, ID, S, autistic features, psychosis | ↑plasma phenylalanine, phenylpyruvic acidemia | Brain calcifications, WM abnormalities | Phenylalanine restricted diet, sapropterin, pegvaliase | Yalaz et al., 2006 |
| *GCH1* | Childhood-onset dystonia and a dramatic and sustained response to low doses of oral administration of L-dopa. This disorder typically presents with gait disturbance caused by foot dystonia, later development of HRS signs. Occasionally, initial symptoms are arm dystonia, postural tremor of the hand, or slowness of movements. In general, gradual  progression to generalized dystonia is observed. | Abnormal ocular movement, ID, truncal hypotonia, S, episodic hyperthermia, torticollis, pes cavus | ↑plasma phenylalanine, ↓CSF HVA and normal or ↓ 5-HIAA and ↓CSF biopterin and neopterin | Normal | L-dopa, BH4 | Furukawa, 2019 |
| *PTS* | Short dystonic episodes with opisthotonic head posturing and limb rigidity can be observed at 6 weeks of age. Other extrapyramidal signs include characteristic truncal hypotonia, increased limb tone, postural instability, hypokinesia, choreatic or dystonic limb movements. | Microcephaly, oculogyric crisis, hypersalivation, ID, truncal hypotonia, S, episodic hyperthermia | ↑plasma phenylalanine and CSF neopterin, ↓CSF HVA and 5-HIAA, | DM | L-dopa, BH4 | Dudesek et al., 2001 |
| *SPR* | Dystonia and oculogyric crisis are present >65% of patients. Other common features include HRS signs (tremor, bradykinesia, masked facies, rigidity), choreoathetosis and ataxia | Failure to thrive, microcephaly, oculogyric crisis, oculomotor apraxia, ID, S, truncal hypotonia | ↓CSF HVA and 5-HIAA and urinary HVA, 5-HIAA and VMA, ↑CSF sepiapterin, biopterin and dihydrobiopterin | Normal to DM or BA | L-dopa, BH4 | Friedman, 2015 |
| *QDPR* | Tremor, dystonia, choreoathetosis | Microcephaly, ID, truncal hypotonia, S, episodic hyperthermia | ↑plasma phenylalanine, ↓CSF HVA and 5-HIAA | Intracerebral calcifications | L-dopa, BH4 | Opladen et al., 2020 |
| *DNAJC12* | Progressive MD with prominent dystonia | Nystagmus, oculogyric crisis, hypotonia, ID, autistic features | ↑plasma phenylalanine, ↓CSF dopa and serotonin metabolites | Normal to BA | L-dopa, BH4 | Anikster et al., 2017 |
| **Disorders of sulfur amino acid and sulfide metabolism** | | | | | | |
| *MAT1A* | 12% of reported patients show dystonia. | ID (rare), hyperreflexia, halitosis | ↑plasma methionine | Demyelination | None | Chamberlin et al., 1996 |
| *CBS, MTHFR, MTR (cblG), MTRR (cblE) and MMADHC (clbC and cblD)* | *CBS*: dystonia  *MTHFR and MTRR:* ataxia  *MMADHC*: ataxia and dystonia | CBS: Ectopia lentis, glaucoma, steatosis, pancreatitis, skeletal abnormalities,  MTHFR: microcephaly  MTR and MTRR: failure to thrive, nystagmus, hypotonia  All of them: S, ID  Atypical hemolytic uremic syndrome | All of them: Homocystinuria  CBS: methioninuria  MTHFR: N or ↓ plasma methionine, homocystinemia  MTR and MTRR: megaloblastic anemia, ↓plasma methionine  *MMADHC:* ↑ methionine | Stroke (thromboembolism), *MMADHC*: leukodystrophy ranging from isolated periventricular WM hyperintensities  to diffuse WM loss | Methionine or protein restricted diet, cystine supplemented diet for pyridoxine non-responders, pyridoxine supplementation for pyridoxine responders, betaine, folic acid, hydroxycobalamin, carnitine and vitamin C | Sacharow S et al., 2017  Sloan et al., 2018 |
| *SUOX* | MD characterized by dystonia and choreoathetosis, ataxia, (rarely) acute hemiplegia due to metabolic stroke and neonatal hyperekplexia | ID, S, hypotonia/hypertonia, eczema, ectopia lentis, delayed teething | ↑urine sulfite, ↓urine sulfate | Cystic leukomalacia, ventricular dilatation, diffuse ulegyria. CeA, thin CC | None | Bindu et al., 2017 |
| *ETHE1* | Very slowly motor deterioration, ataxia and dystonic posturing of the jaw and tongue have been described. | Failure to thrive, retinal tortuous vessels, chronic diarrhea, petechiae, acrocyanosis, ID, hypotonia, S | ↑plasma lactate, ethylmalonic and methylsuccinic aciduria, ↑urine isobutyryl glycine, 2-methylbutyryl glycine and thiosulphate | BG T2W hyperintensities | N-acetylcysteine, metronidazole, liver transplant | Grosso et al., 2002 |
| **Disorders of branched-chain amino acid metabolism** | | | | | | |
| *BCKDHA*  *BCKDHB*  *DBT* | During metabolic crisis, patients present ataxia (age onset 18 month of age) usually triggered by fever. The ataxia could resolve spontaneously without specific treatment. Dystonic posturing can also be observed during the decompensations. | Pancreatitis, vomiting, S, coma, ID (untreated), hypotonia, hallucinations, Maple syrup urine odor | Ketosis, hypoglycemia, ↑leucine, isoleucine, valine, ↑ ketoaciduria: alpha-keto isocaproate, alpha-keto-beta methylisovalerate, alpha-keto isovalerate | Brain edema, WM and diffusion abnormalities | Low BCAA (leucine restricted) diet, valine/isoleucine supplementation | Pode-Shakked et al., 2020 |
| *DLD* | Recurrent episodes of encephalopathy with ataxia and intention tremor. Dystonia has also been described. | Microcephaly, hypertrophic cardiomyopathy, hepatomegaly (rare), vomiting, episodic encephalopathy, ID (frequent), hypotonia, S | Metabolic and lactic acidosis, hypoglycemia, ↑transaminases, pyruvate, leucine, isoleucine and valine, ↑alpha ketoglutarate | BG T2W hyperintensities | None | Hong et al., 2003 |
| *IVD* | Ataxia has been described in few cases | Vomiting, aversion to protein, ID, S, coma, sweaty feet odor | Metabolic acidosis, ketoacidosis, thrombocytopenia, leukopenia, pancytopenia, - isovalericacidemia, isovalericaciduria and isovalerylglycinuria | Normal to brain hemorrhage, BG T2W hyperintensities | Glycine, carnitine | Sogut et al., 2004 |
| *AUH* | Adult onset cerebellar ataxia | Failure to thrive, optic atrophy, ID, hyperreflexia | Metabolic acidosis, ↑urine 3-methylglutaconic acid and hydroxyisovaleric acid | BA, BG atrophy, leukoencephalopathy | None | Wortmann et al., 2010 |
| *ECHS1* | There have been described 3 phenotypes. The mildest phenotype present isolated paroxysmal dystonia (52%) or choreoathetosis/ataxia (20%) that may be exacerbated by illness or exertion | Nystagmus, SNHL (rare), ID (variable), spasticity | ↑plasma and CSF lactate, ↑urine S-(2-carboxypropyl)cysteine and 2-methyl-2,3-dihydroxybutyrate | BG T2W hyperintensities | Valine restriction | Ganetzky et al., 2019 |
| *HIBCH* | Short-lasting, reversible dystonic gait disturbances during febrile infections, and nonprogressive ataxia have been described | Nystagmus, strabismus, vomiting, ID, S, | ↑plasma lactate, hydroxy-C4-carnitine and ↑urine cysteine and cysteamine conjugates of methacrylic acid | BG T2W hyperintensities | None | Schottmann et al., 2013 |
| *HSD17B10* | Generalized rigidity with some dystonic posturing at 15 years of age. Tremor can appear later in the disease course. | SNHL, nystagmus, optic atrophy, retinal degeneration, hypertrophic cardiomyopathy, ID, S, spasticity | Metabolic and lactic acidosis, ↑urine 2-methyl-3 hydroxybutyrate and tiglylglycine | BA, BG T2W hyperintensities | None | Olpin et al., 2002 |
| *PCCA*  *PCCB* | BG infarction which may result in dystonia and choreoathetosis | Failure to thrive, cardiomyopathy, hepatomegaly, pancreatitis, vomiting, coma/acute encephalopathy, hypotonia, S, ID | Pancytopenia, metabolic acidosis, ↑plasma lactate, ammonia, glycine, hypoglycemia, ↓serum carnitine, ↑urine glycine, propionate,  3-hydroxypropionic acid and 3-methylcitric acid | BG infarction | Protein-restricted diet and carnitine supplementation | Jurecki et al., 2019 |
| *MCEE* | Dystonia has been described in some patients | Failure to thrive, | Metabolic acidosis, methylmalonic aciduria, ↑plasma propionylcarnitine, ketonuria | Acute metabolic stroke | Hydroxycobalamin | Bikker et al., 2006 |
| *MMUT* | Ataxia, dystonia and choreoathetosis have been reported | Failure to thrive, cardiomyopathy, hepatomegaly, pancreatitis, vomiting, interstitial nephritis, hypotonia, coma | Metabolic ketoacidosis, leukopenia, thrombocytopenia, ↑ammonia, glycine, ↓ plasma free and total carnitine, methymalonicaciduria | Globus pallidus T2W hyperintensities, DM, BG ischemic stroke | Low protein diet, carnitine, hydroxycobalamin | Shevell et al., 1993 |
| *ACSF3* | Dystonia has been described in some patients | Failure to thrive, ID, S, coma, microcephaly, hypotonia | Ketoacidosis, hypoglycemia, ↑transaminases, plasma methylmalonic and malonic acid, and malonic and methylmalonic aciduria | WM hyperintensities | None | Sloan et al., 2013 |
| *MLYCD* | Dystonia has been described in some patients | Short stature, cardiomyopathy, vomiting, ID, S | Metabolic acidosis, hypoglycemia, ↑plasma lactate, | Periventricular WM and BG hyperintensities, pachygyria, nodular heterotopia | LCT restriction, MCT and carnitine supplementation | Chapel-Crespo et al., 2019 |
| **Disorders of lysine metabolism** | | | | | | |
| *GCDH* | Progressive complex MD including dystonia and choreoathetosis. Dystonia is a significant sequela for individuals with BG injury. Those who have insidious onset generally have less severe MD. | Failure to thrive, macrocephaly, hepatomegaly, hypotonia, S, opisthotonos, | Hypoglycemia, ketonemia, ketonuria, metabolic acidosis, glutaricaciduria | DM, striatal necrosis | Carnitine supplements, lysine-restricted/arginine- rich diet | Larson et al., 2019 |
| **Disorders of proline and ornithine metabolism** | | | | | | |
| *ALDH4A1* | Dystonia and choreoathetosis | ID, S | Hyperprolinemia, aminoaciduria, prolinuria, hydroxyprolinuria, glycinuria, ↑ plasma and urine P5C | BA | None | Mastrangelo et al., 2012 |
| **Disorders of β- and γ-amino acids** | | | | | | |
| *UPB1* | Dystonia and ataxia have been reported. | Hypotonia, ID, S, variable anal atresia, bifid scrotum, bifid phallus, bladder exstrophy and scoliosis | ↑urine dihydropyrimidines, ↑plasma, urine and CSF N-carbamyl-beta-alanine, N-carbamyl-beta-aminoisobutyric acid, dihydrouracil and dihydrothymine | DM | None | Fang et al., 2019 |
| *ABAT* | Early onset nonprogressive ataxia (3 years of age) , persistent chorea and adult onset paroxysmal dyskinesia. Drowsiness has been reported as a diagnostic clue. | ID, S, hyperreflexia, mild dysmorphic features | ↑plasma, urine and CSF gamma-aminobutyric acid, ↑beta-alanine, ↑growth hormone | Leukodystrophy, agenesis CC, cerebellar hypoplasia, posterior fossa cysts | None | Morales-Briceño et al., 2019 |
| *ALDH5A1* | Choreoathetosis, dystonia, myoclonus, nonprogressive ataxia (45%), stereotypes and paroxysmal diskinesias/dystonia have been reported. | Variable ID, hypotonia, S, hyporeflexia, microcephaly | ↑plasma, urine and CSF GABA, ↑plasma, urine and CSF GHB | Globus pallidi, dentate and subthalami T2W hyperintensities | Vigabatrin | Pearl P et al., 2003  Lyn et al. 2015 |
| **Disorders of glutamate metabolism** | | | | | | |
| *SLC1A3* | Childhood onset episodic ataxia and intentional tremor. Episodes may last several hours and be triggered by fever, illness, feeding, stress, exercise, cold. Adult onset ataxia has also been described. | Hemiplegic episodes, S, migraine, interictal nystagmus | None | Normal to CeA | Acetazolamide | Iwama et al., 2018 |
| **Disorders of serine metabolism** | | | | | | |
| *PHGDH* | Adult onset mild cerebellar ataxia | Failure to thrive, microcephaly, congenital cataracts, nystagmus, ID, S, spasticity, adult onset PN | Megaloblastic anemia, thrombocytopenia, ↓ plasma (fasting) and CSF serine, N or ↓ glycine, ↓ CSF glycine | DM | Serine/glycine supplementation | Merenet et al., 2012 |
| **Disorders of glycine metabolism** | | | | | | |
| *GLDC, AMT* | Classical phenotype: myoclonic jerks in the first week of life (neonatal period).  Milder phenotype: late onset-chorea, intermittent ataxia triggered by febrile illness | Encephalopathy, S, hiccups, ID, early death | ↑ plasma, urine and CSF glycine, ↑CSF/plasma glycine ratio | Restricted diffusion in the posterior limbs of the internal capsules, dorsal midbrain and pons | Benzoate, NMDA receptor antagonist | Coughlin II et al., 2017, Kanekar et al., 2013 |
| *SLC6A5* | Neonatal hypertonia, muscle stiffness, exaggerate startle response, nocturnal myoclonus | Neonatal apneic episodes | None | Normal to Absent CC | Clonazepam | Bakker et al., 2009 |
| **Disorder of asparagine metabolism** | | | | | | |
| *ASNS* | Jitteriness (tremulous movement), excessive startle reflex, mimicking hyperekplexia. | Failure to thrive, progressive severe microcephaly, spastic tetraplegia, S | ↓ asparagine | BA, cerebellar hypoplasia, thin CC, DM, cortical dysplasia | None | Ruzzo et al., 2013 |
| **Disorders of tyrosine metabolism** |  |  |  |  |  |  |
| *HPD* | Acute ataxia and drowsiness | ID, seizure | Tyrosinemia, 4-hydroxyphenylpyruvic, 4-hydroxyphenyllactic and 4-hydroxyphenylacetic aciduria, T-cell alteration | None |  | D´Eufemia et al., 2009 |
| **DISORDERS OF VITAMINS, COFACTORS, METALS AND MINERALS** | | | | | | |
| **Disorders of cobalamin metabolism** | | | | | | |
| *AMN, CUBN* | Ataxia, onset before 5 years of age | Failure to thrive, vomiting, PN | Megaloblastic anemia, mild proteinuria, ↓ vitamin B12 | Normal to BA | Hydroxocobalamin | Huemer et al., 2017 |
| MMACHC | Few patients (<10%) show later onset tremor and ataxia | Failure to thrive, microcephaly, nystagmus, retinitis pigmentosa, hemolytic-uremic syndrome, thromboembolism, ID, hypotonia, S | Megaloblastic anemia, thrombocytopenia, neutropenia,↑homocysteine, homocystinuria, methylmalonic acidemia and aciduria, ↓methionine, uremia, hematuria, proteinuria | Normal to spinal cord involvement | Hydroxocobalamin, betaine | Huemer et al., 2017 |
| *C2orf25* | Dystonia, spastic ataxia | Nystagmus, hypotonia, ID | Megaloblastic anemia, ↑homocysteine, homocystinuria, methylmalonic acidemia and aciduria, N or ↓ cobalamin, ↓methionine | Normal to BA or CeA | Hydroxocobalamin, betaine | Huemer et al., 2017 |
| *MTR* | Gait ataxia (10-25%) | Failure to thrive, nystagmus, ID, hypotonia, S | Megaloblastic anemia, ↑homocysteine, ↓methionine, homocystinuria | Normal to BA | Hydroxocobalamin, betaine | Huemer et al., 2017 |
| *HCFC1* | Choreoathetosis (35%) | Failure to thrive, microcephaly, ID, S, hypotonia | ↑homocysteine, homocystinuria, methylmalonic acidemia and aciduria | DM |  | Yu et al. 2013 |
| **Disorders of folate metabolism** | | | | | | |
| *SLC46A1* | Ataxia, choreoathetosis, dyskinesias | Failure to thrive, oral ulcers, diarrhea, ID, hypotonia, PN, S, recurrent infections, | Megaloblastic anemia, thrombocytopenia, leukopenia, neutropenia, ↓serum and CSF 5-MHTF, ↓methionine and ↑urinary formiminoglutamic acid | Demyelination, BG calcification | Folinic acid | Pope et al., 2019 |
| *FOLR1* | Typ  ical clinical signs at presentation are tremor, ataxia,  54  Typ-  ical clinical signs at presentation are tremor, ataxia,  Intention tremor, progressive ataxia and epileptic myoclonus are typically found at diagnosis (median onset 2 years, range 6 mo. to 4,5 y). Later, they develop choreoathetosis. | ID, hypotonia, congenital microcephaly, PN, S, sensory stimulus-sensitive drop attacks | ↓CSF 5-MTHF | Hypomyelination, leukoencephalopathy, BA, CeA, BG calcifications | Folinic acid 0,5 – 7 mg/kg/d | Pope et al., 2019 |
| *MTHFR* | Gait ataxia | Variable clinical presentation: neonatal encephalopathy: S, hydrocephalus or later-onset: cognitive decline, paresthesia | Megaloblastic anemia, pancytopenia, ↑homocysteine, N or ↓methionine, homocystinuria | WM abnormalities | Betaine, folinic acid or 5- methyltetrahydrofolate | Pope et al., 2019 |
| *DHFR* | Ataxia and eyelid myoclonus | Acquired microcephaly, hepatomegaly, ID, hypotonia, S | Megaloblastic anemia, pancytopenia, thrombocytopenia, hypersegmented neutrophils, ↓CSF tetrahydrofolate, BH4, and 5-MHTF | BA, CeA, DM, brain calcification | Folinic acid | Pope et al., 2019 |
| **Disorders of biotin metabolism** | | | | | | |
| BTD | Reversible progressive or intermittent mild ataxia, usually not the initial manifestation | SNHL, optic atrophy, hepatomegaly, splenomegaly, seborrheic dermatitis, skin rash, alopecia, S, hypotonia, ID | ↑beta-hydroxyisovalerate, lactate, beta-methylcrotonylglycine, beta-hydroxypropionate and methyl citrate, biotinidase deficiency, mild hyperammonemia | Diffuse BA, CeA | Biotin | Wolf et al., 2019 |
| HLCS | Truncal ataxia has been reported in some patients | Alopecia, skin rash, vomiting, hypotonia, D, ID, coma | Thrombocytopenia, metabolic acidosis, ↑ammonia, ↑beta-hydroxyisovalerate, beta-methylcrotonylglycine, beta-hydroxypropionate, methylcitrate, lactate, tiglylglycine | Normal or myelopathy | Biotin | Aoki et al., 1999 |
| **Disorders of thiamine metabolism** | | | | | | |
| *SLC19A3* | Dystonia was the second most common sign after encephalopathy. The BFMDS questionnaire was administered to 34 SLC19A3 patients with dystonia (9.8 6 1.8 points [mean 6 SEM]; range, 0–30). Higher BFMDS scores were identified in patients who had a previous history of developmental delay and in patients with disease onset before 6 months of age. A positive, and almost significant, correlation was observed between the BFMDS scores and the time from disease onset to thiamine initiation | Nystagmus, ophthalmoplegia, ptosis, episodic encephalopathy, S, truncal hypotonia, spasticity | Metabolic and lactic acidosis, ↓CSF free thiamine | BG hyperintensities, ↑lactate on MRS | Thiamine, biotin | Ortigoza-Escobar et al., 2017 |
| *TPK1* | Ataxia, dystonia | Hypotonia, episodic encephalopathy, ID, S, spasticity | Metabolic and lactic acidosis, ↓thiamine pyrophosphate, ↑urine alpha-ketoglutaric acid | BG and dentate nuclei hyperintensities, ↑lactate on MRS | Thiamine | Ortigoza-Escobar et al., 2017 |
| *SLC25A19* | All patient has mild to severe foot dystonia. | Episodic encephalopathy, PN | ↑CSF lactate | Caudate and putamen cavitation, ↑lactate on MRS | Thiamine | Ortigoza-Escobar et al., 2017 |
| **Disorders of NAD metabolism** | | | | | | |
| *NADK2* | Dystonia and choreoathetosis have been described. | IUGR, failure to thrive, microcephaly, nystagmus, optic atrophy, ophthalmoplegia, encephalopathy, S, spasticity | ↑lactate, serum lysine, pipecolic acid, ↓ plasma free carnitine, abnormal OA | Leukodystrophy, CM, CeA, hypoplastic CC | Lysine restriction | Houten et el., 2014 |
| *NAXE* | Intermittent or progressive ataxia, head tremor, mild chorea, fine myoclonus and cervical distonia. | Nystagmus, strabismus, hypotonia, encephalopathy, coma, cognitive regression, S, spasticity | ↑serum and CSF lactate, | BA, leukoencephalopathy, spinal myelopathy | Niacin | Trinh et al., 2019 |
| **Disorders of pantothenate metabolism** | | | | | | |
| *PANK2* | Classic form: ataxia,  dystonia, chorea,  parkinsonism, cognitive  decline, abnormal eye  movements. Atypical form:  speech defects, dystonia,  tremor, psychiatric  symptoms, cognitive  impairment  lassic form: ataxia,  dystonia, chorea,  parkinsonism, cognitive  decline, abnormal eye  movements. Atypical form:  speech defects, dystonia,  tremor, psychiatric  symptoms, cognitive  impairment  lassic form: ataxia,  dystonia, chorea,  parkinsonism, cognitive  decline, abnormal eye  movements. Atypical form:  speech defects, dystonia,  tremor, psychiatric  symptoms, cognitive  impairment  lassic form: ataxia,  dystonia, chorea,  parkinsonism, cognitive  decline, abnormal eye  movements. Atypical form:  speech defects, dystonia,  tremor, psychiatric  symptoms, cognitive  impairment  Choreoathetosis, dystonia, including severe jaw-opening dystonia, HRS. Dystonia is always present and usually an early manifestation. Cranial dystonia and limb dystonia are frequent and may lead, respectively, to recurrent trauma to the tongue and to atraumatic long bone fracture from the combination of extreme bone stress and osteopenia. The resulting pain and distress can contribute to development of status dystonicus in a cycle that can be difficult to break. | Pigmentary retinopathy, optic atrophy, ID, spasticity, psychiatric abnormalities | None | Eye of the tiger sign, BA | None | Gregory et al., 2002 |
| *COASY* | Early onset ataxia signs can be observed as early as 24 months of age (hypotonia, broad based, ataxic gait, and ataxic hand movements). _Oromandibular_ dystonia, spastic-dystonia paresis and parkinsonian features have been described. | Cognitive regression, spasticity, pes cavus, PN | None | BG T2W abnormalities, thin CC, iron accumulation | None | Evers et al., 2017 |
| **Disorder of pyridoxine metabolism** | | | | | | |
| *ALDH7A1* | Myoclonus jerks | S, hypotonia, ID | ↑serum and CSF pipecolic acid and alpha-aminoadipic semialdehyde | Normal, CC abnormalities | Pyridoxine and arginine supplementation, lysine restriction | Mills et al., 2006 |
| *PNPO* | Neonatal mixed multifocal myoclonic tonic seizures | Failure to thrive, microcephaly, eye movement abnormalities, ID, S, truncal hypotonia | Metabolic acidosis, anemia, hypoglycemia, ↑lactate, CSF taurine, histidine and arginine, N or ↑plasma and CSF glycine and threonine, ↓ plasma arginine, CSF HVA, 5-HIAA, CSF PLP | Normal, WM edema | Pyridoxal phosphate | Plecko et al., 2014 |
| **Disorder of vitamin E metabolism** | | | | | | |
| *TTPA* | Late childhood or early teens between ages five and 15 years (age range: 2 to 37 years of age) progressive ataxia and clumsiness of the hand. Head tremor in 40% of cases. | Areflexia, proprioception loss, xanthelasmata, tendon xanthomas | ↓blood vitamin E, ↑serum cholesterol, triglycerides and beta-lipoprotein | Normal, CeA | Vitamin E | Schuelke et al., 2015 |
| **Disorders of molybdenum metabolism** | | | | | | |
| *MOCS1*  *MOCS2*  *GPHN* | Intractable epileptic myoclonus jerks | *MOCS1, MOCS2:* Failure to thrive, macro/microcephaly, dislocated lens, nystagmus, S, opisthotonos, spasticity  *GPHN*: hypotonia, ID, S | All defects: Hypouricemia, ↓sulfite oxidase activity  *MOCS1, MOCS2:* ↑urine xanthine, hypoxanthine, S-sulfocysteine and taurine,  *GPHN*: ↑urine S-sulfocysteine and taurine, | BA, thin CC, DM  *GPHN*: CeA, pontine hypoplasia, cerebellar polymicrogyria, WM abnormalities | Cyclic pyranopterin monophosphate (*MOCS1*) | Atwal et al., 2016 |
| **Disorders of copper metabolism** | | | | | | |
| *ATP7B* | Neurologic involvement follows two general patterns: movement disorders or rigid  dystonia.  • Movement disorders tend to occur earlier and include tremors, poor coordination, loss of fine-motor  control, micrographia (abnormally small, cramped handwriting), chorea, and/or choreoathetosis.  • Spastic dystonia disorders manifest as mask-like facies, rigidity, and gait disturbance | Kayser-Fleischer ring, hepatomegaly, cirrhosis, renal tubular dysfunction, osteoporosis, chondrocalcinosis, PN | Hemolytic anemia, hypoparathyroidism, ↓serum ceruloplasmin, ↑urinary copper, proteinuria, aminoaciduria, glucosuria, hypercalciuria, hyperphosphaturia | Face of the Panda sign | Chelation with D-penicillamine, trientine, or tetrathiomolybdate, Zinc acetate/sulphate | Weiss et al., 2017 |
| *ATP7A* | Ataxia, tremor, and head bobbing are characteristic neurologic findings in mild Menkes disease. | IUGR, microcephaly, osteoporosis, hair abnormalities, ID, hypotonia, S, hypothermia | ↓serum copper and ceruloplasmin | Intracranial hemorrhage | Copper histidine or copper chloride | Kaler et al., 2016 |
| **Disorders of iron metabolism** | | | | | | |
| *FTL* | Mean age of onset: 39.4 years (SD = 13.3, range 13-63), beginning with chorea in 50%, focal lower limb dystonia in 42.5% and parkinsonism in 7.5%. The majority reported a family history of a movement disorder often misdiagnosed as Huntington's disease. The disease progressed relentlessly, becoming generalized over a 5-10 year period, eventually leading to aphonia, dysphagia and severe motor disability with subcortical/frontal cognitive dysfunction as a late feature. A characteristic action-specific facial dystonia was common (65%), and in 63% there was asymmetry throughout the disease course. | Spasticity, hyperreflexia, | ↓serum ferritin | BG cavitation, brain iron accumulation | None | Chinnery et al., 2007 |
| *CP* | Ataxia, chorea. Onset between age 30-50 years | Retinal degeneration, diabetes mellitus | Mild anemia, ↓ or absent serum ceruloplasmin, ↓ iron, ↑ ferritin | Iron deposition in the basal ganglia and in the red and dentate nuclei | Iron chelation | Okamoto et al., 1996 |
| **Disorders of manganese metabolism** | | | | | | |
| *SLC30A10* | Although most cases show pure four-limb dystonia leading to a characteristic high stepping gait (a ‘‘cock-walk’’ gait) and fine motor impairment sometimes accompanied by dysarthria, fine tremor, and bradykinesia, one affected individual has pure spastic paraparesis without extrapyramidal dysfunction | Hepatomegaly, cirrhosis, PN | Polycythemia, ↑blood manganese, ↑unconjugated bilirubin, ↑transaminases, ↑erythropoietin, ↓iron, ↓ferritin, ↑TIBC | BG T1W hyperintensities | Chelation with CaNa2EDTA and iron supplementation | Tuschl et al., 2012 |
| *SLC39A14* | Affected children presented with loss of developmental milestones, progressive dystonia and bulbar dysfunction in infancy or early childhood. Towards the end of the first decade, they developed severe generalized pharmacoresistant dystonia, spasticity, limb contractures and scoliosis, and lost independent ambulation. Some showed parkinsonian features of hypomimia, tremor and bradykinesia | Microcephaly, scoliosis, ID, spasticity, hyperreflexia, ankle clonus, | ↑blood manganese | BG T1W hyperintensities, BA, CeA | Chelation with CaNa2EDTA | Tuschl et al., 2016 |
| *SLC39A8* | Dystonia by 3 month of age. | Short stature, SNHL, strabismus, nystagmus, hypotonia, severe ID, S, recurrent infections | Abnormal TIFT type 2 pattern, ↓plasma zinc and manganese, ↑urine zinc and manganese | BA, CeA | Uridine, galactose, manganese | Riley et al., 2017 |
| **Disorders of zinc metabolism** | | | | | | |
| *SLC30A9* | Profound ataxia of limbs and muscle weakness was present at various degrees in all patients. Mild dyskinesia was evident in all patients: most had mild choreoathetosis and dystonic postures of limbs. Older patients had marked axial hypotonia and had difficulties in walking, with camptocormia (‘bent trunk’) postures. | Oculomotor apraxia, ptosis, renal failure, truncal hypotonia, cognitive regression | Hyperkalemia (¿kidney disease?) | Normal | None | Perez et al., 2017 |
| **Disorders of selenium metabolism** | | | | | | |
| *SEPSECS* | Intermittent and mild choreiform movements were seen in 50% of patients | Microcephaly, contractures, ID, S | No | CeA, BA, DM, thin CC, periventricular WM abnormalities | None | Ben-Zeev et al., 2003 |
| DISORDERS OF CARBOHYDRATES | | | | | | |
| **Disorders of carbohydrate transport and absorption** | | | | | | |
| *SLC2A1* | A complex MD is commonly seen and is characterized by ataxia, dystonia, and chorea that may be continuous, paroxysmal, or continual with fluctuations determined by environmental stressors. Often, paroxysmal worsening occurs before meals, during fasting, or with infectious stress.  Clinical findings included the following: Gait disturbance (89%), the most frequent being ataxia and spasticity together or ataxia alone, action limb dystonia (86%), mild chorea (75%),  cerebellar action tremor (70%), non-epileptic paroxysmal events (28%), dyspraxia (21%) and myoclonus (16%)  Paroxysmal movement disorders. Paroxysmal exercise-induced dyskinesia and paroxysmal choreoathetosis are now recognized to be part of the phenotypic spectrum of Glut1 DS.  Other associated findings included progressive spastic paraparesis with onset in early adulthood, mild gait ataxia, mild-to-moderate cognitive impairment, and epileptic seizures.  It is unclear whether these events represent epileptic or non-epileptic phenomena. | Microcephaly, S, hyperreflexia, ID | ↓CSF glucose, ↓CSF lactate | Normal | Ketogenic diet or triheptanoin | Wang et al., 2018 |
| **Disorders of galactose metabolism** | | | | | | |
| *GALT* | MD in 48% of patients. Dystonia is the major feature, with additional tremor in adults, and myoclonus in children. | Failure to thrive, cataracts, hepatomegaly, vomiting, ID, E. coli sepsis | Hemolytic anemia, metabolic acidosis, ↑transaminases, Galactose-1-phosphate uridyltransferase deficiency, galactosuria, aminoaciduria, albuminuria | WM hyperintensities | Low-galactose diet | Kuiper et al., 2019 |
| **Disorders of the pentose phosphate pathway and polyol metabolism** | | | | | | |
| *RPIA* | 75% of reported patients show cerebellar ataxia | Nystagmus, optic atrophy, retinal degeneration, ID, S, spasticity, PN | ↑urine and CSF ribitol and D-arabitol | Leukoencephalopathy | None | Kaur et al, 2019 |
| **Disorders of gluconeogenesis** | | | | | | |
| *PC* | A, D, HRS, T | Hepatomegaly, proximal renal tubular acidosis, ID, hypotonia, S, ankle clonus | ↑blood lactate, pyruvate and alanine, hypoglycemia, ↑serum ammonia, citrulline and lysine, ↑lactate: pyruvate ratio | BA, periventricular cysts and leukomalacia, DM, subcortical leukodystrophy | None | Wang et al., 2018 |
| **Disorders of glycolysis** | | | | | | |
| *TPI1* | Extrapyramidal neurologic including hyperkinetic torsion dystonia limited to the right shoulder girdle and cervical muscles, involuntary choreoathetoid torsion of the neck and intermittent tremor have been described. | Pale optic disc, kyphosis, jaundice, hypotonia, spasticity, PN, frequent infection | Hemolytic anemia, ↑ tissue and red cells DHAP | Normal | None | Sarper et al., 2013 |
| MITOCHONDRIAL DISORDERS OF ENERGY METABOLISM | | | | | | |
| **Disorders of pyruvate metabolism** | | | | | | |
| *PDHA1*  *PDHX* | Isolated paroxysmal exercise induced dystonia and intermittent isolated ataxia have been described. Late onset (mid-thirties) atypical parkinsonism, choreiform movements, stereotypies, ataxia have also been reported. | Low birth weight, microcephaly, episodic ptosis, hypotonia, ID, S, facial dysmorphism (less frequent) | ↑blood and CSF lactate and pyruvate, ↑blood alanine, ammonia | BG T2W hyperintensities, BA, agenesis of corpus callosum, ↑lactate on MRS | Thiamine, ketogenic diet | Castiglioni et al., 2015  Debray et al. 2008  Mellick et al., 2004  Head et al., 2004 |
| **Disorders of the Krebs cycle** | | | | | | |
| *ACO2* | Truncal ataxia and dystonic hand movements were dominant within the first 3 years of life and gradually decreased concomitantly with further motor regression. | Failure to thrive, microcephaly, SNHL, optic atrophy, retinal dystrophy, strabismus, nystagmus, ID, S, hypotonia, hyporeflexia, PN | Metabolic acidosis, hyperglycemia | BA, CeA, thin CC, WM abnormalities | None | Sharkia et al., 2019 |
| *SUCLA2* | Early-onset dystonia/hyperkinesia-deafness syndrome. Dystonia in 85% of patients. | Failure to thrive, SNHL, ophthalmoplegia, ptosis, strabismus, hypotonia, ID, spasticity, hyporeflexia, S, PN | ↑blood and CSF lactate, ↑CK, methylmalonic aciduria, methylglutaconic aciduria, intermittent aminoaciduria | BG T2W hyperintensities, BA | None | Maas et al., 2016 |
| *SUCLG1* | Dystonia (40% of patients) | Failure to thrive, SNHL, hypotonia, ID | ↑blood and CSF lactate, hypoglycemia, methylmalonic aciduria, abnormal mitochondrial RCC activities | BG T2W hyperintensities, BA | None | Carrozzo et al., 2016 |
| *FH* | Dystonia, ataxia and intention tremor have been reported | Failure to thrice, optic atrophy, cholestasis, cutaneous leiomyomata, hypotonia, S, ID, | Metabolic acidosis, ↑lactate and pyruvate, urine fumaric, malic and succinic acids | Polymicrogyria, decreased WM volume, CC agenesis | None | Bourgeron et al., 2014 |
| *MDH2* | Dystonia | Failure to thrive, strabismus, retinitis pigmentosa, hypotonia, encephalopathy, S | ↑serum and CSF lactate, abnormal RCC | CeA, DM, hypoplastic CC | None | Ait-El-Mkadem et al., 2017 |
| *SLC13A5* | Patients show variable combination of ataxia, choreoathetosis and dystonia. | Microcephaly, hypodontia or delayed eruption, hypotonia, encephalopathy, S, axial hypotonia, spasticity | None | WM abnormalities, DM, ↑lactate in MRS | Ketogenic diet (seizure) | Hardies et al., 2015 |
| **Disorders of metabolite repair** | | | | | | |
| *L2HGA* | Cerebellar ataxia can be found in 20% of patients at onset and 82% during disease course. Patients can also show L-dopa responsive dystonic posturing of head, trunk and upper limbs (age onset: 10 years old) and intentional tremor. | SNHL, optic atrophy, strabismus, nystagmus, ID, S | ↑serum lysine, ↑ serum, urinary and CSF L-2-hydroxyglutaric acid | Leukoencephalopathy with cavitation, BA, CeA | None | Steenweg et al., 2010  Balaji et al., 2014 |
| **Disorders of mitochondrial carriers** | | | | | | |
| *SLC25A12* | Dystonia, spasticity | Hypotonia, encephalopathy, S, ID, spasticity, hyperreflexia | ↑serum lactate | Hypomyelination | Ketogenic diet | Falk et al., 2014 |
| **Disorders of mitochondrial complex subunits and assembly** | | | | | | |
| Leigh Syndrome | Dystonia is a common feature in Leigh syndrome. Choreic movements have been found in Leigh syndrome, especially in children with ATPase 6 point mutations. Myoclonus has also been reported in some patients. | Failure to thrive, ophthalmoplegia, optic atrophy, nystagmus, strabismus, ptosis, pigmentary retinopathy hypotonia, hypertrichosis, ID, spasticity, hyperreflexia, S | ↑blood and CSF lactate | BG, cerebellum and brainstem T2W hyperintensities, ↑lactate on MRS | None | Tranchant et al., 2016 |
| MELAS | Ataxia in 25%-49%of patients. | SNHL, cataracts, ophthalmoplegia, cardiac conduction abnormalities, cardiomyopathy, episodic vomiting, myopathy, headache, S, hemiparesis, diabetes mellitus | ↑blood lactate | Stroke like episodes, ↑lactate on MRS | None | El-Hattab et al., 2018 |
| MERFF | Frequently adult-onset ataxia and myoclonus | S, spasticity, SNHL | ↑blood lactate, ↑pyruvate | CeA, atrophy of superior cerebellar peduncles | None | Melone et al., 2004 |
| NARP *(MT-ND6, MT-ATP6 >50% individuals)* | Proximal neurogenic muscle weakness with sensory neuropathy, ataxia, and pigmentary retinopathy. Symptoms usually start in childhood. | Retinitis pigmentosa, nystagmus, ID, S, PN | None | Normal, BA, CeA | None | Thorburn et al., 2017 |
| **Disorders of mitochondrial DNA depletion** | | | | | | |
| *POLG* | Movement disorders, primarily myoclonus and choreoathetosis, are common. Myoclonus can be difficult to distinguish from myoclonic seizures and EPC. Palatal myoclonus resulting from involvement of the inferior olivary nuclei can be seen as well. Some develop parkinsonism, which may temporarily respond to levodopa. Neuropathy and ataxia develop in all persons with Alpers-Huttenlocher Syndrome unless the disease process is so rapid that it results in early death. All neurologic signs and symptoms, including ataxia and nystagmus, may worsen during infections or with other physiologic stressors.  Myoclonic Epilepsy Myopathy Sensory Ataxia (MEMSA): Cerebellar ataxia, generally the first sign, begins in young adulthood as a subclinical sensory polyneuropathy. | SNHL, nystagmus, ptosis, ophthalmoplegia, cataracts, cardiomyopathy, intestinal pseudo-obstruction, muscle weakness, hyporeflexia, migraine, S, ID, PN | ↑blood lactate and CK | Cerebellar WM and thalamic abnormalities | None | Cohen et al., 2018 |
| *MPV17* | Ataxia (3% of patients), dystonia (4% patients ) | Failure to thrive, nystagmus, hepatomegaly, acute hepatic failure, steatosis, cholestasis, vomiting, hypotonia, ID, PN, hyporeflexia, pain insensitivity | Hypoglycemia, ↑blood lactate, transaminases | WM and BG abnormalities, brainstem abnormalities | None | El-Hattab et al., 2018 |
| *TWNK* | Normal development until age one year, followed by onset of ataxia, muscle hypotonia, loss of deep-tendon  reflexes, and athetosis | SNHL, nystagmus, ophthalmoplegia, pes cavus, S (rare), PN, hyporeflexia, amenorrhea | Hypergonadotropic hypogonadism, ↑blood lactate and pyruvate | CeA, BA, cerebellar T2W WM abnormalities | None | Lönnqvist et al., 2013 |
| *FBXL4* | 30% of patients show severe truncal ataxia, dystonia, choreoathetoid movements | Failure to thrive, microcephaly, dysmorphic features, cataracts, nystagmus, hypertrophic cardiomyopathy, hypospadias, renal tubular acidosis (rare), scoliosis, hypotonia, encephalopathy, severe nonverbal ID, S, recurrent infections | ↑blood lactate, ammonia, alanine and transaminases, neutropenia, abnormal RCC | BA, leukodystrophy, CeA, BG and WM abnormalities, DM, thin CC | None | Gai et al., 2013 |
| **Disorders of mitochondrial translation factors** | | | | | | |
| *C12orf65* | Early onset ataxia (18 months of age) | Failure to thrive, optic atrophy, ophthalmoplegia, cognitive regression, nystagmus , PN | ↑blood lactate, abnormal RCC | T2W thalami, brainstem and medulla spinalis abnormalities | None | Antonicka et al., 2010 |
| **Disorders of mitochondrial tRNA incorporation and recycling** | | | | | | |
| *DARS2* | Slowly progressive ataxia, tremor (age onset 2 to 15 years). Exercise induced ataxia has been reported. Tremor, | Nystagmus, muscle weakness, ID, spasticity, hyperreflexia, PN | None | Leukoencephalopathy including brainstem, cerebellum and spinal cord, ↑lactate on MRS | None | Finsterer et al., 2017 |
| *MARS2* | Autosomal Recessive Spastic Ataxia with Leukoencephalopathy (ARSAL). Cerebellar ataxia, dystonia (57%) | Mild SNHL, nystagmus, scoliosis, spasticity, hyperreflexia, mild ID, | None | BA, WM abnormalities, ↑lactate on MRS | None | Webb et al., 2015 |
| *WARS2* | Neurodevelopmental disorder, mitochondrial, with abnormal movements and lactic acidosis, with or without seizures (NEMMLAS). Childhood onset ataxia and HRS. Dystonia and choreoathetosis have also been described. | Intrauterine growth retardation, nystagmus, optic atrophy, strabismus, retinitis pigmentosa, cardiomyopathy, hypotonia, ID, S(rare) | ↑blood lactate, hypoglycemia, abnormal RCC | BA, DM, periventricular WM abnormalities, CeA, ↑lactate on MRS | None | Virdee et al., 2019 |
| **Disorders of mitochondrial fusion** | | | | | | |
| *OPA1* | Adult onset ataxia, cervical dystonia and upper limb intention tremor | Optic atrophy, SNHL, ptosis, ophthalmoplegia, PN | ↑plasma and urine lactate, alanine | Normal to BA | None | Liskova et al., 2013 |
| *OPA3* | Motor symptoms in the 2 patients started at the age of 3 to 4 years and included chorea, cerebellar ataxia, dystonia, and pyramidal tract signs. | Optic atrophy, spasticity, hyperreflexia, ID | ↑urine 3-methylglutaconic acid | Normal | None | Arif et al., 2013 |
| *MSTO1* | Cerebellar ataxia (60%), tremor | Failure to thrive, SNHL, retinitis pigmentosa (rare), scoliosis, pes cavus, hypotonia, muscle weakness, lipomas, psychiatric abnormalities, amenorrhea. ID. EMG: myopathic pattern | Hyperthyroidism, hyperprolactinemia, ↑CK | CeA, WM hyperintensities | None | Donkervoort et al., 2019 |
| **Disorders of mitochondrial phospholipid metabolism** | | | | | | |
| *SERAC1* | Starting at a median age of 6 months, muscular hypotonia (91%) was seen, followed by progressive spasticity (82%, median onset 5 15 months) and dystonia (82%, 18 months). The majority of affected individuals never learned to walk (68%). | Failure to thrive, microcephaly, SNHL, optic atrophy, neonatal hepatic dysfunction, hypotonia, ID, spasticity, S, recurrent infections, neonatal sepsis | ↑blood lactate, ↑transaminases, ↑AFP, hypoglycemia, coagulopathy, 3-methylglutaconic aciduria, ↓cholesterol | BG T2W hyperintensities sparing central putamen, BA, CeA, ↑lactate on MRS | None | Maas et al., 2017 |
| **Disorders of mitochondrial protein import** | | | | | | |
| *DNAJC19* | Non-progressive cerebellar ataxia | Prenatal growth failure, optic atrophy, dilated cardiomyopathy, long QT syndrome, steatosis, hypospadias, cryptorchidism, ID | Microcytic anemia, ↑transaminases,  3-methylglutaconic aciduria, 3-methylglutaric aciduria | BG T2W hyperintensities | None | Ucar et al., 2017 |
| *TIMM8A* | Deafness-dystonia-optic neuronopathy (DDON) syndrome is a progressive disorder. Dystonia and ataxia may appear in adolescent. One case of rapidly progressive dystonia who died at 16 years of age. | SNHL, cortical blindness, fractures, spasticity, hyperreflexia, mental regression, behavioral abnormalities | None | BG atrophy in males older than 40 years of age | None | Tranebjærg et al., 2019 |
| **Disorders of mitochondrial protein quality control** | | | | | | |
| *PMPCA* | All patients shown nonprogressive early onset cerebellar ataxia (< 2years of age), tremor | Short stature, nystagmus, pes cavus, hypotonia, ID, spasticity, hyperreflexia | None | Cerebellar hypoplasia |  | Jobling et al., 2015 |
| *CLPB* | Severe cases with hyperekplexia or absence of voluntary movements in the neonatal period. Moderate cases with progressive movement disorder (ataxia, dystonia, and/or dyskinesia) of varying severity | Failure to thrive, microcephaly, cataracts, neonatal hypotonia, ID, spasticity, S, recurrent infections | Neutropenia, ↑urinary 2-methylglutaconic acid | BG atrophy, BA, CeA | None | Wortmann et al., 2016 |
| *SACS* | Mild to moderate early onset gait ataxia (age at onset 16-18 months of age) (33%), tremor (10%) | Nystagmus, pes cavus, PN, spasticity, hyperreflexia, ID (rare) | None | Vermis atrophy | None | Vill et al., 2018 |
| *AFG3L2* | Early onset spastic ataxia-neuropathy syndrome (range at onset 6 to 60 years), dystonia, HRS | Nystagmus, eye movement abnormalities, ptosis, ophthalmoparesis, spasticity, hyperreflexia | None | CeA and pontine atrophy, thinning of CC | None | Pierson et al., 2011 |
| *PRKN* | Account for 75% of the juvenile Parkinson Disease (onset before 20 years of age). Dystonia may also be present, typically affecting the lower limbs, and is the very first symptom in 40% of case, sometimes as paroxysmal exercise-induced dyskinesia/ dystonia | Hyperreflexia | None | None | L-dopa responsive | Schneider et al., 2010 |
| *HTRA2* |  |  |  |  |  | Kovacs-Nagy et al., 2018 |
| **Primary CoQ10 deficiencies** | | | | | | |
| *COQ2* | Cerebellar ataxia and tremor | SNHL, nystagmus, retinitis pigmentosa, hypertrophic cardiomyopathy, nephrotic syndrome, glomerulosclerosis, encephalopathy, S, ID, Muscle biopsy: RRF, lipid accumulation | ↑plasma lactate and CK, anemia, pancytopenia, ↓coenzyme Q10 in muscle | CeA | Coenzyme Q10 | Scalais et al., 2013 |
| *COQ6* | Ataxia | SNHL, nephrotic syndrome, glomerulosclerosis, S(rare) | Proteinuria | Normal to CeA | Coenzyme Q10 | Heeringa et al. 2011 |
| *COQ8A* | Slowly progressive cerebellar ataxia, intentional tremor, myoclonic jerks. Age at onset: 6 years of age. | Pes cavus, hypotonia, pyramidal signs, variable ID and S. Muscle biopsy: lipid accumulation | ↑plasma and CSF lactate, abnormal RCC, ↓coenzyme Q10 in muscle | CeA | Coenzyme Q10 | Terraciano et al., 2012 |
| DISORDERS OF LIPIDS | | | | | | |
| **Disorders of ketone body metabolism** | | | | | | |
| *ACAT1* | Delayed onset (6 months after decompensation) generalized dystonia and nonprogressive continuous chorea are the main MD. Ataxia and myoclonic jerks have been also described. MD may occur even without a previous decompensation. Decompensation may be triggered by febrile illness. | Vomiting, ID | Episodic ketoacidosis and metabolic acidosis. OA = ↑ 2-methyl-3-hydroxybutyrate, 2-methylacetoacetate and tiglylglycine. ↑ C5:1 acylcarnitine. | BG abnormalities | Protein restriction, carnitine supplementation | Buhas et al., 2013,  Wojcik et al., 2017, Yalçinkaya et al., 2001 |
| **Disorders of fatty acid synthesis and elongation** | | | | | | |
| *MECR* | Childhood-onset progressive dystonia, facial chorea, dyskinesias and myoclonus: ages 1-6.5 years. | Optic atrophy, nystagmus, spasticity, hyperreflexia | ↑CSF lactate, abnormal mitochondrial RCC activities | BG hyperintensities | None | Heimer et al., 2019 |
| *ELOVL4* (SCA34) | AD inheritance: adult onset (30-40 years of age) slowly progressive ataxia, pyramidal tract signs, and cerebellar and pontine atrophy detected on MRI, erythrokeratodermia may be present. AR inheritance: spastic paraplegia, ichthyosis and ID. | Nystagmus, supranuclear gaze palsy, erythrokeratodermia, spasticity, PN | None | CeA, pontine atrophy, pontine midline linear hyperintensity | None | Ozaki et al., 2015 |
| *ELOVL5* | Adult onset cerebellar ataxia (onset 30-50 years of age) | Nystagmus, PN | None | CeA | None | Di Gregorio et al., 2014 |
| **Disorders of the fatty alcohol cycle** | | | | | | |
| *ALDH3A2* | Teenage-onset dystonia and tremor precipitating neurodegeneration without any immediate causal events. Prominent dysarthria due to dystonia. | Short stature, ID, spastic diplegia generalized ichthyosis |  | nonspecific T2W hyperintensities at bilateral parietal deep WM | None | Cho et al., 2018 |
| **Disorders of intracellular triglyceride metabolism** | | | | | | |
| *ABHD5* | Ataxia | SNHL, subcapsular cataracts, nystagmus, strabismus, hepatomegaly, steatosis, ichthyosiform erythroderma, alopecia, ID, myopathy | Lipid droplets in granulocytes | Normal | None | Nakhaei et al., 2018 |
| *BSCL2* | The spectrum includes Silver syndrome and variants of Charcot-Marie-Tooth neuropathy type 2, distal hereditary motor neuropathy (dHMN) type V, spastic paraplegia 17 and Celia´s encephalopathy (progressive Encephalopathy with/without Lipodystrophy, PELD). Myoclonic seizures, stereotypies, including orolingual dystonia with tongue protrusion. Gait ataxia, tremor. | Spasticity, hyperreflexia, amyotrophy, paresthesia, pes cavus, ID, congenital generalized lipodystrophy | None | Normal to BA | Metreleptin, PUFA-rich diet | Ito et al., 2018, Sanchez-Iglesias et al., 2019 |
| **Disorders of non-mitochondrial phospholipid metabolism** | | | | | | |
| *ATP8A2* | All patients show MD, specifically chorea or choreoathetosis (100%), dystonia (27%) and facial dyskinesia (18%). Symptoms onset before 6 months of age. | ID, severe hypotonia, optic atrophy | None | Normal to BA, CeA, CC atrophy, hypoplastic optic nerves | None | McMillan et al., 2018 |
| *PLA2G6* | PLAN encompasses a continuum of three overlapping phenotypes: 1) infantile onset PLAN, corresponding to classic infantile neuroaxonal dystrophy, 2) childhood-onset PLAN corresponding to atypical neuroaxonal dystrophy (ANAD) and 3) juvenile adult-onset PLAN corresponding to PLA2G6-related dystonia-parkinsonism. These patient exhibit predominantly HRS, resting tremor and limbs, oromandibular or generalized dystonia. | Psychomotor regression, hypotonia, pyramidal tract signs, a/hyperreflexia, S, PN | None | CeA, BA, cerebellar cortex T2W hyperintensities, thin optic chiasm, BG iron deposition | None | Darling et al., 2019 |
| *PNPLA6* | The disorder in most patients was characterized by the triad of spinocerebellar ataxia, hypogonadotropic hypogonadism, and visual impairment due to chorioretinal dystrophy. The age at onset was variable, but most patients developed one or more symptoms in the first decade of life. | PN, chorioretinal dystrophy, spasticity, mild ID | Hypogonadotropic hypogonadism | CeA, spinal cord atrophy, small pituitary gland | None | Synofzik et al., 2014 |
| *ABHD12* | Polyneuropathy, hearing loss, ataxia, retinitis pigmentosa, and cataract (PHARC) syndrome. Slowly progressive ataxia, intention tremor. Variable age at onset: 3 to 43 years of age. | SNHL, subcapsular cataracts, retinitis pigmentosa, optic atrophy, nystagmus, pes cavus, spasticity, PN | None | CeA | None | Fiskerstrand et al., 2010 |
| **Disorders of non-lysosomal sphingolipid metabolism** | | | | | | |
| *FA2H* | Variable phenotype NBIA, SPG35 (spastic paraparesis) and leukodystrophy (dystonia). Early childhood onset predominantly lower limb spastic tetraparesis and truncal instability, cerebellar ataxia, and cognitive deficits, often accompanied by movement disorders. The disease is rapidly progressive with loss of ambulation after a median of 7 years after disease onset | Ophthalmoplegia, optic atrophy, strabismus, nystagmus, spasticity, hyperreflexia, cognitive decline, S, | None | Thin CC, CeA, leukodystrophy, periventricular WM and T2W WM abnormalities, iron deposition in globus palidus | None | Rattay et al., 2019 |
| *GBA2* | Variable age at onset (4 to 15 years of age) cerebellar ataxia, head tremor | SNHL, cataracts, nystagmus, scoliosis, pes cavus, spasticity, hyperreflexia, PN | None | BA, CeA, thin CC | None | Hammer et al., 2013 |
| **Disorders of palmitoylation** | | | | | |  |
| *PPT1* | Between 6 and 24 months there is rapid psychomotor regression, ataxia, myoclonus, seizures, and visual failure. Ultimately, patients become vegetative, with prominent spasticity. Late infantile variant begins between 2 and 4 years of age. Patients show ataxia and myoclonus. Juvenile or adult-onset patients may present cognitive decline, ataxia and HRS. | Optic atrophy, retinal degeneration, progressive vision loss, cognitive decline, hypotonia, S, spasticity, | ↓ PPT1 enzyme activity | BA, CeA, T2W thalamus and BG hyperintensities | None | Mink et al., 2013 |
| **Disorders of lipoprotein metabolism** | | | | | | |
| *APOB* | Childhood onset ataxia, resembling ataxia with vitamin E deficiency | Retinitis pigmentosa, retinal degeneration, hyporeflexia | Acanthocytosis, hypobetalipoproteinemia, ↓serum cholesterol | Normal | None | Homer et al., 2005 |
| *MTTP* | Spinocerebellar ataxia. Friedrich's-like ataxia in adulthood | Retinopathy, PN | Acanthocytosis, abetalipoproteinemia, | Normal | None | Burnett et al., 2018 |
| **Disorders of cholesterol biosynthesis** | | | | | | |
| *MVK* | Progressive ataxia | Failure to thrive, microcephaly, cataracts, nystagmus, hepatomegaly, splenomegaly, vomiting, scoliosis, ID, hypotonia, recurrent fever | Anemia, thrombocytopenia, ↑IgD, CK, transaminases, N or ↓serum cholesterol, ↑urine mevalonic acid | CeA, BA, agenesis of cerebellar vermis | None | Kellner et al., 2017 |
| **Disorders of bile acid synthesis** | | | | | | |
| *ACOX2* | Mild infantile onset gait ataxia and dysmetria | Vertical gaze palsy, liver fibrosis, ID | ↑serum and urine C27 bile acid intermediates DHCA and THCA and transaminases, ↓cholesterol. | Normal to WM abnormalities | None | Vilarinho et al., 2016 |
| *AMACR* | Cerebellar ataxia and tremor, onset since second decade | S | ↑serum C26-bile-acid intermediates, pristanic acid and variable phytanic acid. | Nonspecific WM hyperintensities. | None | Dick et al., 2011 |
| *CYP7B1* | Childhood-onset, very slowly progressive sensory ataxia, postural instability and a tendency to fall when closing eyes may be a prominent clinical presentation | ID, optic atrophy, SNHL and cognitive decline. Abnormal SSEP in almost all cases | ↑ plasma and CSF 27-hydroxycholesterol | Normal to T2W WM abnormalities | None | Di Fabio et al., 2014 |
| *CYP27A1* | age at onset of MD: range 13–62 years. MD can be considered a late disease manifestation. Parkinsonism is the most frequently reported MD, followed by dystonia, myoclonus and postural tremor. Mixed MD 23% patients. MD is the presenting symptom 18% patients. | Cognitive impairment, psychiatric symptoms, pyramidal signs, diarrhea, juvenile cataract, tendon xanthomas (Achilles tendon or tibial tuberosity) and xanthelasma | ↑plasma cholestenol and urinary 7 alpha-hydroxylated bile alcohols, and variable plasma cholesterol | BA, CeA, cerebellar WM and dentate nuclei abnormalities, cerebral peduncles and internal capsule abnormalities | Chenodeoxycholic acid | Stelten et al., 2019 |
| **Disorders of tetrapyrroles** | | | | | | |
| **Disorders of heme metabolism** | | | | | | |
| *CPOX* | Adulthood onset acute ataxia, triggered by lead exposure | Tachycardia, hypertension, hepatomegaly, splenomegaly, abdominal pain, vomiting, photosensitivity, neuropathic attacks, PN | ↑urine coproporphyrin, total porphyrins, uroporphyrinporphobilinogen,  ↓5´-aminolevulinic acid | Normal | Hematin | Jimenez-Jimenez et al., 2013 |
| *CYB5R3* | Early onset (6 month of age) dystonia, torticollis and hyperkinetic movements | Failure to thrive, microcephaly, strabismus, cyanosis, headache, ID, opisthotonos, spasticity, truncal hypotonia | Polycythemia, ↑methemoglobin concentration | Decreased WM volume, hypomyelination | Hematin | Mannino et al., 2018 |
| *HMBS* | Childhood-onset slowly progressive spastic paraparesis, cerebellar ataxia, | PN, vertical gaze palsy, nystagmus | ↑urine and plasma porphobilinogen and 5´-aminolevulinic acid | Periventricular and deep cerebral WM, thalami and central part of the pons abnormalities. CeA | None | Kevelam et al., 2016 |
| **Storage disorders** | | | | | | |
| **Disorders of autophagy** | | | | | | |
| *SNX14* | Progressive early onset cerebellar hypotrophy leading to ataxia with severe intellectual disability | Macrocephaly, coarse facies, SNHL (rare), nystagmus, scoliosis, talipes equinovarus, hypotonia, ID, spasticity, S (rare), hyporeflexia, autistic behavior | None | CeA, BA | None | Bryant et al, 2018 |
| *WDR45* | The affected individuals universally showed an early-onset global developmental delay that was static until adolescence/early adulthood when a secondary neurological decline was noted including HRS, dystonia and dementia | Eye movement abnormalities, retinal atrophy, ID, S | None | CeA, BA, thin CC, atrophy of pons, DM, hypomyelination, iron accumulation | None | Hoffjan et al., 2016 |
| **Neuronal ceroid lipofuscinosis** | | | | | | |
| *ATP13A2* | Juvenile onset (10 to 12 years of age) HRS, tremor, facial-faucial-finger mini-myoclonus, ataxia, dystonia | Supranuclear gaze palsy, oculogyric crisis, torticollis, hyperreflexia, S, cognitive decline, PN | None | BA, CeA, caudate atrophy or hypointensity, iron accumulation | None | Behrens et al., 2010 |
| *CLN5* | Ataxia and myoclonic epilepsy (age onset 4 to 17, mean age 5,6 years) | Vision loss, retinal degeneration, nystagmus, cognitive regression, S | None | CeA | None | Mink et al., 2013 |
| *CLN6* | Ataxia. Variable age at onset: 18 months to 8 years. | Vision loss, retinal degeneration, cognitive regression, S | None | CeA | None | Mink et al., 2013 |
| *CLN8* | Ataxia, myoclonus (age onset 2 to 7) | Cognitive decline, vision loss, S | None | CeA, BA | None | Mink et al., 2013 |
| *CTSD* | Childhood juvenile and adulthood-onset ataxia (age at onset 8 to 43 years of age) which progressed to significant motor impairment and cognitive decline. | Microcephaly, retinitis pigmentosa, retinal atrophy, spasticity, S, PN | Decrease or absence of cathepsin D (CTSD) protein immunostaining | CeA, BA | None | Hersheson et al., 2014 |
| *CTSF* | Tremor, ataxia, myoclonus, perioral dyskinesias (adult onset, youngest patient 20 years of age) | Cognitive decline, S | None |  | None | Bras et al., 2016 |
| *DNAJC5* | Myoclonus, cerebellar ataxia, HRS (adult onset 3-4 decades) | S, cognitive decline, | None | CeA, BA | None | Mink et al., 2013 |
| *GRN* | Myoclonic seizures, mild ataxia (age at onset: 22 years of age) | Optic atrophy, retinal dystrophy, S, cognitive decline | None | CeA | None | Smith et al., 2012 |
| *KCTD7* | Early onset gait ataxia and myoclonus | ID, Microcephaly, optic atrophy (rare) | None | CeA, BA, thin CC | None | Ebrahimi‐Fakhari et al., 2017 |
| *MFSD8* | Childhood onset (1,5 to 7 years) progressive ataxia, myoclonic seizures | Optic atrophy, retinopathy, cognitive decline, S | None | CeA, BA | None | Zare-Abdollahi et al., 2019 |
| *TPP1* | Milder MD rest and intention tremor, ataxia, myoclonus, and, ultimately, spastic quadriparesis follow. The myoclonus may be severe and refractory to treatment. | Vision loss, retinal degeneration, cognitive regression, S, | ↓ TPP1 enzyme activity | BA | Intraventricular infusion of cerliponase alfa-recombinant human tripeptidyl peptidase-1 every 2 weeks lifelong | Ebrahimi‐Fakhari et al., 2017 |
| *CLN3* | Cerebellar ataxia, myoclonus, and HRS (onset 4 to 10 years) | Retinitis pigmentosa, macular degeneration, optic atrophy, glaucoma, cataracts, cardiomyopathy, cognitive regression, S | Vacuolated lymphocytes | CeA | None | Chueng et al., 2018 |
| **Sphingolipidosis** | | | | | | |
| *ARSA* | In the typical case the disease starts at the age of about 18 months. Children lose acquired capabilities, develop a spastic tetraparesis, dysarthria, dementia, and finally die in a decerebrate state. Children may exhibit ataxia, dystonia and choreoathetosis | Optic atrophy, cholecystitis, ID, hypotonia, hypo/hyperreflexia, S, PN | ↑CSF protein, ↓ arylsulfatase A activity | WM abnormalities, | Bone marrow transplant | Ferreira et al., 2017 |
| *GBA* | Ataxia and myoclonus | Failure to thrive, supranuclear ophthalmoplegia, nystagmus, strabismus, corneal opacities, oculomotor apraxia, macular atrophy, cardiomegaly, hepatomegaly, splenomegaly, interstitial lung disease, skeletal abnormalities, pes cavus, S, variable ID | Pancytopenia, thrombocytopenia, anemia, ↓beta-glucosidase protein and activity | Hydrocephalus, BA | Enzyme replacement therapy with glucocerebrosidase, substrate reduction | Ferreira et al., 2017 |
| *GLB1* | Type I: Extrapyramidal signs and dystonia are the major neurologic manifestations in adults with GM1 gangliosidosis. Type III: The most common presentation is that of progressive generalized dystonia, and almost half of all patients have associated HRS. Facial dystonia, seen in approximately 90% of patients, is an important diagnostic clue | Failure to thrive, coarse facies, cherry red spot, gingival hyperplasia, cardiomyopathy, inguinal hernia, hepatomegaly, splenomegaly, scoliosis, angiokeratoma, ID | Vacuolated lymphocytes | Periventricular WM abnormalities | None | Ferreira et al., 2017 |
| *HEXA* | Infantile-onset, rapidly progressive neurodegenerative disease culminates in death before 4 years of age (classic Tay-Sachs disease and Sandhoff disease. HRS (age at onset 2,5 years) with bradykinesia, hypomimia, hypophonia, tremor, postural instability, retropulsion. Gait ataxia, stimulus- induced myoclonus | Pale macule, startle response, hypotonia, S | Vacuolated lymphocytes, ↑urine oligosaccharides, ↓ hexosaminidase A activity | WM volume loss, thin CC, periventricular leukomalacia, abnormal cerebellar cortex | Miglustat | Ebrahimi‐Fakhari et al., 2017  Ferreira et al., 2017 |
| *HEXB* | Sandhoff disease, infantile, juvenile, and adult forms. Juvenile forms can exhibit cerebellar ataxia | Muscle weakness, startle reaction, hyperreflexia, macrocephaly, coarse facies, cherry red spot, cardiomegaly, hepatomegaly, splenomegaly | Vacuolated lymphocytes, ↑urine oligosaccharides, ↓ hexosaminidase A activity | CeA | None | Ferreira et al., 2017 |
| *GALC* | Stage II: rapid severe neurologic deterioration with decorticate posturing. Stage IV: absence of voluntary movement. Palatal myoclonus: rhythmical contractions of the soft palate, pharynx, larynx, lips and tongue at 2 years of age. | Failure to thrive, SNHL, optic atrophy, nystagmus, vomiting, ID, cognitive regression, spasticity, S, PN | ↑CSF protein, ↓beta-galactosidase activity | Hydrocephalus, BA, DM leukodystrophy, intracranial calcification, T2W hyperintensities in midbrain, pons and medulla oblongata | None | Yamanouchi et al., 1991 |
| *PSAP* | Frequent and rhythmic movements of the child in late pregnancy, early onset myoclonus (4 weeks of age) | Hypotonia, ID, spasticity, hypo/hyperreflexia, S, cognitive decline, PN, central apneas, optic atrophy, hepatomegaly, splenomegaly | N or ↓ arylsulfatase A activity, ↑CSF protein | Periventricular WM abnormalities, DM, thin CC | None | Kuchař et al., 2009 |
| *SUMF1* | Ataxia is a clinical sign common to all phenotypes | Coarse facies, SNHL, corneal clouding, retinal degeneration, hepatomegaly, splenomegaly, dysostosis multiplex, ichthyosis, ID, hyperreflexia, spasticity | Abnormal urine mucopolysaccharides | BA, CeA, periventricular WM abnormalities | None | Schlotawa et al., 2019 |
| **Oligosaccharidosis** | | | | | | |
| *CTSA* | Juvenile/adulthood phenotype (Japanese patients): myoclonus and ataxia | Coarse facies, SNHL, ID, S, dysostosis multiplex, corneal clouding, red cherry-spot | ↑urine sialyloligosaccharides, normal sialic acid | Enlarged ventricles, hyperintense WM, thalamic vasculopathy, widened peri encephalic spaces | None | Patel et al, 1999 |
| *FUCA1* | Childhood onset progressive, generalized and painful dystonia (age at onset 5 years of age). Oromandibular dystonia. Fixed dystonia associated with multilevel joint contractures | Coarse facies, SNHL, cardiomegaly, hernia, hepatomegaly, splenomegaly,  dysostosis multiplex, angiokeratoma, ID, hypotonia/spasticity, S, PN | Vacuolated lymphocytes, ↑urine oligosaccharides | BA, DM, eye of the tiger sign mimic | None | Gautschi et al., 2014 |
| *MANBA* | Progressive spasticity and cerebellar ataxia suggesting a spinocerebellar ataxia | Mild coarse facies, SNHL, angiokeratoma, ID, hypotonia, S, PN, recurrent infections | ↑urine mannosyl-N-acetylglucosamine | BA | Enzyme replacement therapy, bone marrow transplant | Labauge et al., 2009 |
| *MAN2B1* | Adolescent- young adulthood onset ataxia in moderate form (Type 2) | Coarse facies, macrocephaly, SNHL, retinal degeneration, nystagmus, hepatomegaly, splenomegaly, inguinal hernia, dysostosis multiplex, ID, hypotonia, spasticity, hyperreflexia, recurrent infections | Vacuolated lymphocytes, ↓immunoglobulins, ↑urine mannose-containing oligosaccharides | CeA, WM abnormalities, DM | None | Malm et al., 2008 |
| *NEU1* | Slowly progressive ataxia and myoclonus (age at onset: 9 years of age) | Coarse facies, SNHL, nystagmus, cherry-red spot, lens opacity, cardiomyopathy, neonatal ascites, hepatomegaly, splenomegaly, inguinal hernia, dysostosis multiplex, muscle weakness, S, ID, hypotonia, hyperreflexia, hydrops fetalis | Vacuolated lymphocytes, proteinuria, ↑ urine sialyloligosaccharides and sialylglycopeptides | Normal | None | Gowda et al., 2017 |
| **Disorders of lysosomal cholesterol metabolism** | | | | | | |
| *NPC1*  *NPC2* | Prominent MD: typically beginning as action dystonia in one limb and gradually spreading to involve all of the limbs and axial muscles. Speech gradually deteriorates, with a mixed dysarthria and dysphonia. Facial, orolingual and limbs severe dystonia, cerebellar truncal and limbs ataxia, gelastic cataplexy. Slowly progressive course. Age at onset: 10 to 14 years of age. | Supranuclear gaze palsy, hepatomegaly, splenomegaly, hypotonia, cognitive regression, spasticity, S, cataplexy, fetal ascites, | Enzyme analysis, Filipin test | MRI of the brain is usually normal until the late stages of the illness. At that time, marked CeA, thinning CC, and mild BA may be seen. | Miglustat | Ebrahimi‐Fakhari et al., 2017  Patterson et al., 2019 |
| **Disorders of lysosomal transport or sorting** | | | | | | |
| *SCARB2* | Adolescent–young adulthood onset, progressive action myoclonus, ataxia and tremor, absence of mental deterioration | Nephrotic syndrome, renal failure, S | Thrombocytopenia, proteinuria | CeA | None | Rubboli et al., 2011 |
| *SLC17A5* | Mild ataxia and intentional tremor, athetoid movements. Typically, Salla disease patients are normal at birth but develop muscular hypotonia and ataxia between 6 and 12 months of age. | Failure to thrive, nystagmus, ID, hypotonia, spasticity, S | ↑urine free sialic acid | Hypomyelination, thin CC | None | Ebrahimi‐Fakhari et al., 2017  Ferreira et al., 2017 |
| **Disorders of peroxisomes** | | | | | | |
| **Disorders of peroxisomal fatty acid oxidation** | | | | | | |
| *ABCD1* | X-linked adrenoleukodystrophy phenotype: ataxia, should be suspected in patients with WM or cerebellar hyperintensities  Adrenomyeloneuropathy phenotype: spastic paraparesis and mild cerebellar ataxia, onset at 19 years of age | SNHL, S, spasticity, cognitive regression | ↑VLCFA, | DM, WM abnormalities | Bone marrow transplant | Chen et al., 2018  Chen et al., 2017 |
| *ACOX1* | Early onset (2 month of age) dystonic episodes and enhanced pyramidal signs | SNHL, dysmorphic features, nystagmus, strabismus, optic atrophy, retinitis pigmentosa, inverted nipples (rare), hepatomegaly, steatosis, hypotonia, S, ID, spasticity | ↑VLCFA, normal serum plasmalogen | Leukodystrophy, DM | None | Masson et al., 2016 |
| *HSD17B4* | Perrault Syndrome: ovarian dysgenesis, SNHL and ataxia. Cervical dystonia, tremor and middle-age onset cerebellar ataxia. Almost all patient surviving to adulthood had ataxia. | Failure to thrive, macrocephaly, dysmorphic features, nystagmus, strabismus, hepatomegaly, cholestasis, steatosis, renal cysts, osteopenia, talipes equinovarus, hypotonia, S, ID, PN | ↑transaminases, VLCFA, normal plasmalogen | Polymicrogyria, DM, abnormal CC, periventricular heterotopia, BA, CeA | None | Matsukawa et al., 2017 |
| *SCP2* | Spasmodic torticollis and dystonic head tremor, at 7 years of age as well as slight cerebellar signs with intention tremor. Dystonic head tremor triggered by stressful situations | Abnormal saccadic eye movement | Hypergonadotropic hypogonadism | Butterfly lesions of the pons | None | Ferdinandusse et al., 2006 |
| *PHYH* | Clinical tetrad of peripheral neuropathy, retinitis pigmentosa, cerebellar ataxia, and ↑ CSF protein | SNHL, cardiomyopathy, skeletal abnormalities, pes cavus, ichthyosis, PN | ↑CSF protein, phytanic acid | Signal intensity changes in the posterior limbs of internal capsules, corticospinal tracts and splenium of the CC | Phytanic acid restriction, lipapheresis | Cakirer et al., 2004 |
| **Disorders of peroxisomal biogenesis** | | | | | | |
| *PEX1, PEX2, PEX3, PEX5, PEX6, PEX7, PEX10, PEX11B, PEX12, PEX13, PEX14, PEX16, PEX19, PEX26* | *PEX2:* slowly progressive cerebellar truncal ataxia at 3,5 and 18 years of age and tremor  *PEX6*: ataxic gait  *PEX7:* cerebellar ataxia  *PEX10:* early onset slowly progressive severe ataxia with intentional tremor  *PEX16*: Mild cervical dystonia, ‘no-no’ head tremor, cerebellar ataxia in preschool years | Dysmorphic facies, failure to thrive, cataract, corneal clouding, nystagmus, retinopathy, retinitis pigmentosa, hepatomegaly, splenomegaly, jaundice, hypotonia, areflexia, S, ID, hypospadias, cryptorchidism, skeletal abnormalities, congenital heart defects, SNHL, variable microcephaly  *PEX7*: Rhizomelic chondrodysplasia punctate | ↑transaminases, CSF protein, VLCFA, phytanic acid, pipecolic acidemia and aciduria, aminoaciduria, ↓plasmalogen, prystanic acid | Polymicrogyria, pachygyria, colpocephaly, heterotopias, subependymal cysts, agenesis/hypoplasia CC, hypoplastic olfactory lobes, DM, BA | Cholic acid | Kumar et al., 2018  Zhang et al., 2019  Sevin et al., 2011  Tran et al., 2014 |
| **Congenital disorders of glycosylation (CDG)** | | | | | | |
| **Disorders of N-linked glycosylation** | | | | | | |
| *PMM2* | Prominent cerebellar ataxia with generalized muscular hypotonia, generalized or segmental/multifocal dystonia, choreoathetosis. Stereotypies and tremor have also been described | Nonimmune hydrops fetalis, ID, hypotonia, S, hyporeflexia, stroke-like episodes, PN, abnormal subcutaneous fat tissue distribution, inverted nipples, joint contractures, hepatomegaly, nephrotic syndrome, cardiomyopathy, retinitis pigmentosa, nystagmus, microcephaly, failure to thrive | ↑transaminases, thrombocytosis, ↓IgA, ↓IgG, ↓Cu, ↓Fe, ↓Zn, ↓cholesterol, ↓albumin, ↓Factor XI, ↓antithrombin III, abnormal TIFT (type 1 pattern), proteinuria, prolonged prothrombin time, hypergonadotropic hypogonadism, hypothyroidism | Olivopontocerebellar hypoplasia, CeA | None | Mostile et al., 2019 |
| *DPAGT1* | Tremor, muscular fasciculations, pathologic moving patterns and hyperexcitability in the neonatal period. After that, patients showed reduced spontaneous movement | Microcephaly, dysmorphic features, cataracts, nystagmus, strabismus, inverted nipples, contractures, hypotonia, ID, S, hyperreflexia | Anemia, hypoproteinemia, ↓antithrombin III, abnormal TIFT type 1 pattern | Normal to BA | None | Würde et al., 2012 |
| *ALG1* | Ataxia (75% of reported patients) and tremor (71% of reported patients) | IUGR, microcephaly, cardiomyopathy (rare), hepatomegaly, splenomegaly, nephrotic syndrome, contracture, ID, areflexia, hypotonia, S, hydrops fetalis | Hypogonadism, abnormal TIFT type 1 pattern | BA | None | Morava et al., 2012 |
| *RFT1* | Ataxia, myoclonus | Failure to thrive, microcephaly, SNHL, inverted nipples, hepatomegaly, hypotonia, ID, S, spasticity, hyperreflexia | Abnormal TIFT type 1 pattern and coagulation factors | Normal | None | Quelhas et al., 2019 |
| *ALG6* | Ataxia (50% of reported patients) | Strabismus, hypotonia, areflexia, S, proximal muscle weakness, limb abnormalities | ↑transaminases, ↓Factor XI, XI, antithrombin III, protein C and cholesterol, abnormal TIFT type 1 pattern | Vermis hypoplasia or CeA, BA, CC hypoplasia | None | Morava et al., 2016 |
| *ALG13* | Early onset chorea and dyskinesia | Microcephaly, dysmorphic features, optic atrophy, nystagmus, hepatomegaly, contractures, S, ID, hypotonia, recurrent infections (rare) | Abnormal coagulation factors, abnormal TIFT type 1 pattern | Hydrocephalus, BA, DM | None | Kobayashi et al., 2016 |
| **Disorders of glycosylphosphatidylinositol biosynthesis** | | | | | | |
| *PIGG* | Nonprogressive severe generalized ataxia | IUGR, hypotonia, ID, S, hyporeflexia | Normal alkaline phosphatase | Thin CC, CeA, BA | None | Zhao et al., 2016 |
| *PIGN* | Early onset tremor (71% of reported patients) and choreoathetosis (28% of reported patients) | Macrocephaly, dysmorphic features, nystagmus, atrial septal defect, diaphragmatic hernia (rare), anal stenosis, hydronephrosis, hypotonia, skeletal abnormalities, ID, spasticity, S | None | BA, CeA | None | Maydan et al., 2011 |
| *PGAP1* | Tremor (30% of reported patients), dyskinetic MD (60% of reported patients), stereotypies | Short stature, retinal dystrophy, hypotonia, ID, S, spasticity | Normal alkaline phosphatase | BA, thin CC, CeA, DM | None | Kettwig et al., 2016 |
| *PGAP3* | Hand stereotypies (midline hand stereotypies) and bruxism in 3/5 patients | Microcephaly, dysmorphic features, hypotonia, ID, S | ↑alkaline phosphatase | Hypoplastic CC, vermis and cerebellum | None | Howard et al., 2014 |
| **Disorders of glycolipid glycosylation** | | | | | | |
| *ST3GAL5* | Choreoathetoid and dystonic cerebral palsy, startle myoclonus onset between 2 weeks and 3 months before GTCS seizures | Failure to thrive, microcephaly, SNHL, optic atrophy, vomiting, skin dyspigmentation, profound ID, S, hypotonia | None | Normal to BA | None | Gordon-Lipkin et al., 2018  Simpson et al., 2004 |
| *B4GALNT1* | Early-onset spastic paraplegia, slowly progressive ataxia (55% of patients), facial dyskinesia and dystonia (44% of patients) | Cataracts, nystagmus, scoliosis, pes cavus, spasticity, hyperreflexia, variable ID, PN, hyporeflexia | ↓testosterone | T2W WM hyperintensities | None | Boukhris et al., 2013 |
| **Disorders of dolichol metabolism** | | | | | | |
| *SRD5A3* | Early onset cerebellar ataxia (onset before 3,5 years of age) | Dysmorphic features, coloboma, nystagmus, hypoplasia of the optic disc, ichthyosiform dermatitis, hypertrichosis, ID, hypotonia | Microcytic anemia, abnormal coagulation factors, ↑transaminases, ↓IGF1, IGFBP3 and antithrombin III, abnormal TIFT type 1 pattern | Polymicrogyria, vermis and pituitary gland hypoplasia | None | Kasapkara et al., 2012 |
| *DPM1* | Early onset cerebellar ataxia (onset before 2 years of age), observed in 42% of reported patients, intention tremor | Failure to thrive, microcephaly, nystagmus, strabismus, optic atrophy, retinopathy, hepatomegaly, splenomegaly, contractures, hypotonia, ID, S, | Abnormal TIFT type 1 pattern, ↑transaminases, CK, ↓ antithrombin III, protein S, protein C | Pontocerebellar atrophy, hypomyelination, T2W WM hyperintensities | None | Dancourt et al. 2006 |
| *MPDU1* | Ataxia before 5 years of age | Failure to thrive, microcephaly, amaurosis, nystagmus, strabismus, optic atrophy, apnea, contracture, hyperkeratosis, hypotonia, profound ID, S | Abnormal TIFT type 1 pattern and coagulation factors | BA | None | Kranz et al., 2001 |
| **Glycosylation disorders of vesicular trafficking** | | | | | | |
| *TRAPPC11* | Oromotor and limb choreiform movements, dystonia, ataxia, tremor | IGR, microcephaly, cataracts, strabismus, myopia, alacrimia, hepatomegaly, liver fibrosis, achalasia, scoliosis, hypotonia, Gowers sign, ID, S | ↑CK, transaminases | CeA, BA, reduced WM volume, hypomyelination | None | Larson et al., 2018 |
| *COG4* | Mild ataxia and uncoordinated movements at 3 years of age | Failure to thrive, microcephaly, dysmorphic features, nystagmus, hepatomegaly, splenomegaly, cirrhosis, recurrent diarrhea, hypotonia, ID, S | Abnormal TIFT type 2 pattern and coagulation factors, ↑ transaminases, alkaline phosphatase, | Ba, thin CC | None | Reynders et al., 2009 |
| *COG5* | Generalized chorea and stereotypies. Friedreich’s-ataxia-like phenotypes, including progressive cerebellar ataxia (before 2 years of age) and scoliosis (before 4 years of age). | Hypotonia, ID, scoliosis | Abnormal TIFT | CeA, BA | None | Kim et al., 2017 Mostile et al., 2019 |
| *COG8* | Early onset prominent ataxia (before 2 years of age) and action myoclonus that worsened with age | Acute encephalopathy, hypotonia, ID, S, dysmorphic features, oculomotor apraxia, PN, hyporeflexia | ↑CK, transaminases, transient abnormal coagulation factors, ↓ antithrombin III, protein S, protein C | CeA and brainstem atrophy | None | Foulquier et al., 2007 |
| *GOSR2* | Early onset ataxia (on average at 2 years of age), action myoclonus and myoclonic seizures (onset average at 6,5 years), rest tremor. Transient episodes of motor deterioration triggered by infection and fever. | Scoliosis, S (progressive myoclonic epilepsy), mild ID | ↑CK | Normal to mild BA | None | Dibbens et al., 2017  Ebrahimi‐Fakhari et al., 2017 |
| **Disorder of deglycosylation** | | | | | | |
| *NGLY1* | All affected individuals exhibited choreoathetosis, dystonia, myoclonus and action tremor. These movements were more severe in younger individuals. | Microcephaly, alacrimia, corneal ulcerations, strabismus, ocular apraxia, ID, hypotonia, S, PN, hyporeflexia | ↑lactate, AFP, transaminases, abnormal urine oligosaccharides, normal TIFT | DM, prominent Virchow Robin spaces | None | Lam et al., 2017 Mostile et al., 2019 |

**Table S1. IEMs presenting with MD by groups. Abbreviations.** A= ataxia, AAV= adeno-associated virus, AFP = alpha-fetoprotein, BA = brain atrophy, BG = basal ganglia, CA= choreoathetosis, CeA= Cerebellar atrophy CK = creatine kinase, CP = cerebral palsy, D= dystonia, DHAP = dihydroxyacetone phosphate, DM = delayed myelination, GHB = 4-hydroxybutyric acid, GTCS = Generalized tonic–clonic seizures, 5-HIAA = 5-hydroxyindoleacetic acid, HRS= hypokinetic rigid syndrome, HSCT= hematopoietic stem cell transplantation, 5HTP = 5-hydroxytryptophan, HVA= homovanillic acid, ID = intellectual disability, IGR= Intrauterine growth retardation, M = myoclonus, MHPG = 3-methoxy-4-hydroxyphenylethyleneglycol, 3-OMD = 3-ortho-methyldopa, P5C = Delta-1-pyrroline-5-carboxylate, PLP = pyridoxal 5-prime-phosphate, PN = peripheral neuropathy, RCC= respiratory chain complex S= seizures, SAM = S-adenosylmethionine, S-Ado= succinyladenosine, SAICAr= succinylaminoimidazole carboxamide ribotide, SIADH = Syndrome of inappropriate antidiuretic hormone, SNHL = sensorineural hearing loss, T= tremor, TCC= thin corpus callosum, TIBC = total iron binding capacity, TIFT = transferrin isoelectric focusing test, VLCFA= very long-chain fatty acids, VMA = vanillyl mandelic acid, WM = white matter

**References**

1. Koch J et al. (2017). CAD Mutations and Uridine-Responsive Epileptic Encephalopathy. Brain 140(2):279-286.
2. de Brouwer A et al. (2015). Phosphoribosylpyrophosphate Synthetase Superactivity. 2008 Sep 23 [Updated 2015 Dec 17]. In: Adam MP, Ardinger HH, Pagon RA, et al., editors. GeneReviews® [Internet]. Seattle (WA): University of Washington, Seattle; 1993-2020.
3. de Brouwer A et al., (2018). Arts Syndrome. 2008 Oct 21 [Updated 2018 Mar 22]. In: Adam MP, Ardinger HH, Pagon RA, et al., editors. GeneReviews® [Internet]. Seattle (WA): University of Washington, Seattle; 1993-2020.
4. [Fekrvand](https://pubmed.ncbi.nlm.nih.gov/?term=Fekrvand+S&cauthor_id=30885031) S et al. (2019). The First Purine Nucleoside Phosphorylase Deficiency Patient Resembling IgA Deficiency and a Review of the Literature. Immunol Invest 48(4):410-430.
5. Jurecka A et al. (2015). Adenylosuccinate Lyase Deficiency. J Inherit Metab Dis 38(2):231-42.
6. Nyhan W et al. (2014). Lesch-Nyhan Syndrome. 2000 Sep 25 [Updated 2014 May 15]. In: Adam MP, Ardinger HH, Pagon RA, et al., editors. GeneReviews® [Internet]. Seattle (WA): University of Washington, Seattle; 1993-2020.
7. Crow Y. (2016). Aicardi-Goutières Syndrome. 2005 Jun 29 [Updated 2016 Nov 22]. In: Adam MP, Ardinger HH, Pagon RA, et al., editors. GeneReviews® [Internet]. Seattle (WA): University of Washington, Seattle; 1993-2020.
8. Dhar S et al. (2009). Expanded Clinical and Molecular Spectrum of Guanidinoacetate Methyltransferase (GAMT) Deficiency. Mol Genet Metab 96(1):38-43.
9. Anselm I et al. (2006). X-linked Creatine Transporter Defect: A Report on Two Unrelated Boys With a Severe Clinical Phenotype. J Inherit Metab Dis 29(1):214-9.
10. Almusafri F et al. (2017). Clinical and Molecular Characterization of 6 Children With Glutamate-Cysteine Ligase Deficiency Causing Hemolytic Anemia. Blood Cells Mol Dis 65:73-77.
11. [Yapicioğlu](https://pubmed.ncbi.nlm.nih.gov/?term=Yapicio%C4%9Flu+H&cauthor_id=15074378) H et al. (2004). A Newborn Infant With Generalized Glutathione Synthetase Deficiency. Turk J Pediatr 46(1):72-5.
12. Myers J and Shook J. (1996). Vomiting, Ataxia, and Altered Mental Status in an Adolescent: Late-Onset Ornithine Transcarbamylase Deficiency. Am J Emerg Med 14(6):553-7.
13. Keegan C et al. (2003). Acute Extrapyramidal Syndrome in Mild Ornithine Transcarbamylase Deficiency: Metabolic Stroke Involving the Caudate and Putamen Without Metabolic Decompensation. Eur J Pediatr 162(4):259-63.
14. Saini A et al. (2018). Hypomorphic Citrullinaemia Due to Mutated ASS1 With Episodic Ataxia. BMJ Case Rep 2018:bcr2017220193.
15. [Baruteau](https://pubmed.ncbi.nlm.nih.gov/?term=Baruteau+J&cauthor_id=28251416) J et al. (2017). Expanding the Phenotype in Argininosuccinic Aciduria: Need for New Therapies. J Inherit Metab Dis 40(3):357-368.
16. Rocha D et al. (2012). Analysis of Novel ARG1 Mutations Causing Hyperargininemia and Correlation With Arginase I Activity in Erythrocytes. Gene 509(1):124-30.
17. Tunali N et al. (2014). A Novel Mutation in the SLC25A15 Gene in a Turkish Patient With HHH Syndrome: Functional Analysis of the Mutant Protein. Mol Genet Metab 112(1):25-9.
18. Cheon C et al. (2010). Novel Mutation in SLC6A19 Causing Late-Onset Seizures in Hartnup Disorder. Pediatr Neurol 42(5):369-71.
19. Furukawa Y and Kish S. (2014). Tyrosine Hydroxylase Deficiency. 2008 Feb 8 [Updated 2017 May 11]. In: Adam MP, Ardinger HH, Pagon RA, et al., editors. GeneReviews ® [Internet]. Seattle (WA): University of Washington, Seattle; 1993-2020.
20. Wassenberg T et al. (2017). Consensus guideline for the diagnosis and treatment of aromatic l-amino acid decarboxylase (AADC) deficiency. Orphanet J Rare Dis. 12(1):12.
21. Kurian M. (2017). SLC6A3-Related Dopamine Transporter Deficiency Syndrome. 2017 Jul 27. In: Adam MP, Ardinger HH, Pagon RA, et al., editors. GeneReviews ® [Internet]. Seattle (WA): University of Washington, Seattle; 1993-2020.
22. Rilstone J et al. (2013). Brain Dopamine-Serotonin Vesicular Transport Disease and Its Treatment. N Engl J Med 368(6):543-50.
23. Yalaz K et al. (2006). Phenylketonuria in Pediatric Neurology Practice: A Series of 146 Cases. Child Neurol 21(11):987-90.
24. Furukawa Y (2019). GTP Cyclohydrolase 1-Deficient DopaResponsive Dystonia. 2002 Feb 21 [Updated 2019 Jan 24]. In: Adam MP, Ardinger HH, Pagon RA, et al., editors. GeneReviews ® [Internet]. Seattle (WA): University of Washington, Seattle; 1993-2020.
25. Dudesek A et al. (2001). Molecular Analysis and Long-Term Follow-Up of Patients With Different Forms of 6-pyruvoyl-tetrahydropterin Synthase Deficiency. Eur J Pediatr 160(5):267-76.
26. Friedman J. (2015). Sepiapterin Reductase Deficiency. 2015 Jul 1. In: Adam MP, Ardinger HH, Pagon RA, et al., editors. GeneReviews ® [Internet]. Seattle (WA): University of Washington, Seattle; 1993-2020.
27. Opladen T et al. (2020). Consensus guideline for the diagnosis and treatment of tetrahydrobiopterin (BH4) deficiencies. Orphanet J Rare Dis. 15(1):126.
28. Anikster Y et al. (2017). Biallelic Mutations in DNAJC12 Cause Hyperphenylalaninemia, Dystonia, and Intellectual Disability. Am J Hum Genet. 100(2):257-266.
29. Chamberlin M et al. (1996). Demyelination of the brain is associated with methionine adenosyltransferase I/III deficiency. J Clin Invest. 98(4):1021-7.
30. Sacharow S et al. (2017). Homocystinuria Caused by Cystathionine Beta-Synthase Deficiency. 2004 Jan 15 [Updated 2017 May 18]. In: Adam MP, Ardinger HH, Pagon RA, et al., editors. GeneReviews® [Internet]. Seattle (WA): University of Washington, Seattle; 1993-2020.
31. Sloan J et al. (2018). Disorders of Intracellular Cobalamin Metabolism. 2008 Feb 25 [Updated 2018 Sep 6]. In: Adam MP, Ardinger HH, Pagon RA, et al., editors. GeneReviews® [Internet]. Seattle (WA): University of Washington, Seattle; 1993-2020.
32. Bindu P et al. (2017). Isolated Sulfite Oxidase Deficiency. 2017 Sep 21. In: Adam MP, Ardinger HH, Pagon RA, et al., editors. GeneReviews ® [Internet]. Seattle (WA): University of Washington, Seattle; 1993-2020.
33. Grosso S et al. (2002). Ethylmalonic encephalopathy: further clinical and neuroradiological characterization. J Neurol. 249(10):1446-50.
34. Pode-Shakked N et al. (2020). Clues and Challenges in the Diagnosis of Intermittent Maple Syrup Urine Disease. Eur J Med Genet 63(6):103901.
35. Hong Y et al. (2003). Identification of a Common Mutation (Gly194Cys) in Both Arab Moslem and Ashkenazi Jewish Patients With Dihydrolipoamide Dehydrogenase (E3) Deficiency: Possible Beneficial Effect of Vitamin Therapy. J Inherit Metab Dis 26(8):816-8.
36. Sogut A et al. (2004). Isovaleric Acidaemia: Cranial CT and MRI Findings. Pediatr Radiol 34(2):160-2.
37. Wortmann S et al. (2010). 3-Methylglutaconic Aciduria Type I Redefined: A Syndrome With Late-Onset Leukoencephalopathy. Neurology 75(12):1079-83.
38. Ganetzky R and Stojinski C. (2019). Mitochondrial Short-Chain Enoyl-CoA Hydratase 1 Deficiency. 2019 Jun 20. In: Adam MP, Ardinger HH, Pagon RA, et al., editors. GeneReviews ® [Internet]. Seattle (WA): University of Washington, Seattle; 1993-2020.
39. Schottmann G et al. (2013). A Movement Disorder With Dystonia and Ataxia Caused by a Mutation in the HIBCH Gene. Mov Disord 31(11):1733-1739.
40. Olpin S et al. (2002). 2-methyl-3-hydroxybutyryl-CoA Dehydrogenase Deficiency in a 23-year-old Man. J Inherit Metab Dis 25(6):477-82.
41. Jurecki E et al. (2019). Nutrition Management Guideline for Propionic Acidemia: An Evidence- And Consensus-Based Approach. Mol Genet Metab 126(4):341-354.
42. Bikker H et al. (2006). A Homozygous Nonsense Mutation in the Methylmalonyl-CoA Epimerase Gene (MCEE) Results in Mild Methylmalonic Aciduria. Hum Mutat.27(7):640-3.
43. Shevell M et al. (1993). Varying Neurological Phenotypes Among Muto and Mut- Patients With methylmalonylCoA Mutase Deficiency. Am J Med Genet 45(5):619-24.
44. Sloan J et al. (2011). Exome sequencing identifies ACSF3 as the cause of Combined Malonic and Methylmalonic Aciduria. Nat Genet. 43(9):883-6.
45. Fang Y et al. (2019). Clinical and Genetic Analysis of 7 Chinese Patients With β-ureidopropionase Deficiency. Medicine (Baltimore) 98(1):e14021.
46. Chapel-Crespo C et al. (2019). Clinical, Biochemical and Molecular Characteristics of malonyl-CoA Decarboxylase Deficiency and Long-Term Follow-Up of Nine Patients. Mol Genet Metab 128(1-2):113-121.
47. Larson A and Goodman S.(2019). Glutaric Acidemia Type 1. 2019 Sep 19. In: Adam MP, Ardinger HH, Pagon RA, et al., editors. GeneReviews® [Internet]. Seattle (WA): University of Washington, Seattle; 1993-2020
48. Mastrangelo M et al. (2012). A Diagnostic Algorithm for the Evaluation of Early Onset Genetic-Metabolic Epileptic Encephalopathies. Eur J Paediatr Neurol 16(2):179-91.
49. Morales-Briceño H et al. (2019). Paroxysmal Dyskinesias With Drowsiness and Thalamic Lesions in GABA Transaminase Deficiency. Neurology 92(2):94-97.
50. Pearl P et al. (2003). Clinical Spectrum of Succinic Semialdehyde Dehydrogenase Deficiency. Neurology 60(9):1413-7.
51. Lyn C et al., (2015). A Novel Mutation of ALDH5A1 Gene Associated With Succinic Semialdehyde Dehydrogenase Deficiency. J Child Neurol 30(4):486-9.
52. Iwama K et al. (2018). A Novel Mutation in SLC1A3 Causes Episodic Ataxia. J Hum Genet 63(2):207-211.
53. Mérenet A et al. (2012). A Serine Synthesis Defect Presenting With a Charcot-Marie-Tooth-like Polyneuropathy. Arch Neurol 69(7):908-11.
54. Coughlin 2nd C et al (2017). The genetic basis of classic nonketotic hyperglycinemia due to mutations in GLDC and AMT. Genet Med 19(1):104-111.
55. Kanekar S and Byler D. (2013). Characteristic MRI findings in neonatal nonketotic hyperglycinemia due to sequence changes in GLDC gene encoding the enzyme glycine decarboxylase. Metab Brain Dis 28(4):717-20.
56. Bakker et al. (2009). Clonazepam Is an Effective Treatment for Hyperekplexia Due to a SLC6A5 (GlyT2) Mutation. Mov Disord 24(12):1852-4.
57. Ruzzo E et al. (2013). Deficiency of asparagine synthetase causes congenital microcephaly and a progressive form of encephalopathy. Neuron 80(2):429-41.
58. D´Eufemia P et al. (2009). Increased Nitric Oxide Release by Neutrophils of a Patient With Tyrosinemia Type III. Biomed Pharmacother 63(5):359-61.
59. Huemer M et al. (2017). Guidelines for diagnosis and management of the cobalamin-related remethylation disorders cblC, cblD, cblE, cblF, cblG, cblJ and MTHFR deficiency. J Inherit Metab Dis 40:21–48
60. Yu H et al. (2013). An X-Linked Cobalamin Disorder Caused by Mutations in Transcriptional Coregulator HCFC1. Am J Hum Genet 93(3):506-14.
61. Pope S et al. (2019). Cerebral folate deficiency: Analytical tests and differential diagnosis. J Inherit Metab Dis 42(4):655-672.
62. Wolf B. (2019). Biotinidase Deficiency. 2000 Mar 24 [Updated 2016 Jun 9]. In: Adam MP, Ardinger HH, Pagon RA, et al., editors. GeneReviews® [Internet]. Seattle (WA): University of Washington, Seattle; 1993-2020.
63. Aoki Y et al. (1999). Identification and Characterization of Mutations in Patients With Holocarboxylase Synthetase Deficiency. Hum Genet 104(2):143-8.
64. Ortigoza-Escobar J et al. (2017). Thiamine deficiency in childhood with attention to genetic causes: Survival and outcome predictors. Ann Neurol. 82(3):317-330.
65. Houten S et al. (2014). Mitochondrial NADP(H) Deficiency Due to a Mutation in NADK2 Causes dienoyl-CoA Reductase Deficiency With Hyperlysinemia. Hum Mol Genet 23(18):5009-16.
66. Trinh J et al. (2020). Novel NAXE Variants as a Cause for Neurometabolic Disorder: Implications for Treatment. J Neurol 267(3):770-782.
67. Gregory A and Hayflick S. (2017). Pantothenate Kinase-Associated Neurodegeneration. 2002 Aug 13 [Updated 2017 Aug 3]. In: Adam MP, Ardinger HH, Pagon RA, et al., editors. GeneReviews ® [Internet]. Seattle (WA): University of Washington, Seattle; 1993-2020.
68. Evers C et al. (2017). Diagnosis of CoPAN by Whole Exome Sequencing: Waking Up a Sleeping Tiger's Eye. Am J Med Genet A 173(7):1878-1886.
69. Mills P et al. (2006). Mutations in antiquitin in individuals with pyridoxine-dependent seizures. Nat Med. 12(3):307-9.
70. Plecko B et al. (2014). Pyridoxine responsiveness in novel mutations of the PNPO gene. Neurology. 82(16):1425-33.
71. Schuelke M. (2016). Ataxia with Vitamin E Deficiency. 2005 May 20 [Updated 2016 Oct 13]. In: Adam MP, Ardinger HH, Pagon RA, et al., editors. GeneReviews ® [Internet]. Seattle (WA): University of Washington, Seattle; 1993-2020.
72. Atwal P and Scaglia F. (2016). Molybdenum Cofactor Deficiency. Mol Genet Metab 117(1):1-4.
73. Weiss K. (2016). Wilson Disease. 1999 Oct 22 [Updated 2016 Jul 29]. In: Adam MP, Ardinger HH, Pagon RA, et al., editors. GeneReviews ® [Internet]. Seattle (WA): University of Washington, Seattle; 1993-2020.
74. Kaler S. (2016). ATP7A-Related Copper Transport Disorders. 2003 May 9 [Updated 2016 Aug 18]. In: Adam MP, Ardinger HH, Pagon RA, et al., editors. GeneReviews ® [Internet]. Seattle (WA): University of Washington, Seattle; 1993-2020.
75. Chinnery P et al. (2007). Clinical Features and Natural History of Neuroferritinopathy Caused by the FTL1 460InsA Mutation. Brain 130(Pt 1):110-9.
76. Okamoto N et al. (1996). Hereditary Ceruloplasmin Deficiency With Hemosiderosis. Hum Genet 97(6):755-8.
77. Tuschl K et al. (2012). Syndrome of Hepatic Cirrhosis, Dystonia, Polycythemia, and Hypermanganesemia Caused by Mutations in SLC30A10, a Manganese Transporter in Man. Am J Hum Genet 90(3):457-66.
78. Tuschl K et al. (2016). Mutations in SLC39A14 Disrupt Manganese Homeostasis and Cause Childhood-Onset Parkinsonism-Dystonia. Nat Commun 7:11601.
79. Riley L et al. (2017). A SLC39A8 Variant Causes Manganese Deficiency, and Glycosylation and Mitochondrial Disorders. J Inherit Metab Dis 40(2):261-269.
80. Perez Y et al. (2017). SLC30A9 Mutation Affecting Intracellular Zinc Homeostasis Causes a Novel Cerebro-Renal Syndrome. Brain 140(4):928-939.
81. Ben-Zeev B et al. (2003). Progressive Cerebellocerebral Atrophy: A New Syndrome With Microcephaly, Mental Retardation, and Spastic Quadriplegia. J Med Genet 40(8):e96.
82. Wang D et al. (2018). Glucose Transporter Type 1 Deficiency Syndrome. 2002 Jul 30 [Updated 2018 Mar 1]. In: Adam MP, Ardinger HH, Pagon RA, et al., editors. GeneReviews ® [Internet]. Seattle (WA): University of Washington, Seattle; 1993-2020.
83. Kuiper A et al. (2019). Movement Disorders and Nonmotor Neuropsychological Symptoms in Children and Adults With Classical Galactosemia. J Inherit Metab Dis 42(3):451-458.
84. Kaur P et al. (2019). Confirmation of a Rare Genetic Leukoencephalopathy Due to a Novel Bi-allelic Variant in RPIA. Eur J Med Genet 62(8):103708.
85. Wang D and De Vivo D. (2018). Pyruvate Carboxylase Deficiency. 2009 Jun 2 [Updated 2018 Mar 1]. In: Adam MP, Ardinger HH, Pagon RA, et al., editors. GeneReviews® [Internet]. Seattle (WA): University of Washington, Seattle; 1993-2020.
86. Sarper N et al. (2013). Mild Hemolytic Anemia, Progressive Neuromotor Retardation and Fatal Outcome: A Disorder of Glycolysis, Triose- Phosphate Isomerase Deficiency. Turk J Pediatr 55(2):198-202.
87. Castiglioni C et al. (2015). Pyruvate Dehydrogenase Deficiency Presenting as Isolated Paroxysmal Exercise Induced Dystonia Successfully Reversed With Thiamine Supplementation. Case Report and Mini-Review. Eur J Paediatr Neurol 19(5):497-503.
88. Debray F et al. (2008). Pyruvate Dehydrogenase Deﬁciency Presenting as Intermittent Isolated Acute Ataxia. Neuropediatrics. 39(1):20-3.
89. Mellick G et al. (2004). Late-Onset Presentation of Pyruvate Dehydrogenase Deﬁciency. Mov Disord. 19(6):727-9.
90. Head R et al. (2004). Pyruvate dehydrogenase deﬁciency presenting as dystonia in childhood. Dev Med Child Neurol. 2004 Oct;46(10):710-2.
91. Sharkia R et al. (2019). Clinical, Radiological, and Genetic Characteristics of 16 Patients With ACO2 Gene Defects: Delineation of an Emerging Neurometabolic Syndrome. J Inherit Metab Dis 42(2):264-275.
92. Maas R et al. (2016). SUCLA2 Deficiency: A Deafness-Dystonia Syndrome With Distinctive Metabolic Findings (Report of a New Patient and Review of the Literature). JIMD Rep 27:27-32.
93. Carrozzo R et al. (2016). Succinate-CoA Ligase Deficiency Due to Mutations in SUCLA2 and SUCLG1: Phenotype and Genotype Correlations in 71 Patients. J Inherit Metab Dis 39(2):243-52.
94. Bourgeron T et al. (1994). Mutation of the Fumarase Gene in Two Siblings With Progressive Encephalopathy and Fumarase Deficiency. J Clin Invest 93(6):2514-8.
95. [Ait-El-Mkadem](https://pubmed.ncbi.nlm.nih.gov/?term=Ait-El-Mkadem+S&cauthor_id=27989324) S et al. (2017). Mutations in MDH2, Encoding a Krebs Cycle Enzyme, Cause Early-Onset Severe Encephalopathy. Am J Hum Genet 100(1):151-159.
96. Hardies K et al. (2015). Recessive Mutations in SLC13A5 Result in a Loss of Citrate Transport and Cause Neonatal Epilepsy, Developmental Delay and Teeth Hypoplasia. Brain 138(Pt 11):3238-50.
97. Balaji P et al. (2014). An Interesting Case of Metabolic Dystonia: L-2 Hydroxyglutaric Aciduria. Ann Indian Acad Neurol 17(1):97-9.
98. Falk M et al., (2014). AGC1 Deficiency Causes Infantile Epilepsy, Abnormal Myelination, and Reduced N-Acetylaspartate. JIMD Rep 14:77-85.
99. Steenweg M et al. (2010). An Overview of L-2-hydroxyglutarate Dehydrogenase Gene (L2HGDH) Variants: A Genotype-Phenotype Study. Hum Mutat 31(4):380-90.
100. Tranchant C and Anheim M. (2016). Movement disorders in mitochondrial diseases. Rev Neurol (Paris). 172(8-9):524-529.
101. El-Hattab A et al. (2018) MELAS. 2001 Feb 27 [Updated 2018 Nov 29]. In: Adam MP, Ardinger HH, Pagon RA, et al., editors. GeneReviews® [Internet]. Seattle (WA): University of Washington, Seattle; 1993-2020.
102. Melone M et al. (2004) Revelation of a new mitochondrial DNA mutation (G12147A) in a MELAS/MERFF phenotype. Arch. Neurol. 61: 269-272.
103. Thorburn D et al. (2017). Mitochondrial DNAAssociated Leigh Syndrome and NARP. 2003 Oct 30 [Updated 2017 Sep 28]. In: Adam MP, Ardinger HH, Pagon RA, et al., editors. GeneReviews® [Internet]. Seattle (WA): University of Washington, Seattle; 1993-2020.
104. Cohen B et al. (2018). POLG-Related Disorders. 2010 Mar 16 [Updated 2018 Mar 1]. In: Adam MP, Ardinger HH, Pagon RA, et al., editors. GeneReviews® [Internet]. Seattle (WA): University of Washington, Seattle; 1993-2020.
105. El-Hattab A et al. (2018). MPV17-Related Mitochondrial DNA Maintenance Defect. 2012 May 17 [Updated 2018 May 17]. In: Adam MP, Ardinger HH, Pagon RA, et al., editors. GeneReviews® [Internet]. Seattle (WA): University of Washington, Seattle; 1993-2020
106. Lönnqvist T. (2018). Infantile-Onset Spinocerebellar Ataxia. 2009 Jan 27 [Updated 2018 Apr 19]. In: Adam MP, Ardinger HH, Pagon RA, et al., editors. GeneReviews® [Internet]. Seattle (WA): University of Washington, Seattle; 1993-2020.
107. Gai X et al. (2013). Mutations in FBXL4, Encoding a Mitochondrial Protein, Cause Early-Onset Mitochondrial Encephalomyopathy. Am J Hum Genet 93(3):482-95.
108. Antonicka H et al. (2010). Mutations in C12orf65 in Patients with Encephalomyopathy and a Mitochondrial Translation Defect. Am J Hum Genet 87(1):115-22.
109. Finsterer J and Zarrouk-Mahjoub S. (2017). Phenotypic Spectrum of DARS2 Mutations. J Neurol Sci 376:117-118.
110. Webb B et al. (2015). Novel, Compound Heterozygous, Single-Nucleotide Variants in MARS2 Associated With Developmental Delay, Poor Growth, and Sensorineural Hearing Loss. Hum Mutat 36(6):587-92.
111. Virdee M et al. (2019). Expanding the Phenotype: Neurodevelopmental Disorder, Mitochondrial, With Abnormal Movements and Lactic Acidosis, With or Without Seizures (NEMMLAS) Due to WARS2 Biallelic Variants, Encoding Mitochondrial Tryptophanyl-tRNA Synthase. J Child Neurol 34(12):778-781.
112. Liskova P et al. (2013). Novel OPA1 Missense Mutation in a Family With Optic Atrophy and Severe Widespread Neurological Disorder. Acta Ophthalmol 91(3):e225-31.
113. Arif B et al. (2013). A Novel OPA3 Mutation Revealed by Exome Sequencing: An Example of Reverse Phenotyping. JAMA Neurol 70(6):783-7.
114. Donkervoort S et al. (2019). MSTO1 Mutations Cause mtDNA Depletion, Manifesting as Muscular Dystrophy With Cerebellar Involvement. Acta Neuropathol 138(6):1013-1031.
115. Maas R et al., (2017). Progressive Deafness-Dystonia Due to SERAC1 Mutations: A Study of 67 Cases. Ann Neurol 82(6):1004-1015.
116. Ucar S et al. (2017). Previously Unreported Biallelic Mutation in DNAJC19: Are Sensorineural Hearing Loss and Basal Ganglia Lesions Additional Features of Dilated Cardiomyopathy and Ataxia (DCMA) Syndrome?. JIMD Rep 35:39-45.
117. Tranebjærg L. (2019). Deafness-Dystonia-Optic Neuronopathy Syndrome. 2003 Feb 6 [Updated 2019 Nov 21]. In: Adam MP, Ardinger HH, Pagon RA, et al., editors. GeneReviews® [Internet]. Seattle (WA): University of Washington, Seattle; 1993-2020.
118. Jobling R et al. (2015). PMPCA Mutations Cause Abnormal Mitochondrial Protein Processing in Patients With Non-Progressive Cerebellar Ataxia. Brain 138(Pt 6):1505-17.
119. Wortmann S et al. (2016). CLPB Deficiency. 2016 Nov 22. In: Adam MP, Ardinger HH, Pagon RA, et al., editors. GeneReviews® [Internet]. Seattle (WA): University of Washington, Seattle; 1993-2020.
120. Vill K et al. (2018). SACS Variants Are a Relevant Cause of Autosomal Recessive Hereditary Motor and Sensory Neuropathy. Hum Genet 137(11-12):911-919.
121. Pierson T et al. (2011). Whole-exome Sequencing Identifies Homozygous AFG3L2 Mutations in a Spastic Ataxia-Neuropathy Syndrome Linked to Mitochondrial m-AAA Proteases. PLoS Genet 7(10):e1002325.
122. Schneider S and Bhatia K. (2010). Rare Causes of Dystonia Parkinsonism. Curr Neurol Neurosci Rep 10:431–439.
123. Kovacs-Nagy R et al. (2018). HTRA2 Defect: A Recognizable Inborn Error of Metabolism With 3-Methylglutaconic Aciduria as Discriminating Feature Characterized by Neonatal Movement Disorder and Epilepsy-Report of 11 Patients. Neuropediatrics 49(6):373-378.
124. Scalais E et al. (2013). Early Myoclonic Epilepsy, Hypertrophic Cardiomyopathy and Subsequently a Nephrotic Syndrome in a Patient With CoQ10 Deficiency Caused by Mutations in Para -Hydroxybenzoate-Polyprenyl Transferase (COQ2). Eur J Paediatr Neurol 17(6):625-30.
125. Heeringa S et al. (2011). COQ6 Mutations in Human Patients Produce Nephrotic Syndrome With Sensorineural Deafness. J Clin Invest 121(5):2013-24.
126. Terraciano A et al. (2012). The use of muscle biopsy in the diagnosis of undefined ataxia with cerebellar atrophy in children. Eur J Paediatr Neurol. 16-222(3): 248–256.
127. Buhaş D et al. (2013). A Treatable New Cause of Chorea: Beta-Ketothiolase Deficiency. Mov Disord 28(8):1054-6.
128. Wojcik M et al. (2015). Beta-Ketothiolase Deficiency Presenting With Metabolic Stroke After a Normal Newborn Screen in Two Individuals. JIMD Rep 39:45-54.
129. [Yalçinkaya](https://pubmed.ncbi.nlm.nih.gov/?term=Yal%C3%A7inkaya+C&cauthor_id=11295802) C et al. (2001). Delayed-onset Dystonia Associated With 3-oxothiolase Deficiency. Mov Disord 16(2):372-5.
130. Heimer G et al. (2019). MECR-Related Neurologic Disorder. 2019 May 9. In: Adam MP, Ardinger HH, Pagon RA, et al., editors. GeneReviews® [Internet]. Seattle (WA): University of Washington, Seattle; 1993-2020.
131. Ozaki K et al. (2015). A Novel Mutation in ELOVL4 Leading to Spinocerebellar Ataxia (SCA) With the Hot Cross Bun Sign but Lacking Erythrokeratodermia: A Broadened Spectrum of SCA34. JAMA Neurol 72(7):797-805.
132. Di Gregorio E et al. (2014). ELOVL5 Mutations Cause Spinocerebellar Ataxia 38. Am J Hum Genet 95(2):209-17.
133. Cho K et al. (2018). Neurodegeneration in an adolescent with Sjogren-Larsson syndrome: a decade-long follow-up case report. BMC Medical Genetics 19:152
134. Nakhaei S et al. (2018). A New Case of Chanarin-Dorfman Syndrome With a Novel Deletion in ABHD5 Gene. Iran Biomed J 22(6):415-9.
135. Sanchez-Iglesias S et al. (2019). Celia’s encephalopathy and c.974dupG in *BSCL2* gene: a hidden change in a known variant. Neurogenetics. 20(2): 73–82.
136. Ito D et al. (2018) BSCL2-Related Neurologic Disorders/ Seipinopathy. 2005 Dec 6 [Updated 2018 May 24]. In: Adam MP, Ardinger HH, Pagon RA, et al., editors. GeneReviews® [Internet]. Seattle (WA): University of Washington, Seattle; 1993-2020.
137. McMillan H et al. (2018). Recessive Mutations in ATP8A2 Cause Severe Hypotonia, Cognitive Impairment, Hyperkinetic Movement Disorders and Progressive Optic Atrophy. Orphanet J Rare Dis 13(1):86.
138. Darling A et al. (2019). PLA2G6-associated Neurodegeneration: New Insights Into Brain Abnormalities and Disease Progression. Parkinsonism Relat Disord 61:179-186.
139. Synofzik M et al. (2014). PNPLA6 Mutations Cause Boucher-Neuhauser and Gordon Holmes Syndromes as Part of a Broad Neurodegenerative Spectrum. Brain 137(Pt 1):69-77.
140. Fiskerstrand T et al. (2010). Mutations in ABHD12 Cause the Neurodegenerative Disease PHARC: An Inborn Error of Endocannabinoid Metabolism. Am J Hum Genet 87(3):410-7.
141. Rattay T et al. (2019). FAHN/SPG35: A Narrow Phenotypic Spectrum Across Disease Classifications. Brain 142(6):1561-1572.
142. Hammer M et al. (2013). Mutations in GBA2 Cause Autosomal-Recessive Cerebellar Ataxia With Spasticity. Am J Hum Genet 92(2):245-51.
143. Mink J et al. (2013). Classification and Natural History of the Neuronal Ceroid Lipofuscinoses. J Child Neurol. 28(9): 1101–1105.
144. Homer V et al. (2015). Mental Retardation and Ataxia Due to Normotriglyceridemic Hypobetalipoproteinemia. Ann Neurol 58(1):160-3.
145. Burnett J et al. (2018). Abetalipoproteinemia. In: Adam MP, Ardinger HH, Pagon RA, et al., editors. GeneReviews® [Internet]. Seattle (WA): University of Washington, Seattle; 1993-2020
146. Kellner U et al. (2017). Mevalonate Kinase Deficiency Associated With Ataxia and Retinitis Pigmentosa in Two Brothers With MVK Gene Mutations. Ophthalmic Genet 38(4):340-344.
147. Vilarinho S et al. (2016). ACOX2 Deficiency: A Disorder of Bile Acid Synthesis With Transaminase Elevation, Liver Fibrosis, Ataxia, and Cognitive Impairment. Proc Natl Acad Sci U S A 113(40):11289-11293
148. Dick D et al. (2011). AMACR Mutations Cause Late-Onset Autosomal Recessive Cerebellar Ataxia. Neurology 76(20):1768-70.
149. Di Fabio R et al. (2014). Sensory Ataxia as a Prominent Clinical Presentation in Three Families With Mutations in CYP7B1. J Neurol. 2014 Apr;261(4):747-51.
150. Stelten B et al. (2019). Movement Disorders in Cerebrotendinous Xanthomatosis. Parkinsonism Relat Disord 58:12-16.
151. Jimenez-Jimenez F et al. (2013). Hereditary Coproporphyria Associated with the Q306X Mutation in the Coproporphyrin Oxidase Gene Presenting with Acute Ataxia. Tremor Other Hyperkinet Mov (N Y).3:tre-03-151-4162-1.
152. Mannino E et al. (2018). Congenital methemoglobinemia type II in a 5-year-old boy. Clinical Case Reports 6(1): 170–178
153. Kevelam S et al. (2016). Acute Intermittent Porphyria-Related Leukoencephalopathy. Neurology 87(12):1258-65.
154. Bryant D et al. (2018). SNX14 mutations affect endoplasmic reticulum associated neutral lipid metabolism in autosomal recessive spinocerebellar ataxia 20. Hum Mol Genet 27(11):1927-1940.
155. Hoffjan S et al. (2016). WDR45 Mutations in Rett (-Like) Syndrome and Developmental Delay: Case Report and an Appraisal of the Literature. Mol Cell Probes 30(1):44-9.
156. Behrens M et al. (2010). Clinical Spectrum of Kufor-Rakeb Syndrome in the Chilean Kindred With ATP13A2 Mutations. Mov Disord. 25(12):1929-37.
157. Hersheson J et al. (2014). Cathepsin D deficiency causes juvenile-onset ataxia and distinctive muscle pathology. Neurology.83(20):1873-5.
158. Bras J et al. (2016). Exome sequencing in a consanguineous family clinically diagnosed with early onset Alzheimer’s disease identifies an homozygous CTSF mutation. Neurobiol Aging. 46:236.e1–236.e6.
159. Smith et al. (2012). Strikingly Different Clinicopathological Phenotypes Determined by Progranulin-Mutation Dosage. Am J Hum Genet. 90(6):1102-7.
160. Zare-Abdollahi D et al. (2019). MFSD8 gene mutations; evidence for phenotypic heterogeneity. Ophthalmic Genet.40(2):141-145.
161. Chueng N et al. (2018). First case of genetically confirmed CLN3 disease in Chinese with cDNA sequencing revealing pathogenicity of a novel splice site variant. Clin Chim Acta 486:151-155.
162. Ferreira C and Gahl W. (2017). Lysosomal Storage Diseases. Transl Sci Rare Dis 2(1-2):1-71.
163. Yamanouchi H et al. (1991). Palatal Myoclonus in Krabbe Disease. Brain Dev 13(5):355-8.
164. Kuchař L et al. (2009). Prosaposin deficiency and saposin B deficiency (activator‐deficient metachromatic leukodystrophy): Report on two patients detected by analysis of urinary sphingolipids and carrying novel PSAP gene mutations. Am J Med Genet A 149A(4):613-21.
165. Schlotawa L et al. (2019). Multiple Sulfatase Deficiency. 2019 Mar 21. In: Adam MP, Ardinger HH, Pagon RA, et al., editors. GeneReviews® [Internet]. Seattle (WA): University of Washington, Seattle; 1993-2020.
166. Pattel M et al. (1999). Early-infantile Galactosialidosis: Prenatal Presentation and Postnatal Follow-Up. Am J Med Genet 85(1):38-47.
167. Gautschi M et al. (2014). Late Diagnosis of Fucosidosis in a Child With Progressive Fixed Dystonia, Bilateral Pallidal Lesions and Red Spots on the Skin. Eur J Paediatr Neurol 18(4):516-9.
168. Labauge et al. (2009). Beta-mannosidosis: A New Cause of Spinocerebellar Ataxia. Clin Neurol Neurosurg 111(1):109-10.
169. Malm D and Nilssen Ø. (2008). Alpha-mannosidosis. Orphanet J Rare Dis. 3:21.
170. Gowda V et al. (2017). Sialidosis Type 1 With a Novel Mutation in the Neuraminidase-1 (NEU1) Gene. Indian J Pediatr 84(5):403-404.
171. Patterson M. (2019). Niemann-Pick Disease Type C. 2000 Jan 26 [Updated 2019 Aug 29]. In: Adam MP, Ardinger HH, Pagon RA, et al., editors. GeneReviews® [Internet]. Seattle (WA): University of Washington, Seattle; 1993-2020.
172. Rubboli G et al., 2011. Clinical and neurophysiologic features of progressive myoclonus epilepsy without renal failure caused by *SCARB2*mutations. Epilepsia 52(12):2356-63.
173. Ebrahimi‐Fakhari D et al. (2017). The Spectrum of Movement Disorders in Childhood‐onset Lysosomal Storage Diseases. Version 2. Mov Disord Clin Pract. 5(2):149-155.
174. Chen Y et al. (2017). Unmasking adrenoleukodystrophy in a cohort of cerebellar ataxia. PLoS One 12(5):e0177296.
175. Chen Y et al. (2018). A Novel Variant in ABCD1 Gene Presenting as Adolescent-Onset Atypical Adrenomyeloneuropathy With Spastic Ataxia. Front Neurol 9:271.
176. Mason R et al. (2016). Early white matter involvement in an infant carrying a novel mutation in ACOX1. Eur J Paediatr Neurol 20(3):431-4.
177. Matsukawa T et al. (2017). Slowly progressive D-bifunctional protein deficiency with survival to adulthood diagnosed by whole-exome sequencing. J Neurol Sci 15;372:6-10.
178. Ferdinandusse S et al. (2006). Mutations in the Gene Encoding Peroxisomal Sterol Carrier Protein X (SCPx) Cause Leukencephalopathy With Dystonia and Motor Neuropathy. Am J Hum Genet 78(6):1046-52
179. Cakirer S and Savas M. (2004). Infantile Refsum disease: serial evaluation with MRI. Pediatr Radiol 35: 212–215
180. Tran C et al. (2014). Late-Onset Zellweger Spectrum Disorder Caused by PEX6 Mutations Mimicking X-Linked Adrenoleukodystrophy. Pediatr Neurol 51(2):262-5.
181. Zhang C et al. (2019). Ataxia With Novel Compound Heterozygous PEX10 Mutations and a Literature Review of PEX10-related Peroxisome Biogenesis Disorders. Clin Neurol Neurosurg 177:92-96.
182. Kumar K et al. (2018). Expanding the spectrum ofPEX16mutations and novel insights into disease mechanisms. Mol Genet Metab Rep. 20;16:46-51.
183. Würde A et al. (2012). Congenital disorder of glycosylation type Ij (CDG-Ij, DPAGT1-CDG): Extending the clinical and molecular spectrum of a rare disease. Mol Genet Metab 105(4):634-41.
184. Morava E et al. (2012). Defining the Phenotype in Congenital Disorder of Glycosylation Due to ALG1 Mutations. Pediatrics 130(4):e1034-9.
185. Quelhas D et al. (2019). RFT1-CDG: Absence of Epilepsy and Deafness in Two Patients with Novel Pathogenic Variants. J Inherit Metab Dis 43: 111–116.
186. Morava E et al. (2016). ALG6-CDG: A Recognizable Phenotype With Epilepsy, Proximal Muscle Weakness, Ataxia and Behavioral and Limb Anomalies. J Inherit Metab Dis 39(5):713-723.
187. Kobayashi Y et al. (2016). High prevalence of genetic alterations in early-onset epileptic encephalopathies associated with infantile movement disorders. Brain Dev 38(3):285-92.
188. Zhao J et al. (2017). Reduced Cell Surface Levels of GPI-linked Markers in a New Case With PIGG Loss of Function. Hum Mutat. 38(10):1394-1401.
189. Maydan G et al. (2011). Multiple congenital anomalies-hypotonia-seizures syndrome is caused by a mutation in PIGN. J Med Genet 48(6):383-9.
190. Kettwig M et al. (2016). Compound heterozygous variants in PGAP1 causing severe psychomotor retardation, brain atrophy, recurrent apneas and delayed myelination: a case report and literature review. BMC Neurology 16:74
191. Howard M et al. (2014). Mutations inPGAP3Impair GPI-Anchor Maturation,Causing a Subtype of Hyperphosphatasiawith Mental Retardation. Am J Hum Genet. 94(2):278-87.
192. Simpson M et al. (2004). Infantile-onset Symptomatic Epilepsy Syndrome Caused by a Homozygous Loss-Of-Function Mutation of GM3 Synthase. Nat Genet 36(11):1225-9.
193. Gordon-Lipkin E et al.(2018). ST3GAL5-Related Disorders: A Deficiency in Ganglioside Metabolism and a Genetic Cause of Intellectual Disability and Choreoathetosis. J Child Neurol 33(13):825-831.
194. Boukhris A et al. (2013). Alteration of Ganglioside Biosynthesis Responsible for Complex Hereditary Spastic Paraplegia. Am J Hum Genet. 93(1):118-23.
195. Kasapkara C et al. (2012). SRD5A3-CDG: A patient with a novel mutation. Eur J Paediatr Neurol 16(5):554-6.
196. Dancourt J et al. (2006). A New Intronic Mutation in the DPM1 Gene Is Associated With a Milder Form of CDG Ie in Two French Siblings. Pediatr Res 59(6):835-9.
197. Kranz C et al. (2001). A mutation in the human MPDU1 gene causes congenital disorder of glycosylation type If (CDG-If). J Clin Invest. 108(11): 1613–1619.
198. Larson A et al. (2018). TRAPPC11 and GOSR2 mutations associate with hypoglycosylation of α-dystroglycan and muscular dystrophy. Skeletal Muscle 8:17
199. Reynders E et al. (2009). Golgi function and dysfunction in the first COG4-deficient CDG type II patient. Hum Mol Genet 18(17):3244-56.
200. Kim Y et al. (2017). A Mild Form of COG5 Defect Showing Early-Childhood-Onset Friedreich's-Ataxia-Like Phenotypes with Isolated Cerebellar Atrophy. J Korean Med Sci. 32(11):1885-1890.
201. Foulquier F et al. (2007). A New Inborn Error of Glycosylation Due to a Cog8 Deficiency Reveals a Critical Role for the Cog1-Cog8 Interaction in COG Complex Formation. Hum Mol Genet 16(7):717-30.
202. Dibbens L and Rubboli G. (2017). GOSR2: A Progressive Myoclonus Epilepsy Gene. Epileptic Disord 18(S2):111-114.
203. Mostile G et al. (2019). Hyperkinetic movement disorders in congenital disorders of glycosylation. Eur J Neurol. 26(9):1226-1234.
204. Lam C et al. (2017). Prospective Phenotyping of NGLY1-CDDG, the First Congenital Disorder of Deglycosylation. Genet Med 19(2):160-168.
