# Supplementary material for: A Proposed Diagnostic Algorithm for Inborn Errors of Metabolism Presenting With Movements Disorders
Source: Front Neurol. 2020 Nov 13;11:582160. doi: 10.3389/fneur.2020.582160 (PMC7691570; doi:10.3389/fneur.2020.582160)
Supplement: Supplementary file 7 [file Table_7.DOCX]

**TABLE S2. Summary of the main characteristics of the reported studies regarding metabolic etiology in series of patients with movement disorders and metabolic etiology in children who attend pediatric emergency services.**

| References | Population / Number of patient | Type of genetic testing | Movement disorders | IEMs diagnosis | Diagnosis yield or % of IEMs |
| --- | --- | --- | --- | --- | --- |
| 3 | 51 children with MD | Targeted next generation sequencing panels conﬁrmed a genetic diagnosis of IEMs in 3 patients, whole exome sequencing identiﬁed a genetic diagnosis in 5 patients and mitochondrial genome sequencing in 1 patient. | Dystonia (49%) (generalized 84%, focal 12%, hemidystonia 4%), ataxia (53%), choreoathetosis (13%), tremor (16%), and hyperkinetic movements (13%). 45% had more than one MD in combination | GLUT1 deficiency, NCL2, *SURF1*-associated Leigh disease Pyruvate dehydrogenase complex (PDHC) deﬁciency, *DNAJC19*, mitochondrial encephalopathy due to ND3, riboﬂavin transporter type 2 deﬁciency (*SLC52A2*), mitochondrial 3-hydroxy-3-methylglutaryl (HMG) CoA synthase 2 deﬁciency (*HMGCS2*), PDHC E3 deﬁciency (*DLD*) | Diagnosis yield 51% |
| 29 | 61 patients (age range 1–73 years) | Targeted next-generation sequencing dystonia panel of 94 genes | All patients have dystonia | TH deﬁciency, glutaric aciduria type I, Niemann-Pick type C | Diagnosis yield 14,8% |
| 30 | 50 patients: 10 patients in the MD group (age range: 12 – 68 years old) and 7 patients in the mitochondrial group (4 of these patients presented some MD, age range 2-30 years old). | They assessed traditional Sanger-based sequencing vs. exome sequencing | MD group: Hereditary spastic paraplegia (58.8%), cerebellar ataxia (23.5%), and dystonia (17.6%).  Mitochondrial disease group: ataxia 25%, dystonia 75% | Movement disorder: *GOSR2*, *TWNK, ATP13A2,* and *VCP*  Mitochondrial disease group: *MTFMT, RARS2, NDUFA1* and *TUFM* | MD group: Diagnosis yield 5% vs. 20%  Mitochondrial disease group: Diagnosis yield 11% vs. 16% |
| 31 | 378 patients (age range: 0-84 years) | Targeted next generation sequencing panels (127 genes) | Parkinsonism (47.9%), dystonia (35.7%), chorea (6.6%), paroxysmal MD (5.3%) and myoclonus (4.5%)  Cerebellar ataxia: 23 patients | *MD cohort: GBA, PLA2G6, ATP13A2, GCH1, GLRA1, WDR45, HTRA2, ALDH5A1, SUOX, TIMM8A*  *Cerebellar ataxia cohort: PEX6, PEX10, SLC52A2, SACS, HSD17B4, SPG7,* | Diagnosis yield MD 22%  Diagnosis yield cerebellar ataxia 43,5% |
| 32 | 221 patients (<18 years: 72 cases, >18 years: 149 cases) | Targeted next generation sequencing panels (70 genes) | Not specified | *GLB1, GCH1, SPR, TH, WDR45, GRN, GBA, PRKN, PINK1* | Diagnosis yield 11,3% |
| 33 | 148 patients (age range 0–17 years) | Targeted next generation sequencing panels (102 genes) | Combined dystonia (40%), isolated dystonia (21%), paroxysmal MD (23%), chorea (13%), tremor (3%) | *ADAR1, PANK2, PLA2G6, WDR45, SLC2A1* | Diagnosis yield 28% |
| 36 | 52 children (age range: 2 months – 15 years) presenting with acute MD to the pediatric emergency department | Not commented | Chorea (38%), Dystonia (32%), Tremor (23%), Myoclonus (19%) and Parkinsonism (19%) | Glutaric aciduria type 1 and Leigh syndrome | % of IEMs: 5,7% |
| 35 | 92 children (age range: 5 days to 15 years) presenting with acute MD to the pediatric emergency department | Not commented | Hyperkinetic MD (82%): myoclonus (27%), dystonia (23%), choreoathetosis (20%), tremor (16%), tics (2%).  Acute parkinsonism (3%) | Glutaric aciduria type 1, Leigh syndrome, MELAS,  pantothenate kinase–associated neurodegeneration and unspecified leukodystrophy | % of IEMs: 8,6% |
| 34 | 256 children (age range 2months to 17 years) presenting with acute hyperkinetic MD to the pediatric emergency department | Not commented | Tics (44.5%), Tremor (21.1%), Chorea (13.7%), Dystonia (10.2%), Myoclonus (6.3%) and Stereotypies  (4.3%). | Ceroidolipofuscinosis and Mucopolisacaridosis | % of IEMs: 1,1% |
