# Supplementary material for: A Proposed Diagnostic Algorithm for Inborn Errors of Metabolism Presenting With Movements Disorders
Source: Front Neurol. 2020 Nov 13;11:582160. doi: 10.3389/fneur.2020.582160 (PMC7691570; doi:10.3389/fneur.2020.582160)
Supplement: Supplementary file 8 [file Table_8.DOCX]

**Table S3. Differential diagnosis of IEMs with disease-specific treatment**

| Ataxia | Dystonia |
| --- | --- |
| CAD trifunctional protein deficiency  Phosphoribosyl pyrophosphate synthetase 1 superactivity  Hypoxanthine guanine phosphoribosyltransferase deficiency  Guanidinoacetate methyltransferase deficiency  Creatine transporter deficiency  Glutathione synthetase deficiency, severe  Ornithine transcarbamylase deficiency  Argininosuccinate synthetase deficiency  Argininosuccinate lyase deficiency  Arginase deficiency  Mitochondrial ornithine transporter deficiency  Dopamine-serotonin vesicular transport defect  Classic homocystinuria  Ethylmalonic encephalopathy  Maple syrup urine disease  Isovaleric acidemia  Propionic acidemia  Methylmalonyl-CoA epimerase deficiency  Methylmalonic aciduria due to methylmalonyl-CoA mutase deficiency  Glutaric aciduria type 1  Glycine encephalopathy due to glycine decarboxylase deficiency  Glycine encephalopathy due to aminomethyltransferase deficiency  Imerslund-Gräsbeck syndrome  cblC disease  Methylcobalamin synthesis defect - cblD variant 1  Methionine synthase deficiency – cblG  Hereditary folate malabsorption  Folate receptor alpha deficiency  Methylenetetrahydrofolate reductase deficiency  Dihydrofolate reductase deficiency  Biotinidase deficiency  Holocarboxylase synthetase deficiency  Biotin-thiamine-responsive basal ganglia disease  Thiamine pyrophosphokinase deficiency  NAXE deficiency  Alpha-tocopherol transfer protein deficiency  Molybdenum cofactor deficiency  Wilson disease  Menkes disease  Aceruloplasminemia  SLC39A8 deficiency  Glucose transporter 1 deficiency  Classic galactosemia  Pyruvate dehydrogenase complex deficiency  COQ6 deficiency  COQ8A deficiency  Beta-ketothiolase deficiency  Celia's encephalopathy  Cerebrotendinous xanthomatosis  Recessive porphobilinogen deaminase deficiency  Coproporphyrinogen oxidase deficiency  CLN2 disease  Gaucher disease  Metachromatic leukodystrophy  Alpha-mannosidosis  X-linked adrenoleukodystrophy  Refsum disease  Zellweger spectrum disorders | Hypoxanthine guanine phosphoribosyltransferase deficiency  Guanidinoacetate methyltransferase deficiency  Creatine transporter deficiency  Tyrosine hydroxylase deficiency  Aromatic L-amino acid decarboxylase deficiency  Dopamine transporter deficiency  Dopamine-serotonin vesicular transport defect  Autosomal recessive GTP cyclohydrolase I deficiency  Autosomal dominant GTPCH deficiency  6-Pyruvoyl-tetrahydropterin synthase deficiency  Sepiapterin reductase deficiency  DNAJC12-deficient hyperphenylalaninemia  Classic homocystinuria  Ethylmalonic encephalopathy  Maple syrup urine disease  Propionic acidemia  Methylmalonic aciduria due to methylmalonyl-CoA mutase deficiency  Malonic aciduria  Glutaric aciduria type 1  Methylcobalamin synthesis defect - cblD variant 1  Biotin-thiamine-responsive basal ganglia disease  Thiamine pyrophosphokinase deficiency  Mitochondrial thiamine pyrophosphate transporter deficiency  Pyridoxine-dependent epilepsy  PNPO deficiency  Molybdenum cofactor deficiency  Wilson disease  Aceruloplasminemia  SLC30A10 deficiency  SLC39A14 deficiency  SLC39A8 deficiency  Glucose transporter 1 deficiency  Classic galactosemia  Pyruvate dehydrogenase complex deficiency  COQ8A deficiency  Beta-ketothiolase deficiency  Celia's encephalopathy  Cerebrotendinous xanthomatosis  CLN2 disease  Gaucher disease  Metachromatic leukodystrophy  X-linked adrenoleukodystrophy |
| Choreoathetosis | **Tremor** |
| Hypoxanthine guanine phosphoribosyltransferase deficiency  Guanidinoacetate methyltransferase deficiency  Creatine transporter deficiency  Aromatic L-amino acid decarboxylase deficiency  Dopamine transporter deficiency  6-Pyruvoyl-tetrahydropterin synthase deficiency  Sepiapterin reductase deficiency  Dihydropteridine reductase deficiency  Propionic acidemia  Methylmalonic aciduria due to methylmalonyl-CoA mutase deficiency  Glutaric aciduria type 1  Glycine encephalopathy due to glycine decarboxylase deficiency  Glycine encephalopathy due to aminomethyltransferase deficiency  cblX disease  Hereditary folate malabsorption  Classic galactosemia  Folate receptor alpha deficiency  Molybdenum cofactor deficiency  Wilson disease  Aceruloplasminemia  Glucose transporter 1 deficiency  Pyruvate dehydrogenase complex deficiency  Beta-ketothiolase deficiency  Cerebrotendinous xanthomatosis  CLN2 disease | Tyrosine hydroxylase deficiency  Aromatic L-amino acid decarboxylase deficiency  Dopamine transporter deficiency  Phenylketonuria  Autosomal recessive GTP cyclohydrolase I deficiency  Autosomal dominant GTPCH deficiency  6-Pyruvoyl-tetrahydropterin synthase deficiency  Sepiapterin reductase deficiency  Maple syrup urine disease  Isovaleric acidemia  Folate receptor alpha deficiency  Pyridoxine-dependent epilepsy  Wilson disease  Aceruloplasminemia  SLC30A10 deficiency  SLC39A14 deficiency  Glucose transporter 1 deficiency  Classic galactosemia  COQ2 deficiency  COQ8A deficiency  Beta-ketothiolase deficiency  Celia's encephalopathy  Cerebrotendinous xanthomatosis  Gaucher disease |
| Myoclonus | **HRS** |
| Mitochondrial ornithine transporter deficiency  Tyrosine hydroxylase deficiency  Aromatic L-amino acid decarboxylase deficiency  Sepiapterin reductase deficiency  Classic homocystinuria  Pyridoxine-dependent epilepsy  Wilson disease  Glucose transporter 1 deficiency  COQ2 deficiency  COQ8A deficiency  Celia's encephalopathy  Cerebrotendinous xanthomatosis  CLN2 disease  Gaucher disease | Tyrosine hydroxylase deficiency  Aromatic L-amino acid decarboxylase deficiency  Dopamine transporter deficiency  Dopamine-serotonin vesicular transport defect  Phenylketonuria  Autosomal dominant GTPCH deficiency  Sepiapterin reductase deficiency  Dihydropteridine reductase deficiency  DNAJC12-deficient hyperphenylalaninemia  Glutaric aciduria type 1  Molybdenum cofactor deficiency  Wilson disease  Aceruloplasminemia  SLC30A10 deficiency  SLC39A14 deficiency  Glucose transporter 1 deficiency  Classic galactosemia  Cerebrotendinous xanthomatosis  Gaucher disease |
