# Supplementary material for: A Proposed Diagnostic Algorithm for Inborn Errors of Metabolism Presenting With Movements Disorders
Source: Front Neurol. 2020 Nov 13;11:582160. doi: 10.3389/fneur.2020.582160 (PMC7691570; doi:10.3389/fneur.2020.582160)
Supplement: Supplementary file 9 [file Table_9.DOCX]

| Table S4. Summary of the main characteristics of the reported studies of deep brain stimulation in children with inborn error of metabolism | | | | | |
| --- | --- | --- | --- | --- | --- |
| Reference | Age at MD onset | Age at DBS | IEMS | DBS target | Outcome |
| 110 | 6y | 18 y | X-Linked Adrenoleukodystrophy | Gpi | After 25 months, the patient had neither deteriorated nor shown improvement of dystonia. |
| 111 | 21 months | 4y | Methylmalonic acidemia | STN | Follow-up at 6 months: Clinical improvements were not matched by changes in BFMDRS scores: interacting and more smiling, diapers change easier |
| 112 | 6y | 7y | Methylmalonic acidemia | Thalamic | Follow-up at 12 months: No change in BFMDRS scores |
| 113 | 3 y | 16 y | Mitochondrial disorder | Gpi | Follow-up at 12 months: SF-36 scale worsening in 59%, BFMDRS worsening in 19% and UMRS worsening in 23%. The frequency and intensity of action myoclonus scores remained lower when compared to baseline scores. |
| 114 | 4y 8 m | 10 y | Thiamine pyrophosphokinase deficiency | Gpi | Little benefit |
| 115 | 10y | 15y | Lesch–Nyhan syndrome | Gpi | Follow-up at 24 months: no self-mutilating behavior. BFMDRS improvement 33% movement section and 50% in disability section. |
| 116 | 2 patients (both onset at 1y) | 12 y | Lesch–Nyhan syndrome | Gpi | Follow-up at 3 months: no self-mutilating behavior. Decreased of dystonia (no scale available) |
| 117 | 4 patients (not available) | Not available | Lesch–Nyhan syndrome | Not available | Initial improvement of dystonia and behavior in 2 of them, but they all worsened signiﬁcantly so that the treatment was stopped and subsequently 3 of them died. |
| 118 | 1 y | 8 y | Lesch–Nyhan syndrome | Gpi | Follow-up at 2,5 years: no self-mutilating behavior. Barry-Albright Dystonia Scale improves in 50% |
| 119 | 6 months | 16 y | Lesch–Nyhan syndrome | Antroventral globus pallidus | Follow-up at 16 months: improvement in Behavior Problems Inventory (BPI-1) questionnaire, movement part of the BFMDRS remains unchanged |
| 120 | 6 months | 15 y | Lesch–Nyhan syndrome | Gpi | Follow-up at weeks: dystonia and self-injurious behavior improved (no scale available) |
| 121 | 5,4 to 16,8 y | Not available | Glutaric aciduria type 1 (1), Lesch–Nyhan syndrome (1), NBIA (3), PINK1 (1) | Gpi, except PINK1 (STN) | Follow-up: mild to no improvement in Glutaric aciduria type 1, Lesch–Nyhan syndrome and *PINK1* patients. BFMDRS progressive worsening in NBIA patients |
| 122 | 4 patients (age range:8y to 16y) | 1 y to 8 y | Pantothenate kinase associated neurodegeneration | Gpi (1) and STN (3) | Follow-up: BFMDRS scores improves between 7 to 45%. |
| 123 | 6y | 13y | Pantothenate kinase associated neurodegeneration | Gpi | Follow-up at 12 months: motor BFMDRS score improves 32%, but motor BFMDRS score gradually deteriorate during the following years |
| 124 | 3 children (age at onset: 1-10y) | 10y to 13y | Pantothenate kinase associated neurodegeneration | Gpi | Global motor improvement measure by motor BFMDRS score: 46% to 91,5%. |
| 125 | 2,5y | 11y | Pantothenate kinase associated neurodegeneration | Gpi | 33% improvement on Barry–Albright dystonia scale. Removal of DBS due to infection, slow deterioration to presurgical condition |
| 126 | 7 patients (range at onset 6 months to 8y) | Range 8y-17y | Pantothenate kinase associated neurodegeneration | Gpi | Patient 7 do not respond to DBS. |
| 127 | 11y | 15y | Pantothenate kinase associated neurodegeneration | STN | Follow-up at 44 months: axial (eye, mouth, speech and swallowing, neck and trunk) BFMDRS score improvement of 27,3% and appendicular (arms and legs) BFMDRS score improvement of 91,7% |
| 128 | 8y | 6y | Pantothenate kinase associated neurodegeneration | Gpi | Patient died 3 months after the procedure |
| 129 | 9y | 17y | Pantothenate kinase associated neurodegeneration | Gpi | Follow-up: 16% improvement in motor BFMDRS score |
| 130 | 10y | 16y | Pantothenate kinase associated neurodegeneration | Gpi | Follow-up at 8 months: 27,5% improvement in motor BFMDRS score |
| 131 | 15 patients (age at onset 7,8±4,8y) | 18±8,8 y | 60,9% Pantothenate kinase associated neurodegeneration, 34,8% not tested | Gpi | The mean improvement in severity of dystonia was 28.5% at 2–6 months and 25.7% at 9-15 months measure by BFMDRS score |
| 132 | 12y | 16y | Pantothenate kinase associated neurodegeneration | STN | Follow-up at 3 years: 84% improvement in motor BFMDRS score |
| 133 | 2 patients (9,8y and 14,9y) | Not available | Pantothenate kinase associated neurodegeneration | Gpi | There was not improvement in motor BFMDRS score in both patients. The first patient died at 15,8 y. The other patient was followed up until age 18 y |
| 134 | 2 siblings (4y and 4,5 y) | 17,5 y and 16,5y | Pantothenate kinase associated neurodegeneration | Gpi | Follow-up at 4 years: 28 and 51% improvement in motor BFMDRS score |
