# Supplementary material for: A Proposed Diagnostic Algorithm for Inborn Errors of Metabolism Presenting With Movements Disorders
Source: Front Neurol. 2020 Nov 13;11:582160. doi: 10.3389/fneur.2020.582160 (PMC7691570; doi:10.3389/fneur.2020.582160)
Supplement: Supplementary file 10 [file Table_10.DOCX]

**Search strategy**

This review follows the recommendations of the 2009 guidelines for the systematic review of preferred reporting items for systematic review and meta-analysis (PRISMA). PubMed (<https://pubmed.ncbi.nlm.nih.gov/>) was systematically searched from 1960 to May 2020. The following search strategy was used: (“Metabolism, Inborn Errors” OR “Metabolic disease”) AND (“Paroxysmal dyskinesia” OR “Chorea” OR “Dystonia” OR “Tremor” OR “Ataxia” OR “Parkinsonism” OR “Myoclonus” OR “Stereotypies” OR “Tics” OR “Tourette syndrome” OR “Spastic paraparesis” OR “Movement disorder”). The search also includes disease-specific chapters in Genereviews (<https://www.ncbi.nlm.nih.gov/books/NBK1116/>).

In the event of MD in the neonatal period, the Age criteria: Neonate: Newborn – 1 month was selected in PubMed. Regarding DBS, the search was conducted through this strategy: (“Metabolism, Inborn Errors” OR “Metabolic disease”) AND (“Deep brain stimulation”).

**Selection criteria**

Eligible literature was included if they simultaneously fulfilled the predefined criteria. These studies 1) were prospective cohort studies or clinical cases in which the participants younger than 18 years old present any movement disorder and suffered one of the IEMs included in the classification of Ferreira et al., 2019. Studies were excluded if they did not meet the inclusion criteria. Nonoriginal research (reviews, commentaries, conference abstracts and editorial) and duplicated studies were excluded. If >1 article was found to have used the same data, the one with higher quality score was chosen, and where the score was equal, the study with the larger sample size was chosen. If there was more than one article on the same disease, preference was given to the article that contained a more detailed description of the MD. In some IEM in which different MD phenotypes were described, more than one article per IEM has been included.

**Data extraction and quality assessment**

The following information was extracted from each article: name of the first author, publication year, MD phenotype and other clinical features, biochemical and radiological data (CT, brain MRI and spectroscopy) and disease-specific treatment. The information obtained from this search is presented in **Supplementary Table 1** following the classification of Ferreira et al., 2019 available at doi:10.1016/j.ymgme.2019.03.007..
